# Butterfly wing patterns in flight create powerful illusory motion cues

**DOI:** 10.1038/s41586-026-11062-w

**Published:** 2026-09-30

**Authors:** George R. A. Hancock, Emmanuelle S. Briolat, Anna E. Hughes, Laura A. Kelley, Jolyon Troscianko

**Affiliations:** 1 Centre for Ecology and Conservation, University of Exeter, Penryn, UK; 2 Department of Psychology, University of Essex, Colchester, UK

**Keywords:** Behavioural ecology, Evolutionary ecology

## Abstract

The dazzling stripes found on zebras and snakes have long been thought to interfere with predator motion perception^1,2^, but this hypothesis has received little empirical support so far^3^. Butterflies show enormously diverse and high-contrast wing patterns, the function of which has inspired intense theoretical debate and research^4^. Here we suggest that butterfly wing patterns work in tandem with flight dynamics to create visual illusions that confuse predators. We use multiple lines of enquiry with biologically inspired modelling to support this hypothesis: free-flight butterfly take-offs filmed at high speed demonstrate the creation of misleading motion cues; phylogenetically controlled analyses demonstrate that this is an evolutionarily widespread strategy; in silico evolution experiments independently converge on high-contrast butterfly-like wing patterns; and behavioural experiments validate model predictions. Our results show that butterfly wing patterns and flight dynamics create powerful illusory motion effects, potentially representing one of the most successful visual defence strategies in moving animals.

## Abstract (web summary)

Butterfly wing patterns and flight dynamics work in tandem to create powerful visual illusions that confuse predators, potentially representing one of the most successful visual defence strategies in moving animals.

## Main

Movement can break even the most effective camouflage^5–8^, rendering almost any animal highly conspicuous. Animal markings have evolved to exploit limitations in the vision of their predators, and this principle should hold true for animals in motion, where patterns have long been thought to interfere with a predator’s perception of the speed and direction of their prey via ‘motion dazzle’ effects^3,9^. High-contrast stripes and patterns are known to create illusory motion^8,10,11^, but studies testing their effects in animals such as zebras, snakes and lizards have been somewhat equivocal in their support^1,2,12–14^. Importantly, these studies exclusively used rigid prey, despite animal locomotion often requiring limb movements that oppose the body’s main direction of travel. Biological motion, therefore, introduces unexplored potential for visual interference that is potentially common in nature^3,15^.

The striking coloration of butterflies has captivated the attention of evolutionary biologists for well over a century, making them one of the most studied groups in terms of how their appearance is linked to their behaviour and evolution^4^. Research has focused on the adaptive significance of warning signals, mimicry, sexual signals, thermoregulation and camouflage, and the underlying genetics and evolution^16^. However, it is less clear how these fundamental adaptive strategies can explain the high-contrast dorsal wing patterns seen in many butterflies. We propose that another, non-mutually exclusive, key function of butterfly wing patterns is anti-predator defence through creating illusory motion. There are multiple factors that suggest flight behaviour and wing patterning may interact to create misleading motion cues in butterflies, as many species have high-contrast patterns similar to those suggested to affect motion perception, as well as low wingbeat frequencies that are resolvable by avian vision^17^. Butterflies also often display unpredictable flight behaviour, which may interact with high-contrast markings and hindwing tails to deflect predatory attacks^18–20^. Furthermore, strikingly few predators have specialized on catching butterflies on the wing despite their extreme conspicuousness^21^. This is in contrast to nocturnal moths, which are targeted by a wide range of avian predators when in flight, and generally have lower-contrast patterns and high wingbeat frequencies that would probably produce too much motion blur to create illusory motion cues^22–25^.

## Motion-confusion measures

Elementary motion detectors (EMDs) form the basic unit of the motion processing system across a wide range of taxa^26,27^. We quantified motion detection in consecutive video frames as the mean EMD motion ‘energy’ in four cardinal directions (forwards, backwards, left and right)^28^, and defined motion confusion in two directions: forwards confusion (the Michelson contrast between forwards energy and backwards energy), and sideways confusion (the Michelson contrast between the mean left–right energy and forwards energy)^29^. Forwards confusion is likely to interfere with a predator’s estimation of speed (for example, misdirecting attacks behind a butterfly), whereas sideways confusion is likely to misdirect attacks laterally.

## Butterfly patterns create motion confusion

**Fig. 1:**
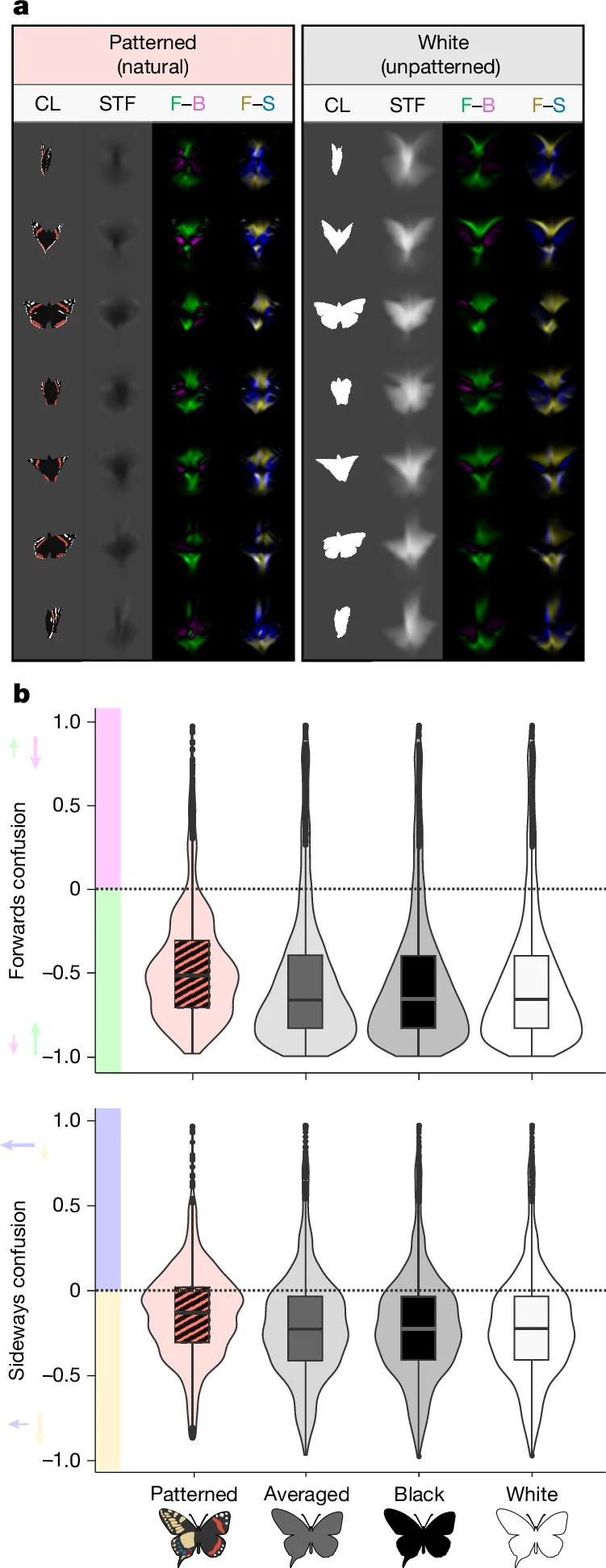
EMD measures for butterfly slow motion take-off. **a**, Frames from free-flight motion-confusion analysis using a red admiral butterfly, *Vanessa atalanta*, as an example with frames re-centred to match time, so spatial location is only approximate. From left to right, panels show the colour-segmented butterfly (CL), the butterfly converted to avian double-cone vision with spatiotemporal filtering (STF), the EMD model output for forwards confusion (F–B; Michelson contrast of backwards–forwards EMD), and then sideways confusion (F–S; Michelson contrast of sideways–forwards EMD). This process is repeated for an unpatterned butterfly where the wing coloration has been replaced by white. **b**, Boxplots (lower, median and upper quartiles, whiskers show maximum and minimum up to 1.5× interquartile range beyond the box) with violin distribution plots for forwards confusion (top) and for sideways confusion (bottom) across all frames for 18 flights for 7 butterfly wing morphotypes with patterning and without patterning (averaged, black and white), *N* = 2,391 per pattern treatment. Values greater than zero indicate when motion in the wrong direction (backwards or sideways) outweighs motion in the direction of heading, namely, forwards. Illustrations of treatments were created by G.R.A.H.

First, we experimentally tested whether the natural wing patterns maximized illusory motion cues in free flight compared with unpatterned treatments. This was tested by filming butterfly take-offs at 1,000 frames per second and segmenting the footage to manipulate and test how patterning affects early-stage motion detection (Supplementary Video 1). Butterflies with natural wing patterning created significantly more motion confusion when flying than experimentally unpatterned treatments (Tukey post hoc comparisons for patterned treatments compared with unpatterned, forwards confusion: average grey *β* = 0.079, *P* < 0.001; black *β* = 0.078, *P* < 0.001; white *β* = 0.078, *P* < 0.001; sideways confusion: average grey *β* = 0.044, *P* < 0.001, black *β* = 0.044, *P* < 0.001, white *β* = 0.044, *P* < 0.001; Fig. 1 and Supplementary Tables 1 and 2). In line with predictions of EMDs, higher contrast (being lighter or darker against the background) resulted in higher EMD values in all directions. However, patterning influenced the ratio of energy in different directions, creating both forwards- and sideways-confusion effects by reducing the relative amount of forwards motion.

## Motion confusion across European species

Next, we determined the properties of natural wing patterns that maximize motion-confusion effects and explored whether they are evolutionarily widespread. We rendered the simulated flight (both flapping and gliding) of 757 unique butterfly morphotypes across 397 European species at 2,000 frames per second to achieve fluid motion; Supplementary Video 2). European butterflies provide an ideal system because there are no major mimicry rings (comparatively few are chemically defended)^30^, yet many have otherwise unexplained high-contrast markings, and there is a detailed phylogeny available^31,32^.

Using random forest models^33^, a decision-tree-based method for identifying variable contributions to an output, we identified which butterfly features (73 different metrics for wing pattern (orientation, size, contrast), colour, shape, body size, as well as three principal coordinates for the phylogeny; see Supplementary Information, section 2 and Methods for full list) best explained our motion-confusion metrics for flapping flight (forwards-confusion model, pseudo-*R*^2^ = 0.65; sideways-confusion model, pseudo-*R*^2^ = 0.69; forwards-energy model, pseudo-*R*^2^ = 0.90). During flapping flight, forwards confusion was most effectively maximized through (in order of importance, with all highly influential): increased forewing contrast, resolvable vertical stripes on the hindwing, uneven stripe distribution (generally meaning a single stripe, either relatively central across both fore- and hindwing, or a contrasting edge), higher hindwing luminance, higher hindwing shape roughness (for example, hindwing tails), and vertical stripes on the forewing (see Supplementary Information, section 2(vi) for the full list of predictors). Sideways confusion similarly increased with vertical patterning and hindwing tails but was less affected by contrast and also increased with butterfly size. As with the take-off analyses, being whiter increased forwards energy (see Supplementary Information, section 1 for energy plots).

**Fig. 2:**
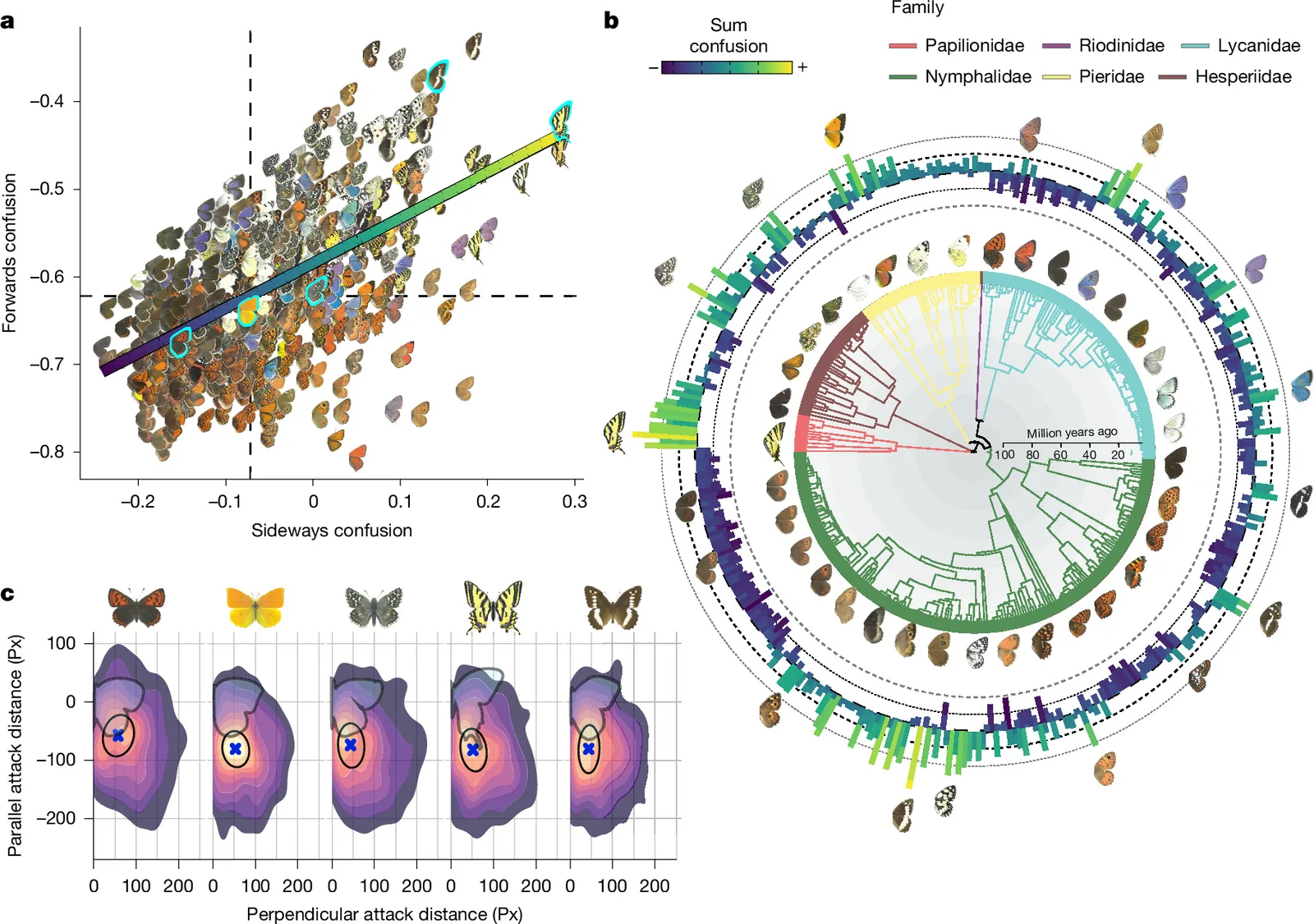
Motion confusion across European butterflies. **a**, Correlation between forwards confusion and sideways confusion within flapping European butterflies, *N* = 757. Each image corresponds to the motion-confusion measures for the shown butterfly. Butterflies highlighted in blue are those selected for the butterfly capture task (see **c**). **b**, Phylogeny of European butterflies with the normalized sum of forwards confusion and sideways confusion across the phylogeny: the height of each bar corresponds to the level of motion confusion relative to the mean. The thick dashed line shows the mean, and the lighter dashed lines indicate 1× and 2× the standard deviation from the mean. Outer wing images show the butterfly with the highest motion confusion at 20° intervals. Inner wing images show randomly selected example butterflies from within a 10° interval range. **c**, Location of attacks by human ‘predators’ on five selected butterfly phenotypes, ordered by forwards confusion. Heatmaps show the two-dimensional density estimates for attacks oriented relative to the heading of the butterflies, *N* = 2,985 attacks. Crosses and ellipses show the shape statistics for the 25% densest regions of each butterfly attack, with the crosses indicating the centroid and the ellipses showcasing the orientation, size and aspect ratio. Butterfly outlines showcase the to-scale butterfly when the wings were fully extended during flight. Illustrations in **a**,**b**,**c** from the *Collins Butterfly Guide* by Richard Lewington.

Motion confusion varied across butterfly taxa (Fig. 2a,b); for example, after controlling for phylogenetic relatedness using principal coordinates, Papilionidae consistently showed high forwards and sideways confusion, with all species measured possessing contrasting striped wing patterns, whereas Nymphalidae varied substantially from the highest overall forwards confusion (typically within species that possessed a single vertical stripe) to negligible effects. Forwards and sideways confusion were correlated with one another (linear model for forwards confusion against sideways confusion: *β* = 0.518, *P* < 0.001; Fig. 2a). Comparisons between flapping and gliding flight that isolate the effect of biological wing motion from wing pattern and shape showed that forwards confusion can only be created by wing motion, whereas gliding flight is still able to generate powerful sideways confusion. This may explain why Papilionidae (a group that glides during flight) have wing patterns that maximize both forwards confusion and sideways confusion (Fig. 2a).

## Motion confusion and human misdirection

We validated motion-confusion model predictions by measuring attack misdirection in human ‘predators’ (100 participants, 3,000 trials), using a high-speed touch-screen system (240 Hz) to present realistic butterfly flight trajectories and wing dynamics, using videos that were pre-processed to remove temporal aliasing artefacts. Five butterfly species were selected from across the confusion continuum (Fig. 2a), and those with high levels of forwards confusion resulted in attack distributions being more elongated and backwards along the parallel axis (forwards confusion:parallel axis, *β* = −0.15, *P* < 0.001; Fig. 2c; see Supplementary Table 14 and Supplementary Video 4 for full results and demonstration), in line with expectations for patterns that bias attacks towards the back of an object, that is, manipulating perception of speed.

## Digital evolution for motion confusion

**Fig. 3:**
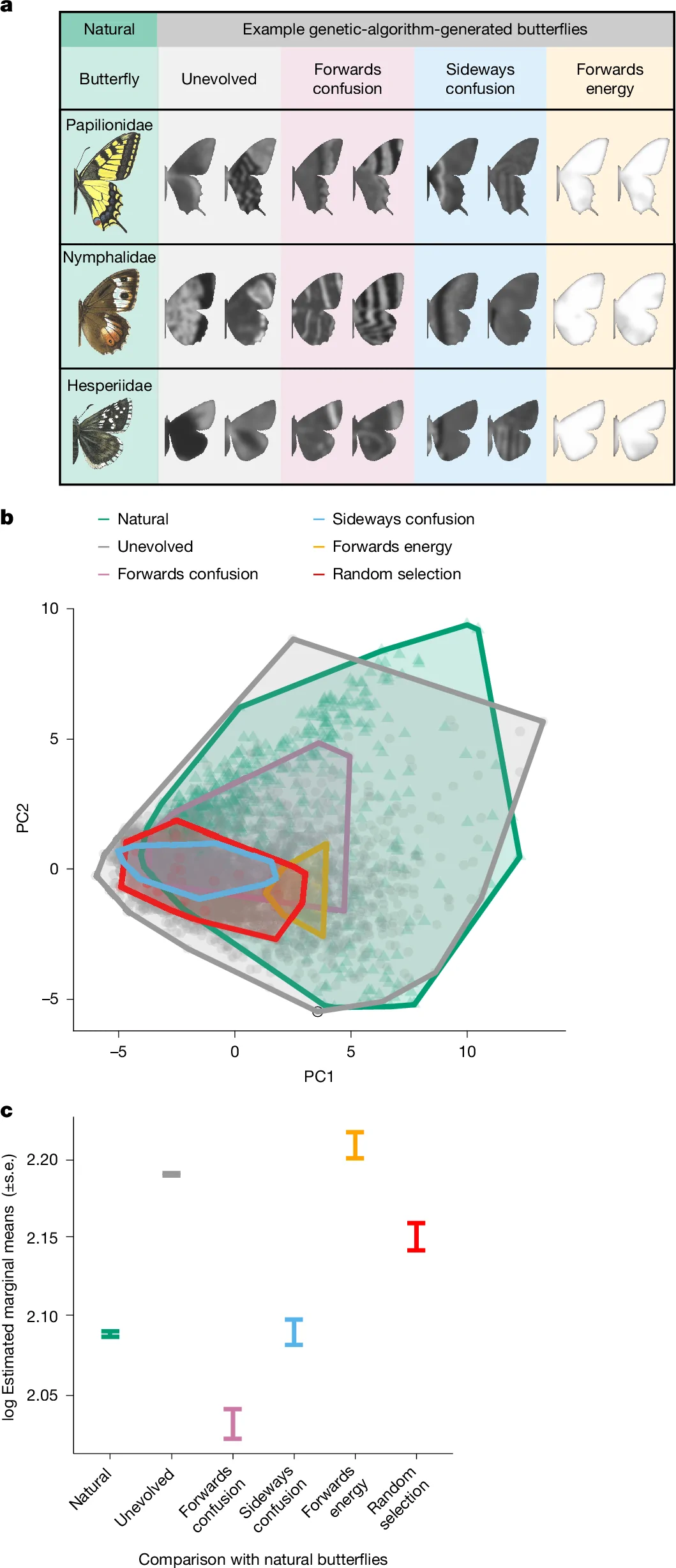
Artificial evolution of motion-confusing butterflies. **a**, Butterfly examples created using the genetic algorithm for three different wing shapes and the ‘natural’ pattern for the given wing shape (for examples from each population, see Supplementary Information, section 3). Unevolved butterflies were generated by using random gene values, whereas forwards confusion, sideways confusion and forwards energy were evolved under selection for their named metric with examples being from the final generation (*G* = 20). **b**, Wing pattern measure principal component analysis hulls for the 4 genetic-algorithm butterfly groups (unevolved *N* = 2,160, for all others *N* = 30 per group) and real, naturally occurring butterflies (*N* = 757). Here principal components (PCs) summarize variation in the butterfly wing pattern space, using the same metrics as used for the forest plots for the real butterfly wing patterns (Fig. 2). Random-selection butterflies evolved by randomly ranking the butterflies in each generation. **c**, The estimated marginal means for the pairwise distance of each group from natural butterflies, including the typical difference between natural butterflies (natural versus natural *N* = 572,292, unevolved versus natural *N* = 2,180,160, evolved groups versus natural *N* = 22,710). Error bars show the standard errors from the Tukey post hoc comparisons between the genetic-algorithm butterflies and the natural butterflies. Illustrations in **a** from the *Collins Butterfly Guide* by Richard Lewington.

Finally, we used genetic algorithms to evolve 57,600 unique butterfly wing patterns in silico, selecting for maximal motion confusion, free from evolutionary and developmental constraints of true butterfly wing patterns^4^ (Supplementary Video 3), and tested for pattern convergence with real natural butterfly wings. The genetic-algorithm-evolved butterfly patterns that were selected for either forwards confusion, sideways confusion or forwards energy (that is, the absolute motion energy in the forwards direction), replicated across three wing shapes (Fig. 3a). As with real butterflies, forwards and sideways confusion significantly correlated (linear model for sideways confusion against forwards confusion for genetic-algorithm-generated butterflies; sideways confusion, *β* = 0.733, *P* < 0.001).

Analysis of the evolved phenotypes revealed convergence between genetic-algorithm-evolved wing patterns selected for forwards confusion and those of European butterflies. Patterns that produced higher motion confusion had a lower pairwise Euclidean distance of principal components 1 (22% of variance) and 2 (8% of variance) from natural butterflies compared with butterflies randomly generated at the start (unevolved) (Fig. 3b), butterflies evolved under random selection (drift) or even outlying butterfly species (Fig. 3c; Tukey post hoc comparisons of natural butterflies to genetic-algorithm-generated butterflies compared; unevolved, *β* = −0.102, *P* < 0.001; forwards confusion, *β* = 0.056, *P* < 0.001; sideways confusion, *β *= 0.00, *P* = 0.999; forwards energy, *β* = −0.122, *P* < 0.001; random selection, *β* = −0.062, *P* < 0.001; see Supplementary Information, section 3 for the full table). Selecting for forwards energy resulted in immaculate white butterflies, consistent with the strategy adopted in the Pieridae (‘whites’), although they were more different from natural butterflies than other treatments. This strategy would be expected to also cause predators to overestimate forwards speed and over-shoot their attacks.

## Summary and implications

We provide empirical evidence that many butterfly wing patterns are consistent with selection for motion confusion likely to misdirect predator attacks in flight. Our study uses converging evidence from four biologically inspired frameworks; motion vision modelling, three-dimensional butterfly flight dynamics, behavioural validation and genetic algorithms. Taken together, these demonstrate how motion confusion could operate from mechanistic to evolutionary levels in a system long used as a testbed for evolutionary theory. We show that butterfly motion confusion can be achieved through multiple routes, with contrasting vertical forewing wing stripes, a singular vertical band or internal contrasting wing margin patterns all increasing motion confusion, whereas immaculate white maximizes forwards energy. Critically, the patterns that cause motion confusion are not mutually exclusive to—and may be complementary to—many of the other proposed adaptive functions of butterfly wing patterns such as signalling and thermoregulation^16,21,34^.

Our research goes beyond previous motion-illusion literature by controlling for temporal aliasing artefacts that may have limited previous findings^28,35^. We show that these confusion effects are probably widespread in a taxon that is both phenotypically and evolutionarily diverse. Our results offer empirical support for longstanding ‘motion dazzle’ hypotheses, and we predict that this type of motion confusion could offer an adaptive function for an enormous range of high-contrast patterns found in nature, such as those on the flapping wings of birds or the flicking tails of lizards and fishes^13,36,37^. These findings also highlight the importance of studying the adaptive function of animal patterning in tandem with realistic movement, as only together do the striking effects we observe here emerge.

EMD-based motion vision has been well validated^2,38–40^ and is increasingly being used to investigate animal patterns during movement^2,28,41^. Previous behavioural studies show that motion confusion from patterning can influence perceived direction^42,43^ and speed, for example, a 1.5° s^−1^ decrease in perceived speed for fast-moving targets^44^. Here we show that the forwards-confusion metric can meaningfully misdirect a predator attack: for parallel misdirection, the difference in the centroid of the ellipses between the most and least forwards-confusing butterflies tested was approximately 38% of the average body length (Fig. 2c).

Further work should also incorporate more realistic simulations that consider butterfly wing ventral patterning, as well as a wider range of flight dynamics, viewer angles and distances, and background types. However, we argue that these will probably add nuance to our findings, rather than changing any overall conclusions: for example, these factors may help to explain when a higher-frequency striped pattern evolves over a singular distal stripe.

Our findings call for a reassessment of the mechanistic function of animal patterns while in motion. We trigger exciting avenues for research into the effects of motion confusion in other systems, together with applied uses in the civil and defence sectors, such as improving biologically inspired motion detection for robotics.

## Methods

### Temporally fluid motion measures

To quantify the influence of butterfly wings and their patterns on motion detection, we used a custom-written biologically informed EMD^28,45^, using ImageJ. The model operates using spatiotemporal correlation of pixel intensity values across consecutive frames, quantified with arrays of vertically and horizontally orientated EMDs to measure motion in four cardinal directions (forwards, backwards, left and right)^2^. This version of the EMD model was first validated in ref. ^28^, where it was used to quantify high-speed recordings of broadclub cuttlefish *Sepia latimanus* illusory hunting displays, using the temporal and spatial resolution of green shore crabs, *Carcinus maenas*.

For our model, videos were first processed by spatially and temporally filtering contrasts not visible to the viewer to create ‘fluid motion vision’. Spatial contrast limits were based on those of small passerines (about 6 cycles per degree) observing at 34 cm (smallest) to 193 cm (largest) away, depending on the size of the butterfly. Avian spatial acuity scales with eye and body size^46^, so these viewing parameters should scale allometrically with larger or smaller predators and butterflies. Temporal limits were chosen based on a 100-Hz critical flicker fusion frequency for a passerine within a forest habitat^46,47^. We chose these distances to be as biologically realistic as possible: they are close enough to the target that an avian predator would be likely to initiate their ballistic attack. Closer distances are challenging to model, as they require very high frame rates to avoid motion artefacts (this viewing distance already required simulations at 2,000 fps). Meanwhile, longer viewing distances lead to the stripes no longer being resolvable, and are therefore irrelevant for the current hypotheses and attack ranges.

Filtering was achieved by interpolating frames and pixels using temporal (*σ* = 5) and spatial (*σ* = 8) Gaussian blur. Temporal blurring at *σ* = 5 reduced a 100-Hz flashing stimulus recorded at 1,000 Hz to 1% of its original amplitude^28^. In the absence of both spatial and temporal filtering, EMD outputs would have been strongly influenced by temporal aliasing resulting in ‘wagon wheel’-type motion artefacts that would not occur in nature^35^. The model then outputs motion energy values in each direction (forwards, backwards, left and right) for every pixel and for each consecutive pair of frames.

To quantify motion confusion, the strength of motion in each direction was measured as the mean energy across each frame giving a measure of forwards, backwards, left and right energy. To measure how wing patterns ‘confuse’ motion detection relative to the dominant direction of movement (forwards), we calculated Michelson’s contrast of motion for backwards compared with forwards (forwards confusion), and for sideways compared with forwards (sideways confusion)^29^. Sideways motion was measured using the mean of left energy and right energy.

$$\begin{array}{l}\mathrm{Forwards}\,\mathrm{confusion}=(\mathrm{Backwards}\,\mathrm{energy}-\mathrm{Forwards}\,\mathrm{energy})\\ \,/(\mathrm{Backwards}\,\mathrm{energy}+\mathrm{Forwards}\,\mathrm{energy})\end{array}$$

$$\mathrm{Sideways}\,\mathrm{energy}=(\mathrm{Right}\,\mathrm{energy}+\mathrm{Left}\,\mathrm{energy})/2$$

$$\begin{array}{l}\mathrm{Sideways}\,\mathrm{confusion}=(\mathrm{Sideways}\,\mathrm{energy}-\mathrm{Forwards}\,\mathrm{energy})\\ \,/(\mathrm{Sideways}\,\mathrm{energy}+\mathrm{Forwards}\,\mathrm{energy})\end{array}$$

Forwards confusion gives an estimation of how patterning alters the perceived speed of a moving object, with greater values indicating a greater weighting of motion backwards compared with forwards and a slower speed. For most forwards-moving objects the Michelson’s contrast of backwards to forwards energy should be negative, with biological motion such as flapping wings and running limbs generating cyclical periods of greater backwards compared with forwards energy, for example, when a limb swings backwards. Sideways confusion gives an estimation of how patterning alters the perceived turning rate of an object, with greater values exaggerating or dampening turns. Forwards energy on its own can also mislead observers, with more contrasting objects, such as white, resulting in overestimation as opposed to underestimation of object speed by humans^48,49^. Failure to accurately evaluate the speed or turning rate of an object can result in failure to intercept a target^50^.

It is noted that, for the free-flight videos and the videos presented to human ‘predators’, the direction of the EMD measures did not always align with the heading of the butterfly and was instead globally aligned to the start and end point of the butterfly’s flight path. Meanwhile, for all measurements using the European butterfly simulations and genetic algorithm, the butterfly’s orientation always aligned with the four EMD directions.

### High-speed analysis of butterfly take-off

To pilot whether and how butterfly wing patterns influence early-stage motion detection of their flight, we recorded the take-offs for five Euro-African butterfly species, two of which were recorded for dimorphic sexes, giving a total of seven morphotypes. These were: *Anthocharis cardamines* (males, *N* = 2), *Hypolimnas misippus* (males, *N* = 3 and females, *N* = 3), *Papilio dardanus ochracea* (males, *N* = 3 and females, *N* = 2), *Papilio machaon* (unknown sex, *N* = 2) and *Vanessa atalanta* (unknown sex, *N* = 3). For *H. misippus* and *P. d. ochracea*, the females are mimics of aposematic species of the subfamily Danainae^51,52^. We recorded take-off behaviour as most predator attacks begin on stationary butterflies. All butterflies filmed were adults, with sample sizes determined by availability. As data collected were based on EMD measurements of take-offs, no randomization was required.

Butterfly take-offs were recorded against a green-screen background with a scale bar and Xrite colour checker using a CHRONOS 1.4 (Kron Technologies). The butterflies were typically oriented with their dorsal surface patterning facing the camera. Footage was taken in RAW format at a framerate of 1,057 fps and a resolution of 1,280 × 1,024 pixels. Each species was recorded two or three times (see above for the number of listed flights). Videos were converted from RAW to a DNG stack using the Python function pyraw2dng; these could then be imported into ImageJ and saved in a .tif format. Weka trainable segmentation followed by manual screening and adjustments was used to cluster the video, replacing the background with a dark colour and the butterfly patterning with up to a maximum of four colours^53^. Each video was cropped to include only the area of the flight path and so that the final frame had the entirety of the butterfly visible. Video durations ranged from 174 to 399 frames (0.16 s to 0.38 s)

Once clustered, videos were converted from standard RGB space to avian luminance using blue-tit double-cone quantum catch^54–56^. The background area for each video was replaced with the average luminance for a grassy background (RGB 87, 87, 87). Videos were re-oriented and cropped in length such that the first and last position of the butterfly would follow a linear vertical line upwards (up = forwards) and the butterfly was entirely in frame for its final position. Each video was then passed through the temporally fluid EMD using the methods described above. For each consecutive frame pair, the forwards-confusion and sideways-confusion metrics were calculated using the measured forwards-, backwards-, left- and right-motion energy (Supplementary Video 1). This process was repeated for each video with the butterfly wing patterns replaced with the averaged luminance of the butterfly, 0% reflectance (black) and 100% reflectance (white), giving a total of 4 pattern treatments: natural, averaged, black and white.

European butterflies were caught with a butterfly net in a private garden in France (Mareil-Marly, 78750), housed temporarily in individual plastic pots with airholes and filmed outdoors in a netted enclosure. To film the take-off, the pot was placed upside down in front of a green screen and lifted off once the butterfly had settled on the lid; the butterfly was then allowed to take off naturally. Butterflies were released immediately after recording. African species (*H. misippus* and *P. d. ochracea*), raised from captive-bred pupae, were filmed using a similar set-up; these butterflies were held using soft tweezers, then released for take-off.

### Motion confusion across European butterflies

To assess which features of butterfly wing patterns influence early-stage motion detection of butterflies in flight, we opted to use controlled three-dimensional simulations of European butterflies in flight.

#### Scanning butterfly images

To capture the breadth of European butterfly species, we scanned pages from *Collins Butterfly Guide* as JPEGs using a Konica Minolta bizhub C368 office scanner (Konica House)^57^. As we were interested in patterning more than exact colour values, the accurately scaled illustrations of butterflies within the guide provided an invaluable resource with dorsal images of 397 butterfly species and a total of 757 unique morphotypes (subspecies and sexes).

For each scanned page, individual butterfly dorsal images were cropped and saved in .tif format using a custom-made ImageJ script to semi-automatically select regions of interest for the right forewing and hindwing, as well as a line for the body length. Regions-of-interest selections allowed for later quantification of butterfly wing pattern and shape measures, and for extraction of the forewing, hindwing and body as .pngs for our three-dimensional simulations^45^. Each scan was saved with a scale of 23.583 pixels per millimetre.

#### Three-dimensional rendering in Blender

To simulate flight for the European butterfly morphotypes, we constructed a three-dimensional animated model of a butterfly in Blender 4.0 (Supplementary Video 2). The butterfly consisted of five two-dimensional planes: the body, two forewings (left and right) and two hindwings (left and right). The image of each plane could be replaced with one of the avian double-cone catch converted scans for the butterfly’s body, hindwing and forewing. These planes were rigged with an armature for the forewings and hindwings used to generate flapping flight. The wings were animated symmetrically to match the clap and fling flight of one of the videoed *H. misippus*, with the wings deforming, rolling and yawing distally during the upstroke and downstroke, as opposed to simply pitching upwards and downwards. When animated, the butterfly would fly forwards linearly from its start frame to the end, carrying out two wingbeat cycles.

A custom-written ImageJ + Python script was then used to automatically replace the images used for the butterfly’s body, hindwings and forewings with those of a selected butterfly, as well as to adjust the wingbeat frequency and render the animation^58^. Across Lepidoptera, a smaller wing area corresponds with a higher wingbeat frequency^25,59^. Higher wingbeat frequencies are likely to interact with how patterning influences motion detectors, resulting in increased motion blur. Wing area for each butterfly was converted to the estimated wingbeat frequency in hertz using an equation calculated from the near-linear relationship between butterfly mass, wing area and wingbeat frequency (see Supplementary Information, section 2 for formula calculation)^60^:

$$\begin{array}{c}{\rm{W}}{\rm{i}}{\rm{n}}{\rm{g}}{\rm{b}}{\rm{e}}{\rm{a}}{\rm{t}}\,{\rm{f}}{\rm{r}}{\rm{e}}{\rm{q}}{\rm{u}}{\rm{e}}{\rm{n}}{\rm{c}}{\rm{y}}\\ \,=\,{10}^{(-0.134346\times ((\log (\text{wing area (}{\rm{m}}{{\rm{m}}}^{2}))/\log (10))\times 1.23952-7.843389)+0.53418)}\end{array}$$

The calculated wingbeat frequency was then used to rescale the length of the animation and adjust the start and end frame so that the animation consisted of one wingbeat cycle with ten additional frames lead-in and lead-out for fluid frame interpolation. When rendered, butterflies were filmed from a static bird’s-eye view (camera looking down on the butterfly), flying forwards at a speed proportional to its body length and wingbeat frequency (speed = length × 1.93 × wingbeat frequency). We used only one viewing angle for both tractability and because we did not have access to ventral surface patterns for most of the butterfly species illustrated in *Collins Butterfly Guide*. Animations were rendered as a stack of .pngs at a frame rate of 2,000 frames per second.

Each stack was imported into ImageJ and quantified using the temporally fluid EMD, taking the average forwards, backwards, left and right energy across each frame. These were used to calculate the forwards confusion, sideways confusion and forwards energy for a butterfly in flapping flight. Each butterfly was rendered with three colour treatments: its natural pattern (natural), the averaged grey value of the wing (grey) and in white to account for the effect of the wing shape alone. Natural patterns were used for the random forest analyses, whereas the grey and white renders were used exclusively for linear model comparisons of patterned versus unpatterned butterflies (Supplementary Information, section 2). In addition, each colour treatment was rendered with and without wingbeat animations to determine how gliding influences motion, giving a total of six treatments. This process was carried out for all 757 morphotypes.

#### Butterfly wing features

To assess which features of butterfly wings influenced our EMD model, we quantified the colour, patterning and wing shape of butterflies using image analysis. All measures used were repeated separately for the forewing and the hindwing. Simple measures of colour were obtained by converting the butterfly scans to the CIE 1976 Lab colour space^61^. This colour space is frequently used for the measurement of biological coloration and allows for the quantification of colour across three discrete image channels, L* (achromatic luminance), a* (opponent red–green) and b* (opponent yellow–blue). For each image, we measured the mean and standard deviation (contrast) of a*, b*, and of the Euclidean distance of the a* and b* channels from zero (saturation).

For patterning, we used Gabor filters set to 6 different orientations (0°, 30°, 60°, 90°, 120° and 150°) and 6 octaves, the largest being 1/4× the wavelength of the wing (square root of wing area) to measure the pattern contrast, size and orientations of our blue-tit double-cone catch images^62,63^. The resulting stack of 24 images were then used to create spatial maps for periodicity (how big the pattern is), absolute energy (how contrasting the pattern is), direction encoded as two separate channels VH (0–90 | vertical–horizontal) and OA (135–45 | obtuse–acute), as well as the directionality (anisotropy = √(VH2 + OA2) − average energy; Supplementary Information, section 2). These five maps (periodicity, energy, VH, OA and directionality) allowed us to quantify pattern variation across the wing, not just the global average. For each map, we measured the pixel mean, standard deviation, horizontal gradient (*x*) and vertical gradient (*y*). For instance, a high positive mean VH would indicate a vertical rather than a horizontal (negative) pattern, a positive horizontal gradient for periodicity would indicate that periodicity increases distally from the body, and a high standard deviation of directionality would indicate that directionality varies substantially across the wing.

Lastly, butterfly size and wing shape were measured by calculating the total wing area, the estimated wingbeat frequency and the body length. Forewing and hindwing shape measurements were made by recording the length of the wing from the centre of the wing base to the most distal point on the wing^64^, the breadth of the wing at the centroid of the line for the length of the wing, the rectangular length and width of the wing, the area of the wing relative to the body length (area/body length), the aspect ratio of the wing (length/breadth), the circularity of the wing (4π(area/perimeter^2^))^45^, and the roughness of the wing (the convex area/the actual area). Altogether, this gave us 73 measures for wing features.

### In silico butterfly pattern evolution

The diversity of butterfly wing patterns is a product of unique genetic and developmental pathways, some of which have been accurately mapped^4,65,66^. These could either limit the adaptive potential of their patterning for motion confusion or could create patterns that fit our motion-confusion hypothesis by coincidence. To validate whether our motion-confusion metrics corresponded with actual butterfly wing evolution, we opted to simulate evolution and selection for motion confusion with genetic algorithms^67,68^. Genetic algorithms provide a powerful tool for exploring the fitness landscape of animal phenotypes, and have seen increasing use within visual ecology research, notably for animal camouflage research^69–71^. These algorithms mimic evolution by natural selection to efficiently explore vast multidimensional solution spaces, such as those of animal patterns.

For the algorithm, we modified the existing animal pattern evolution framework of the CamoEvo Toolbox, which utilizes decimal encoding of reaction-diffusion patterns and subsequent image modifications to produce artificial animal patterns, with mutation operators tailored for the evolution of patterning^71^. This framework was altered to produce images of artificial butterflies where the parameters for pattern shape were separate for the forewing, hindwing and body of the butterfly, allowing for them to evolve independently from one another. As with the real European butterflies, the butterfly images were then transferred to Blender and animated with our three-dimensional butterfly rig.

To account for any influences wing shape might have had on evolution, we selected three distinct butterfly wing shapes from three of the butterfly families: the proportionally small wings of a ‘Skipper’ Hesperiidae (*Pyrgus andromedae*), the distinct tailed wings of a ‘Swallowtail’ Papilionidae (*Papilio machaon*) and the more classically shaped wings of a Nymphalid butterfly (*Pseudochazara anthelea*)^72^. For each wing shape, we assigned populations of *N* = 24 butterflies to one of three different measures of ‘fitness’ representing different selection pressures: forwards confusion, sideways confusion and forwards energy. For each generation, the top-8 individuals (that is, those with the highest motion-confusion and energy metrics) would ‘survive’, whereas the remaining 16 ‘died’ and were replaced by mutant recombinant offspring of the survivors in 2 rounds of random pairings. In addition, we evolved butterflies under random selection, where the rank order was randomly generated each generation: this was done to ensure that butterflies did not evolve patterns simply because of drift. Each population evolved for 20 generations, starting with a population of randomly generated individuals, and with 10 repeats for each fitness metric per wing shape, giving a total of 120 populations (3 wing shapes, 4 selection pressures and 10 repeats).

Butterflies were expected to become ‘fitter’ with each generation, and to select for similar features to those identified to predict motion confusion in real butterflies. As high-spatial-frequency patterns and speckles were not under selection owing to spatial filtering from our fluid EMD model, both the patterns of real butterflies and the genetic-algorithm-generated in silico butterflies underwent Gaussian spatial filtering (*σ* = 8) before measurement. For both sets of butterflies, we used the same wing pattern metrics as the European butterfly analysis, excluding measures of wing shape or CIE Lab colour. We chose to use population sizes of 24 individuals and 20 generations based on previous applications^63,71^ and as we wished to determine the most immediately selected characteristics and to preserve diversity for motion confusion, rather than determine the peak optimum phenotypes.

### Behavioural validation of measures

To test whether or not our motion-confusion metrics could predict changes in predator-attack behaviour, we constructed a high-framerate (240 Hz) touch-screen butterfly capture game using the three-dimensional butterfly rig used for the in silico evolution and analysis of scans from *Collin’s Guide to Butterflies*. This involved the following: subselecting butterflies that were representative of different levels of motion confusion; rendering the butterflies onto 10 different flight paths to create animations for a touch-screen game; and recruiting 100 volunteers to collect data on how motion-confusion measures before the player touching the screen influenced where they clicked relative to the butterfly.

For the game, we sub-selected five butterfly phenotypes from the range of *Collins Guide to Butterflies*. These butterflies were selected from across the motion-confusion gradient for forwards and sideways confusion: *Brintesia circe* (male), *Papilio alexanor* (female; it is noted that this species has the highest sideways confusion), *Pyrgus sidae* (male), *Gonepteryx cleobule* (male) and *Lycaena tityrus* (female). See Supplementary Information, section 4 for full table.

#### Butterfly animation and flight behaviour

As with the Collins and genetic-algorithm simulations of butterflies, we used Blender to create artificial renderings of butterflies. Butterflies were re-scaled so that they had the same wing area. Butterflies had a mean body length of 2.42 cm when rendered on the screen. Wingbeat frequencies were adjusted based on body size and wing area, as with our previous simulations. However, unlike the previous simulations, butterflies were animated with an average flight speed of approximatley 40 cm s^−1^ for a period of 1.458 s, with multiple wingbeat cycles per flight and sinusoidal flight paths, to simulate natural flight behaviour and make them more difficult to capture. Butterflies initiated their flight from the bottom-left corner of a 1,280 × 720 pixel background image, which was the same colour as the previously used backgrounds (RGB 87, 87, 87) and would then fly off the screen either from near the bottom-right or top-right corners. The start position of the butterflies was offset from the centre point in a 50° arc (±25°) centred on 45° from the left corner of the background (Supplementary Fig. 25).

As the flight paths were encoded using waves and differences in starting orientation, we were able to create saveable flight paths that could be efficiently stored and used across different butterfly phenotypes. For more details of how the sinusoidal flight was encoded, see Supplementary Information, section 4. We randomly generated 50 flight paths and then sub-selected 5 paths where the endpoint was in the top-right corner and 5 where the endpoint was in the bottom-left corner. The five selected paths for each end point were uniformly selected from low to high levels of linearity (inverse of the level of displacement from the straight-line path from start to end). The settings of each of these paths were saved as .json files, which could be reloaded using Blender and Python, allowing the butterfly phenotypes to be mapped to the same motion paths. See Supplementary Fig. 26 for illustrations of each flight path.

#### EMD measurements and hitboxes

Butterfly videos were rendered at a frame rate of 1,000 fps and then underwent temporal motion smoothing with the same Gaussian blur levels used for the EMD analyses. As birds see faster than humans, and pilot experiments found that human participants struggled to catch butterflies moving faster than 50 cm s^−1^, we opted to create targets that flew across the screen at 40 cm s^−1^, but had the equivalent wingbeat frequency and blur to a butterfly travelling at double the speed 80 cm s^−1^, which was in the range of speeds for our free-flying butterfly take-offs. This was achieved by resampling the number of frames and then dividing the wingbeat frequency by two for the Blender render before applying the blurring method (Supplementary Fig. 27).

After applying temporal smoothing, we downsampled the number of frames to match the frame rate of the monitor, 240 fps, for the game (Supplementary Information, section 4(v)) For each flight path and each phenotype, a second video was rendered using a labelled hitbox created for the butterfly’s image. This hitbox was colour-coded such that the red channel could be used to indicate whether a butterfly was hit (butterfly = 255 R), and the green channel could indicate which region of the butterfly was hit (body = 10 G, forewing = 200 G, hindwing = 255 G or tail = 30 G).

For each butterfly and for each path, we measured EMD using the same method of alignment as the butterflies filmed in free flight. The images were re-oriented so that the start and end points were in a straight vertical line upwards. The forwards (up), backwards (down), left and right EMD were then used to calculate the confusion measures (forwards and sideways confusion) for the butterflies. This gave us the EMD and confusion measures for every frame. As confusion varied throughout the flight for each butterfly owing to differences in wingbeat cycle and heading (Supplementary Fig. 28), we created a measure for the mean EMD values and confusion values starting from 400 ms (96 frames) before each frame. However, it is worth noting that changes in the orientation of the butterfly will have resulted in noise in the motion-confusion measures, as they were not always aligned with the direction of movement. For instance, forwards confusion was on average higher for *P. alexanor* than *B. circe*, probably owing to movements perpendicular to the start and end heading.

#### Butterfly game

For the experiment, we recruited 100 anonymous volunteer players from across the University of Exeter’s Penryn Campus (Penryn, Cornwall, UK, TR10 9FE) to play a touch-screen-based butterfly-catching game. Participants were required to be aged between 18 and 70 years old, with normal or corrected to normal vision (that is, wear glasses if needed). The sex and age of the participants were not recorded.

The game was run using an ASUS Aspire Nitro V15 laptop with an in-built NVIDIA GEFORCE RTX graphics card, allowing us to display the game with a secondary screen for our experiment, a graphics-processing-unit-powered 240-Hz ASUS XG32UCWMG gaming monitor. The screen for the experiment was 71 cm wide and 40 cm high, with a maximum resolution of 1,920 × 1,080 when in enhanced frame rate mode. Although the uppermost frame rate of the screen was 480 Hz, we selected a vertical refresh rate of 240 Hz for increased stability.

To convert the screen into a touch screen, we used a greentouch infrared frame, placed over the monitor and with a thin (2 mm thick) acrylic sheet cut to fit behind it to protect the screen. Infrared frames allow objects, that is, a participant’s finger, that penetrate the frame to be registered as clicks (Supplementary Fig. 29). Infrared frames are advantageous compared with alternative touch device methods owing to their high response rate >240 Hz and their ability to be paired with almost any screen. This method for high-frame-rate touch screens was based on previous studies^73,74^. Players were seated on a chair positioned about 50 cm away from the screen and were asked to adjust the height of the screen and to adjust themselves to where they could comfortably reach all four corners of the screen without fully extending their arm, and so that their head faced the centre point of the screen. The mean body length of our butterflies was 2.42 cm, giving an angular width of approximately 2.80° and an angular speed of approximately 58.99° s^−1^.

To create the game, we used MATLAB R2025 along with the Psychtoolbox-3 and a suite of custom functions for initializing the screen and Psychtoolbox and for registering the touch output as clicks, dubbed PECK as they were initially developed for experiments using birds^73^. As PECK has not yet been released, a stripped version of it including only functions necessary for the experiment has been provided within both our Github and Figshare repositories^75^.

Each player undertook 30 trials rendered at 240 Hz with 6 replicates of each butterfly phenotype. The trials used random flight paths, and each path could be flipped on the *x* and/or *y* axis. This gave a total of 3,000 trials across 100 participants. Before each trial, the player was first instructed to click on a white circle positioned in the same corner from which the butterfly would start. This meant that the player’s finger always started behind the butterfly. The butterfly would then immediately appear, and the player would have to ‘attack’ it using a single finger. Once the player touched the screen, the butterfly would pause, and text would appear revealing whether the player had successfully hit or missed the butterfly. If the butterfly’s hitbox was within a 30-pixel radius of the click, it was considered a hit. This was to account for the size of the participant’s finger.

Before the experiment commenced, the player was given a series of instructions informing them how to play the game and asking them to use only their dominant hand and one finger when playing the game. Players also did a short tutorial to ensure that they understood the instructions and to ensure that they could catch the butterflies at the speed they travelled in the experiment. This tutorial tasked the player to try and click on butterflies of 3 increasing speeds, 0.5×, 0.75× and 1.0× the speed of the butterfly used in the experiment. To progress to the next speed, the player needed to successfully catch two butterflies at that speed. Players, on average, took 14 ± 6 (mean ± s.d.) trials to complete the tutorial. In addition, during the tutorial, when the player clicked the screen, they were also given information about whether their click was to the front or back and left or right of the butterfly. These trials were not included in the count of 30 experimental trials. During experimental trials, the player was no longer provided with any additional information or instruction. When the screen was clicked, they were only informed on whether they hit the butterfly or not, rather than where they clicked relative to the butterfly.

#### Data collection

For each experimental trial, we recorded the unique ID assigned to the player, the flight path and butterfly phenotype used, the order number of the trial, whether the path was flipped on the *x* and/or *y* axis, the coordinates and frame of the touch/click if there was one and the RGB values of the nearest pixel of the hitbox to the click. By using the heading of the butterfly, we were able to calculate the angle of the click relative to the butterfly and the distance of the click on different vectors, the distance parallel to the butterfly’s (**y** vector) heading and the distance perpendicular (**x** vector). As click distances were defined relative to the butterfly headings, the **y** vector was allowed to be positive or negative, with negative values indicating clicks behind the centre of the butterfly and positive values being in front.

### Statistical analyses

All statistical analyses were carried out using R version 4.3.3^76^.

To determine how wing pattern influenced the motion-confusion metrics for real butterfly take-offs in free flight, we used linear mixed models (LMMs), with the ‘lme4’ package^77^. For the analysis, the motion-confusion metrics (forwards confusion, sideways confusion and forwards energy) were considered as dependent variables in separate models, with wing pattern type (natural, averaged, black and white) as a factorial predictor variable. The ID number for each unique flight and the species were used as random effects. To compare differences between the factor levels, we used emmeans Tukey post hoc comparison tests^78^.

Given the vast array of wing features measured, we opted for a random forest model to evaluate the predictive value for each of the measures for each of our motion-confusion measures^33^. Random forest provides a non-parametric ensemble learning method, using multiple constructed decision trees trained with random subsets of the data and the predictors. Predictions are then aggregated across the trees helping to prevent overfitting. For each of our metrics, random forest models were trained with 1,500 trees with 4 predictors per split. To evaluate model performance, we used *R*^2^ to estimate the proportion of explained variance.

Recursive feature elimination was used to improve model interpretability by reducing redundant predictors. Predictors were systematically ranked based on their weight and model performance was evaluated for subsets from one to the total number of variables.* R*^2^ values of the complete model and the reduced models were compared to ensure that the predictive accuracy was retained. To evaluate which features of the reduced model were the most important, we used Shapley additive explanations (SHAP) as a measure of feature importance^79^. Greater absolute SHAP indicates a greater importance, with positive and negative values corresponding with a positive or negative correlation, respectively.

Random forest models were performed using ‘randomForest’ and ‘caret’ packages^33,80^. As many of the wing features are potentially linked to phylogeny, we incorporated the complete European butterfly phylogeny of Wiemers et al. into the model^72^. Cophenetic distances were extracted from the phylogeny and converted into three phylogenetic principal components with principal coordinate analysis using the ‘phytools’ package^81^. Similar to how principal component analysis can reduce multiple numeric variables into singular components, principal coordinate analysis allows an individual’s position within the phylogeny to be redefined as a set of multidimensional coordinates. Our three phylogenetic components were included alongside the wing parameters as predictor variables within our model. Circular coordinates for the butterfly phylogeny were extracted with the ‘ape’ package^82^.

For the in silico butterflies, LMMs were used to validate whether butterflies significantly improved in fitness between the first and last generation for each of our selection variables (forwards confusion, sideways confusion and forwards energy) for our evolution treatments. Random forest models were again generated for the motion-confusion metrics using our reduced set of spatially filtered wing pattern variables. These models were created using the measured phenotypes from the first and last generation of the entirety of our in silico-generated dataset, rather than for butterflies under one selection pressure, using the initial randomly generated butterflies to provide a wider range of variation. Lastly, to determine whether or not butterflies under a particular selection pressure evolved to be more or less like the 757 natural butterfly morphotypes scanned from Collins, we generated principal components for the same wing pattern metrics used for our real butterflies, barring measures of colour as the artificial butterflies were rendered only in the luminance channel, (PC1 and PC2) and used pairwise comparison to calculate the Euclidean difference of principal components between every butterfly, both real and in silico, from every natural butterfly.

Instances where real butterflies of the same morphotype were compared were removed as their distance would equal 0. Butterflies were divided into 6 groups: natural, forwards-confusion selected (gen = 20, top 1 per population), sideways-confusion selected (gen = 20, top 1 per population), forwards-energy selected (gen = 20, top 1 per population), random selection (gen = 20, top 1 per population) and unevolved (gen = 0, all individuals). We then used a linear model to test which group generated the lowest difference from real butterflies and whether the pairwise distance levels were significantly different from one another with Tukey post hoc comparisons. We hypothesized that the difference should be lower for those selected for motion confusion than for those that were randomly generated at the start.

To test whether or not our motion-confusion measures influenced how the butterflies were clicked relative to the butterflies for our behavioural validation, we used an LMM, with the vector distance as the response variable and the axis (*x* or *y*) and the predicted motion-confusion measures, forwards confusion, sideways confusion and the forwards energy, of the butterfly as the predictor variables, with the player ID, the trial order and the path ID as random effects, in addition to frame rate as during some trials frame rate dropped to about 220 Hz. For the model, we included interactions between the axis and each confusion measure. Our expectations were that forwards confusion should influence the click distance parallel to the butterfly (*y*) and that sideways confusion should influence the click distance perpendicular (*x*).

In addition to the linear model, we used two-dimensional kernel density estimation with the Mass package for our vectors to determine how the butterfly phenotypes influenced the shape of the distribution of clicks^68^. We extracted the grid for the top-25% densest regions of clicks for each butterfly and used ellipse statistics (centroid coordinates, angle, aspect ratio, major and minor width) to describe the shape of where players tended to click. We also used a binomial generalized LMM with the same random effects as the linear model to test whether our EMD measures influenced the likelihood of clicking forewing or hindwing. Clicks to the body and misses were excluded from this analysis.

For each model, continuous predictor and response variables were re-scaled to a mean of 0 and a standard deviation of 1.

### Ethics statement

Use of butterflies for flight recordings was approved by the University of Exeter ethics committee (application number eCORN000385). For the behavioural validation game, volunteers were recruited via email and by signs, with ethical approval from the University of Exeter ethics committee (REF: 9474868) and in line with the Declaration of Helsinki. No identifying data were collected as part of the experiment. All volunteers were required to read through the information sheet and sign a copy of the participant consent form. Copies of these forms are available within our data repository^75^.

### Reporting summary

Further information on research design is available in the Nature Portfolio Reporting Summary linked to this article.

## Online content

Any methods, additional references, Nature Portfolio reporting summaries, source data, extended data, supplementary information, acknowledgements, peer review information; details of author contributions and competing interests; and statements of data and code availability are available at https://doi.org/10.1038/s41586-026-11062-w.

## Supplementary information

Supplementary Information This file contains real butterfly take-off recordings, motion confusion across European butterflies, butterfly artificial evolution with genetic algorithms, behavioural validation of motion-confusion measures for butterflies, and references.

Reporting Summary

Peer Review File

Supplementary Video 1 Butterfly high-speed recording: this video provides a visual summary for the high-speed video recording methods used for live butterflies and their measurement with an elementary motion detector.

Supplementary Video 2 Butterfly three-dimensional rendering: this video provides a visual summary for how butterfly images from *Collins Guide to Butterflies* were rendered in three dimensions with Blender 4.0 for measurement with an elementary motion detector. Images of butterflies were used from the *Collins Butterfly Guide* with permission from Richard Lewington.

Supplementary Video 3 Butterfly genetic algorithm: this video provides a visual summary for how digital butterflies were generated and then ‘evolved’ using a genetic algorithm by rendering them with our three-dimensional model for Blender 4.0 and measuring them with an elementary motion detector to rank patterns for selection.

Supplementary Video 4 Behavioural experiments: this video provides a visual summary for how our digital Blender 4.0 animations of butterflies were used to create a butterfly-catching touch-screen game.

## Data Availability

The time-calibrated butterfly phylogenetic data we used are open access and accessible within ref. ^72^ (https://doi.org/10.3897/zookeys.938.50878). We provide a copy of their Newick tree within both our Github (https://github.com/GeorgeHancock471/Butterfly-Motion-Illusions) and figshare repositories^75^. We also provide our elementary motion detector data for our live butterflies and blender-rendered butterflies, as well as our pattern measures for the butterflies scanned from *Collins Butterfly Guide* and generated by our genetic algorithm.

## Code availability

All R code used for statistical analysis is provided within our Github (https://github.com/GeorgeHancock471/Butterfly-Motion-Illusions) and figshare (https://doi.org/10.6084/m9.figshare.31626616) repositories^75^, and divided by section: Take-off analyses, Collins analyses, and genetic-algorithm analysis. A copy of the ImageJ plugins used is also provided and includes within them a copy of the butterfly Blender model and EMD measurement scripts. The MATLAB code for the butterfly game is also provided.
