# Supplementary material for: Butterfly wing patterns in flight create powerful illusory motion cues
Source: Nature. 2026 Sep 30;658(8137):958–62. doi: 10.1038/s41586-026-11062-w (PMC13686948; doi:10.1038/s41586-026-11062-w)
Supplement: Supplementary file 1 [file 41586_2026_11062_MOESM2_ESM.pdf]

Corresponding author(s): Jolyon Troscianko
Last updated by author(s): Aug 11, 2026

Reporting Summary

Nature Portfolio wishes to improve the reproducibility of the work that we publish. This form provides structure for consistency and transparency in reporting. For further information on Nature Portfolio policies, see our Editorial Policies and the Editorial Policy Checklist.

Statistics

For all statistical analyses, confirm that the following items are present in the figure legend, table legend, main text, or Methods section.

n/a	Confirmed	
☐	☒	The exact sample size (*n*) for each experimental group/condition, given as a discrete number and unit of measurement
☐	☒	A statement on whether measurements were taken from distinct samples or whether the same sample was measured repeatedly
☐	☒	The statistical test(s) used AND whether they are one- or two-sided<br>*Only common tests should be described solely by name; describe more complex techniques in the Methods section.*
☐	☒	A description of all covariates tested
☐	☒	A description of any assumptions or corrections, such as tests of normality and adjustment for multiple comparisons
☐	☒	A full description of the statistical parameters including central tendency (e.g. means) or other basic estimates (e.g. regression coefficient) AND variation (e.g. standard deviation) or associated estimates of uncertainty (e.g. confidence intervals)
☐	☒	For null hypothesis testing, the test statistic (e.g. *F*, *t*, *r*) with confidence intervals, effect sizes, degrees of freedom and *P* value noted<br>*Give P values as exact values whenever suitable.*
☐	☒	For Bayesian analysis, information on the choice of priors and Markov chain Monte Carlo settings
☐	☒	For hierarchical and complex designs, identification of the appropriate level for tests and full reporting of outcomes
☐	☒	Estimates of effect sizes (e.g. Cohen's *d*, Pearson's *r*), indicating how they were calculated

Our web collection on statistics for biologists contains articles on many of the points above.

Software and code

Policy information about availability of computer code

Data collection	Commercial:<br>Matlab 2025R (mathworks.com/products/matlab.html), Psychtoolbox Matlab 2025R (http://psychtoolbox.org/), Windows OS operating system<br>Open Source / Free:<br>ImageJ V1.54 (https://imagej.net/ij/), Blender 4.0 (https://www.blender.org/), GIMP 3.2.4 (https://www.gimp.org/), Python (https://www.python.org/)<br>Custom:<br>All custom scripts for Blender, Python, and ImageJ have been made available as a part of our GitHub (https://github.com/GeorgeHancock471/Butterfly-Motion-Illusions). Additional instructions for using our custom EMD model are provided in our README file.
Data analysis	All statistical analyses were carried out in R version 4.3.3. All R code use including the list of libraries necessary has been made available through our GitHub (https://github.com/GeorgeHancock471/Butterfly-Motion-Illusions).

For manuscripts utilizing custom algorithms or software that are central to the research but not yet described in published literature, software must be made available to editors and reviewers. We strongly encourage code deposition in a community repository (e.g. GitHub). See the Nature Portfolio guidelines for submitting code & software for further information.

Data

Policy information about availability of data

All manuscripts must include a data availability statement. This statement should provide the following information, where applicable:

- Accession codes, unique identifiers, or web links for publicly available datasets
- A description of any restrictions on data availability
- For clinical datasets or third party data, please ensure that the statement adheres to our policy

Time-calibrated butterfly phylogenetic data we used is open-access and is accessible within Wiemers et al., 2020 publication https://doi.org/10.3897/zookeys.938.50878. We provide a copy of their Newick tree and the R code we created for the acquisition of circular coordinates used in Figure 2 within our GitHub repository (https://github.com/GeorgeHancock471/Butterfly-Motion-Illusions). We also provide our elementary motion detector data for our live butterflies and blender-rendered butterflies, as well as our pattern measures for the butterflies scanned from Collins Butterfly Guide and generated by our genetic algorithm

Research involving human participants, their data, or biological material

Policy information about studies with human participants or human data. See also policy information about sex, gender (identity/presentation), and sexual orientation and race, ethnicity and racism.

Reporting on sex and gender	No data on sex or gender was recorded, participants were anonymous.
Reporting on race, ethnicity, or other socially relevant groupings	No data on race or ethnicity was recorded, participants were anonymous.
Population characteristics	No population characteristic data was recorded, participants were anonymous.
Recruitment	For our experiment, we recruited 100 anonymous volunteer players from across the University of Exeter's Penryn Campus (Penryn, Cornwall, UK, TR10 9FE) to play a touch-screen-based butterfly-catching game. Volunteers were recruited via email and by signs, with ethical approval from the University of Exeter ethics committee (REF: 9474868) and in line with the Declaration of Helsinki. While there was some potential for self-selection bias from the method of recruitment, it was required as per University of Exeter's recruitment policies and was not expected to influence our hypotheses which are based on low-level motion detection and visual processing. No identifying data was collected as part of our experiment. All volunteers were required to sign a copy of our participant consent form as well as to read through our information sheet. Copies of these forms are available within our supplementary materials.
Ethics oversight	University of Exeter ethics committee (REF: 9474868)

Note that full information on the approval of the study protocol must also be provided in the manuscript.

Field-specific reporting

Please select the one below that is the best fit for your research. If you are not sure, read the appropriate sections before making your selection.

☒ Life sciences ☐ Behavioural & social sciences ☐ Ecological, evolutionary & environmental sciences

For a reference copy of the document with all sections, see nature.com/documents/nr-reporting-summary-flat.pdf

Life sciences study design

All studies must disclose on these points even when the disclosure is negative.

Sample size	We conducted four key experiments, each utilizing their own data sets.<br>(1) Elementary motion detector (EMD) measurement of butterfly take-off<br>We recorded the take offs for five Euro-African butterfly species, two of which were recorded for dimorphic sexes, giving a total of 7 morphotypes. These were: Anthocaris cardamines (m, N=2), Hypolimnas misippus (m, N=3 & f, N=3), Papilio dardanus ochracea (m, N=3 & f, N=2), Papilio machaon (n/a, N=2), and Vanessa atalanta (n/a, N=3). For H.misippus and P. d. ochracea the females are mimics of aposematic species of the subfamily Danainae. m=male, and f=female.<br>Butterflies were filmed with a 1000 fps camera with flights ranging from 174 to 399 frames (0.16 to 0.38 seconds). This gave a total of N=2391 frames. Butterfly EMD measures were then compared between patterned, averaged grey, white, and black for all N=2391 frames.<br>The sample size of butterflies was determined by availability and time.<br>(2) Elementary motion detector (EMD) measurement of 3D modelled butterflies<br>We scanned Collin's Guide to Butterflies to extract N=757 unique phenotypes of butterflies to render on our 3D Blender of a butterfly and then our EMD model. Results were averaged across frames each butterfly giving a total sample size of N=757. With a sample size of N = 1 for each phenotype as only a single linear flight and one wing beat cycle was used.<br>Sample size was determined by the number of dorsal wing phenotypes present within Collin's Guide to Butterflies.

(3) Artificially evolved butterflies with a genetic algorithm
Using genetic algorithms, we artificially selected for butterfly phenotypes with increased motion confusion measures for three different measures, forwards confusion, sideways confusion, and forwards energy, as well as randomly. For each measure we evolved 30 populations of butterflies (3x wing shapes and 10x populations per wing shape). Populations evolved for 20 generations with 24 butterflies per population.This gave a total of 57,600 butterflies. For comparisons of butterfly pattern measures between our artificially evolved butterflies and those from our scans of Collin's guide to butterflies we sub selected the fittest individual from the last generation of each group (N = 30 for each measure including random), while for the unevolved we used all 2,160 phenotypes and for Collin's we used all 757 phenotypes. We then calculated PC1 and PC2 Euclidean distances of phenotype measures for the artificial butterflies (unevolved, forwards confusion, sideways confusion, forwards energy, and random) from the natural butterflies in Collin's Guide to Butterflies.
(4) Butterfly game
We recruited 100 participants from the University of Exeter to take part in our catch the butterfly game. Each participant was tasked with catching 30 butterflies (5 phenotypes each with 6 replicates). This gave a total of 3000 trials, instances where a participant failed to click the screen were removed reducing the total to 2985. (L.t N = 597, G.a N = 599, P.s N=598, P.a N = 592, B.c N =599). We used pilot data from 20 participants to build simple simulations for whether a sample size of 100 was sufficient to detect significant differences within our dataset and we found a 90% power for detecting an estimated marginal mean difference in number of hits of 0.45. Butterflies were arranged in randomly ordered blocks of 6 meaning that a butterfly could not occur more than twice in a row.

Data exclusions

(1) No data excluded

(2) No data excluded

(3) Best individual from each final population was sub selected to prevent overweighting the principal component space with the artificial pattern measures rather than the natural butterflies. Decision was made as a prior.

(4) Instances where no click was recorded were excluded due to the lack of click coordinate data.

Replication

(1) Multiple flights were taken for each species which act as replicates, but no replication of the experiment was carried out.

(2) No additional replication was needed as this study entirely relied on 3D simulations where any replication would produce exactly the same result.

(3) 10 replicates were performed for each wing shape and selection pressure. Differences between the replicates were accounted for in the statistical analyses. But the experiment itself was not replicated.

(4) 600 replicates were carried out for each treatment group, across the 100 participants. But no replication of the actual experiment was carried out.

Randomization

(1) No randomization, butterflies were filmed opportunistically in order of capture.

(2) No randomization required, because this was simulation data and no humans or animal observers were involved.

(3) No randomization required because this was simulation data and no humans or animal observers were involved.

(4) Participants were assigned a pre-generated random order sequence of trials from a list of 100. Participants undertook these sequences in order of arrival for the experiment.

Blinding

(1) No blinding was required for this study, all butterfly footage was analyzed automatically using elementary motion detectors.

(2) No blinding was required for this study as it was a simulation.

(3) No blinding was required for this study as it was a simulation.

(4) All data was collected automatically by the touch screen game.

Reporting for specific materials, systems and methods

We require information from authors about some types of materials, experimental systems and methods used in many studies. Here, indicate whether each material, system or method listed is relevant to your study. If you are not sure if a list item applies to your research, read the appropriate section before selecting a response.

Materials & experimental systems

n/a	Involved in the study
☒	☐ Antibodies
☒	☐ Eukaryotic cell lines
☒	☐ Palaeontology and archaeology
☐	☐ Animals and other organisms
☒	☐ Clinical data
☒	☐ Dual use research of concern
☒	☐ Plants

Methods

n/a	Involved in the study
☒	☐ ChIP-seq
☒	☐ Flow cytometry
☒	☐ MRI-based neuroimaging

Animals and other research organisms

Policy information about studies involving animals; ARRIVE guidelines recommended for reporting animal research, and Sex and Gender in Research

Laboratory animals

Species:
1. Hypolimnas misippu, 3x male sex and 3x female sex, adult
2. Papilio dardanus ochracea, 3x male sex and 2x female sex, adult
Origin:
No laboratory animals were used in the study.
Species were captive reared from larvae as a part of a private butterfly collection as opposed to from laboratory lines. The exact age of the butterflies could not be determined. Butterflies that were heavily worn or damaged were avoided.

Wild animals

Species:
1. Anthocaris cardamines, 2x male sex, adult
2. Vanessa atalanta, 2x N/A sex, adult
3. Papilio machaon, 2x N/A sex, adult
Capture & Housing:
Animals were captured by using an etymological net in aprivate garden in France (Mareil-Marly, 78750) and housed temporarily using pots with air holes. Butterflies were released at their site of capture and on the same day as capture after being filmed. The exact age of the butterflies could not be determined. Butterflies that were heavily worn or damaged were avoided.

Reporting on sex

Only species which were sexually dimorphic in patterning were sexed (Anthocaris cardamines, Papilio dardanus ochracea, and Hypolimnas misippu). Sex was not included as a part of the analysis of filmed live butterflies.

Field-collected samples

No field collected samples were used in the study.

Ethics oversight

Use of butterflies for flight recordings was approved by the University of Exeter ethics committee (application number eCORN000385).

Note that full information on the approval of the study protocol must also be provided in the manuscript.

Plants

Seed stocks

Report on the source of all seed stocks or other plant material used. If applicable, state the seed stock centre and catalogue number. If plant specimens were collected from the field, describe the collection location, date and sampling procedures.

Novel plant genotypes

Describe the methods by which all novel plant genotypes were produced. This includes those generated by transgenic approaches, gene editing, chemical/radiation-based mutagenesis and hybridization. For transgenic lines, describe the transformation method, the number of independent lines analyzed and the generation upon which experiments were performed. For gene-edited lines, describe the editor used, the endogenous sequence targeted for editing, the targeting guide RNA sequence (if applicable) and how the editor was applied.

Authentication

Describe any authentication procedures for each seed stock used or novel genotype generated. Describe any experiments used to assess the effect of a mutation and, where applicable, how potential secondary effects (e.g. second site T-DNA insertions, mosiacism, off-target gene editing) were examined.
