# Supplementary material for: Butterfly wing patterns in flight create powerful illusory motion cues
Source: Nature. 2026 Sep 30;658(8137):958–62. doi: 10.1038/s41586-026-11062-w (PMC13686948; doi:10.1038/s41586-026-11062-w)
Supplement: Supplementary file 2 [file 41586_2026_11062_MOESM3_ESM.pdf]

nature portfolio

Peer Review File

Butterfly wing patterns in flight create powerful illusory motion cues

Corresponding Author: Dr Jolyon Troscianko

Any redactions in this file are there to maintain patient confidentiality, the confidentiality of unpublished data, or to remove third-party material.

This file contains all reviewer reports in order by version, followed by all author rebuttals in order by version.

Version 0:

Reviewer comments:

Referee #1

(Remarks to the Author)
"Butterfly wing patterns create powerful illusory motion cues"

The manuscript explores how butterfly wing patterns affect the accuracy of motion cues. The authors make use of an "Elementary Motion Detector" visual model that quantifies directional motion "energy" generated by pixel comparisons between consecutive frames of a video. Any directional energy values which indicate motion in directions other than the true direction of motion are considered to contribute to motion confusion, which the authors argue has the potential to mislead predators into mis-targeting their attacks on moving prey.

The authors apply their model to three different data sets: videos of real butterfly take-off motion, simulated butterfly flight of a wide range of different species' patterns, and artificially generated patterns that undergo simulated selection for motion confusion. They find that many natural butterfly patterns have higher values for motion confusion than plain patterns, and that artificial patterns selected for motion confusion increase their similarity to natural patterns.

1. The manuscript is likely to be of wide interest, bringing a clear strategy for addressing what has been an under-explored aspect of animal colour patterns. This comes in the context of huge research interest in butterflies as a model for animal colour patterning, and with the possibility of applying the technique to a range of other taxa. The key question in my mind while reading the manuscript is: does "motion confusion" as measured here actually confuse predators? I appreciate that this is a slightly separate question to the one the authors have asked, and they do acknowledge the need for behavioural experiments (line 188). Although references are made to the EMD model being "well validated" (lines 184, 223), as far as I can see, only two of the referenced studies (36 and 37) compare it against receiver behaviour, both of which involve humans responding to computer-generated cues. How confident can we be that predators targeting flying prey use analogous methods for judging motion? In my opinion, this has the potential to be a classic and extremely well-cited paper if future behavioural experiments back up the model, but I'm not sure that is guaranteed.

2. The authors have generally been careful in their language to avoid over-stretching the conclusions (e.g. line 163 "consistent with selection for motion confusion", line 166 "how motion confusion could operate"). On the other hand, this might be pedantic, but can we say that the motion illusion is "powerful" (line 1) when we don't know its effect? Can we say that motion confusion is an evolutionary "strategy" (line 12) when we don't know if it could be simply a biproduct of selection for warning colouration? For consideration for future work: are there differences between the patterns we would predict under motion confusion vs aposematism that could be used to separate the two strategies?

3. The manuscript is innovative in its approach, using sophisticated image analysis to generate motion confusion measures. The use of animation enables the authors to expand their taxonomic coverage from the smaller numbers of species used in live video capture, to encompass a comprehensive range of European species. This is a nice way to overcome the practical limitations of data collection, while keeping measurements grounded in reality. The methods generally appear to be very well implemented, with care taken to account for the visual systems of realistic signal receivers (passerine birds, which are justifiably assumed to be key selective agents here). However, all modelling is based on images filtered to a certain level of

visual acuity (line 229). Although this value has a biological basis, it is an angle, and so the amount of spatial information visible depends heavily on the viewing distance. On what distance were the results based, and how robust are the results to changes in that value?

4. Animation of butterflies was based on the flight pattern of a single species (line 327). The authors adjust for speed and wingbeat frequency but the "shape" (I can't think of a better word) of wing motion is constant across all animated species. I would imagine that there might be a strong interaction between wing morphology and flight patterns. How much do species vary in their flight patterns, and how much effect does the flight pattern have on the measures of confusion? Would it be valuable to carry out a test of robustness to changing the way in which the motion is animated?

5. There are some interesting effects of background matching that are evident in the supplementary information (lines 138-141 and Supp Fig 8). It seems that background matching, and hence the background against which patterns are viewed, can have a strong effect on forwards confusion, particularly for low-contrast patterns. In fact, the highest values of forwards confusion (by some margin) arise not from patterned wings but from the averaged treatment. Does this mean that background matching could form an alternative motion-confusion strategy? How does this compare with the results of the evolutionary simulation? E.g. it appears from supp fig 17 that some "evolved" patterns have rather low contrast – is this adaptive in the context of the simulation? It would be valuable to discuss these points in the main text.

6. The authors do briefly mention the above factors in the discussion (lines 189-194), suggesting that "Further work should also incorporate more realistic simulations that take into account … a wider range of flight dynamics, view angles and distances, and background types." But they are then somewhat dismissive of the importance: "we argue that these will likely add nuance to our findings, rather than changing any overall conclusions". Given points 3-5 above, I disagree that this can be stated with confidence. Based on the presented evidence, background can alter whether a plain or contrasting pattern is favoured by evolution. Viewing distance will determine whether a pattern is perceived as contrasting or plain in the first place. I therefore think that further consideration is needed of how much it is possible to generalise from the presented results to other viewing conditions.

7. Given that the body has a much simpler motion trajectory than the wings, I wonder whether contrast between body and wings might be an important factor contributing to motion confusion? (Not necessarily requesting a change to the manuscript here, just making a comment)

8. I found the key measures of forward confusion and sideways confusion very, well, confusing to get my head around in terms of how the values actually behave. After careful reading, it seems that "forwards confusion" is negative by default (i.e. in the absence of any misleading cues) and gets closer to zero as it increases, which I found counter-intuitive. If we're measuring confusion, would it be possible to rescale so that the minimum (no confusion) is zero rather than -1? And/or it might be helpful to add a bit more description to the main text to help orient readers to what the numbers mean. Descriptions in the methods section are helpful in this regard (such as lines 253-4) but are less prominent especially on a first read-through.

9. No results are presented to back up the statement about motion confusion being ancestral (line 194). Have the authors carried out ancestral reconstruction based on their data in figure 2b?

10. What was a typical evolutionary trajectory like over the course of the 20 generations in the simulation (line 422)? Were phenotypes converging on an asymptote by the end of the simulation? How was the duration of 20 generations chosen, and what would happen if simulations were left to run for longer?

11. All figures and statistical tests should quote sample sizes. Boxplots (e.g. Fig 1b) and error bars (e.g. Fig 3c) need defining.

12. The videos are very clear and helpful.

13. The manuscript is concise, well written and well argued. Some minor points requiring clarification or correction:
o Line 23: slightly over-generalised. Animal locomotion doesn't require limb movements, or even limbs.
o Line 31-32: can you provide examples of where butterfly wing patterning cannot be explained by pre-existing theories?
o Line 37: how is "evasive flight behaviour" characterised?
o Line 47: "in each video frame" – I found this a bit confusing as motion can't be detected from a single frame. Later it becomes clear that comparisons are between consecutive frames, but it might be useful to clarify here.
o Line 48: are the four directions defined relative to direction of travel?
o Line 62: I might be getting the baseline treatment the wrong way round, but I think that the beta values should be negative rather than positive for unpatterned compared to patterned?
o Line 88: define "var exp" – I assume "variance explained". Why are these values quite high when the R2 values are so low?
o Line 105-7: this seems to reiterate lines 94-5 unless it is based on a different analysis?
o Line 109-110: Comparisons between flapping and gliding are justified in the supplementary information but the data needs to be referenced here.
o Line 115: In Fig 2b, bars would make more sense than lines since the exact ordering of clades depends on arbitrary choices. Can Fig 2c be labelled with whether those features affect forward or sideways confusion or both?
o Line 122: selection is surely regular not random?
o Line 132: is confusion truly maximised? Or would higher values be possible? Clarify that these three outcomes were under

different selection regimes.
o Line 149: shouldn't it be over-estimate rather than under-estimate?
o Line 150: check the numerical scales for figure 3b.
o Line 158: quote the separate contributions for PC1 and PC2 so that the reader can assess their relative contribution (and/or use consistent scales on the x and y axes in fig 3b)
o Line 175: define "fluid motion vision"
o Line 223: I'm not sure what is meant by validation here, I don't see any comparison to crab behaviour or similar in Santon et al.
o Line 272: what was the orientation of the butterflies relative to the camera?
o Line 300: sentence is cut off, but this might be due to a redaction for blinding the review?
o Line 440: This seems to imply that each video frame is being treated as a separate data point. It would be helpful to state this explicitly.
o Supplementary Figure 2: check the y axis scales. I suggest writing "Raw" in sentence case rather than all-caps, to avoid confusion with the data format mentioned elsewhere.
o Supplementary Table 2: the estimates of exactly zero are a little surprising, they may be correct but just flagging for a double-check.
o Supplementary Figure 5: "values4" typo?
o Supplementary info lines 361-94 are missing an explanation/caption.

(Remarks on code availability)
The code is well-documented and clear, and I commend the authors for the openness with which they have shared their working. I have not gone as far as trying to run the code for myself, so I can't comment on the accuracy.

Referee #2

(Remarks to the Author)
It has been suggested for a while that the patterning on some animals could combine with their motion to cause visual motion processing to deliver incorrect, and therefore misleading, motion information to the visual systems of predators. Some neat and very basic demonstrations of this were made by Zanker and colleagues a few years ago using 2-frame motion (ref 9 here, but also Zanker JM. 2019. Prey capture: becoming invisible when you move. Curr. Biol. 29(18):R875–77), this has been elaborated recently to demonstrations using realistic motions that dynamic cuttlefish patterning delivers (ref 23 here: Santon et al, 2025), but there have been no systematic studies to test this hypothesis using real animal patterns. This work does that. A recent review has highlighted the crucial gap in the literature that the work described here starts to fill (Harris et al, 2025, Annu. Rev. Vis. Sci. 11:331–57; https://doi.org/10.1146/annurev-vision-101222- 051652). The research described in this paper is the first work, to my knowledge, to do this, and thus the paper is important and highly novel: just the sort of work that Nature should be publishing. It is inter-disciplinary, working across the areas of evolutionary biology, vision science and computational modelling of vision, and thus should be of interest to a wide range of audiences.

I like the work very much, but I do have some comments that I hope would encourage the authors to improve the paper. There is a huge amount of scientific work presented in this manuscript, and while I appreciate that this makes choosing illustrative figures difficult, the choices made are not always clear. I think this can be remedied easily by making some slightly different choices (e.g. the Suppl. Videos are very compelling and informative, but what they illustrate is not well described in the text).

Below follow comments on specific sections and figures.

Title:

The key point being made in this paper is that interplay between the physical motion of the butterfly wings and the butterfly pattern result in incorrect visual motion processing. May I suggest that the title emphasises this inter-play. It is the effect of both motion and pattern on early motion processing that is the crucial step forward in this research. In vision science, many would describe the motions delivered to the visual by a combination of pattern and physical motion as 'errors' in motion processing, rather than 'illusory'.

Introduction:

The introduction is engaging, comprehensive, and covers all the bases in terms of the evolution of pattern. For me, what's missing is a paragraph describing what we know about early motion processing in predators. This would broaden the interest-base for readership of this paper. The work is inter-disciplinary, but isn't really flagged as such. The elementary motion detectors (EMD) that are used for modelling here, are based on a huge body of work from the human (and insect)

vision research literature. Part of the key novelty of this work is that no-one has applied that body of work to real animal motion before in a sophisticated way. I would suggest including a short explanation for what EMD's are, and the evidence to support their existence. You have this in the Methods, but it would be great to move some of it to the Intro. In a recent paper, Santon et al (2025), describe the idea of 'generation of motion noise' as form of camouflage to explore how the motion generated by cuttlefish pattern change can potentially disrupt perceived motion. The effects described here elaborate the early motion detector (EMD) model used in that paper to give a much more detailed account of the motion of a whole range of lepidoptera species. Some of the details of the EMD motion model as elaborated in that paper, would be very usefully added here, to aid the reader in understanding more of the methods.

Methods/Results

Motion confusion and Figure 1:

The analysis of motion is performed along the cardinal directions. This seems a sensible choice, keeping the motion calculations down to a manageable size. It was not explained how forward, backward, left and right were defined. Was this with respect to the video frame, or the direction of motion of the animal? If the latter, how was the ground-truth motion direction and speed calculated? There is a clue in the figure legend of Suppl Fig 1, where the authors say "Note, as the butterfly does not fly in a straight line forwards and backwards, energy will not always correspond with the heading of the butterfly." This suggests that the motion directions are not referenced to the instantaneous direction of motion of the butterfly (its heading). This is an important technical point which could be explained in more detail. Given that the authors later do some clever modelling of flight dynamics (supplementary video 2 – Butterfly blender rendering), these details could be nailed down more carefully.

The results shown in Figure 1 clearly show the significant differences between patterned and non-patterned stimuli, but I found the data of Suppl Fig 1 much more compelling. You might want to consider promoting Supple Fig 1 to part of a full figure.

However, some motion errors (or as the authors define it, motion confusion) occur for all motions detected by EMD's. I also wondered what the EMD model would deliver for a much simpler moving stimulus? It is important for the audience to realise that errors in motion direction and speed will likely occur for any object moving. For example, if the animal body was emulated without the wings (eg. one could use rectangle to illustrate the approximate location of the butterfly body, estimated frame-by-frame).

Analysis across species and Figure 2:

I really liked the analysis across 397 species, and 757 morphotypes. This represents careful and diligent work. Figure 2a and b really need to be bigger. It could be just the pdf, but the resolution of the image is too low to illustrate the butterfly patterning well.

What is a forest plot as used in this context (figure 2c)? I'm familiar with such plots used to illustrate data such as log-odds ratios in meta-analyses. I don't think the authors are using the term in the same way here. Could the authors please explain what they are plotting in figure 2c? There are some details of 'Random Forest" modelling given in the Methods, but for the uninitiated (including me) there is not a clear explanation of what is plotted, nor why.

Evolving butterflies using genetic algorithms and figure 3:

Again, great work. The power of this work is that the genetic algorithms used do indeed 'evolve' the kinds of patterns that have been suggested to be effective at disrupting the overall motion signals. For the first time, to my knowledge, the results here show that they do. That the evolved patterns are similar to those of real butterflies is also a really important result.

I found the interaction with overall wing shape particularly interesting. The method is very nicely illustrated in Suppl. Video 3.

However, the text in this part of the Results section is difficult to follow without a careful reading of the Methods section. I suggest the authors take a look at this and work towards a simple explanation in this section, summarising what was done, and what was measured, so that is clear what each part of the figure is illustrating.

For example, it would be useful to describe in more detail what the PCA hulls graphs illustrates (Figure 3b). The data of figure 3C are also important, demonstrating how close some natural butterfly patterns are to these genetically evolved optimal patterns. These details are elaborated nicely in the Methods section, but some could really do with being promoted to the main text so that the crucial points can be appreciated by a non-technical audience.

Typos and other small points:

Line 300: sentence about approval is not finished. Is this where any ethical approval should be mentioned?

Line 501: Is the code for the elaboration of the EMD model available? This would be a very useful addition to open-source code that would allow other groups to apply this form of modelling to other natural motions, and indeed to other contexts including the design of human environments (e.g. driving, trips hazards in urban spaces, etc).

(Remarks on code availability)
I have looked at the code materials, not reviewed the code in detail, nor tried to run it. I hope that other technical referees with more current coding skills will be willing to do that.

I may have missed it but, Is the code for the elaboration of the EMD model available as a stand-alone? I think it sits in the ImageJ code folder, but that seems to include all sorts of material that might come from other projects too. I didn't see any README files there. This would be a very useful addition to open-source code that would allow other groups to apply this form of modelling to other natural motions, and indeed to other contexts including the design of human environments (e.g. driving, trips hazards in urban spaces, etc).

Referee #3

(Remarks to the Author)
The paper ‘Butterfly wing patterns create powerful illusory motion cues’ presents three strands of investigation into the hypothesis that butterfly wing patterns can function in motion camouflage. Misleading predators about the true direction or speed of movement could then reduce the probability of successful attack. This is what many in the field call dazzle camouflage, thought the authors have chosen not use this term.

Dazzle camouflage/pattern based motion illusions have been investigated for several decades with a complex and not yet especially solid evidence basis (meta-analysis in Ref#5, also reviewed in Tan et al. 2024 https://doi.org/10.1111/brv.13044 which would be a useful citation). Lab experiments (generally humans or birds catching artificial prey on monitors) have had low replicability, and there is currently little evidence that 1.) this class of camouflage mechanism can reduce predation in natural conditions and 2.) that it is utilised by any animals, though it has been suggested for several species and groups of species. To my knowledge, butterfly wing patterns have not yet been suggested as functioning in motion confusion, so the proposal that is explored here is novel. I comment on each of the three elements in turn:

1.) Analysis of high-speed videography of several butterfly species using elementary motion detectors suggests that illusory motion signals are produced.

EMDs are simple local motion detectors. Motion of contrasting elements either within a wing, or between a wing and a background, on a different vector to the global motion (such as a vertical wing stripe on a flapping wing) will generate an ‘illusory’ response. Dealing with noise (variability) in local motion signals is a challenge for all visual systems when trying to estimate global motion, and they are adapted to do so. What matters in a predator-prey interaction is how noise affects the global motion percept – when do errors occur? This depends on how local motion signals from different areas of the object are combined. Intuitively the accuracy of this should increase as the local motion signals are more consistent, which is the argument made here (L51) and in many global motion models, but this may not be the case – Green & Pratte (2022 10.1037/xhp0000971), for example, provide evidence that global motion perception either happens with a high degree of

precision, or not at all. The global motion model used (Michelson contrast between energy in different directions) in this study is very basic and should be properly justified as having a basis in biological reality as it is key to conclusions. Better, alternative global motion perception models could be evaluated. The gold standard would be experimental evidence that the direction and strength of local motion signals correspond to predator targeting errors. I appreciate that this is probably another project, but without it, the conclusions of the current work are inevitably less convincing.

2. Wing patterns of European butterflies can have high levels of motion confusion.
This is a very interesting analysis that investigates EMD outputs of a model receiver viewing European butterfly wing patterns on a 3D animated butterfly. It then identifies what pattern features are associated with modelled illusory movement, and finally examines phylogenetic patterns in the degree of motion confusion. It concludes that illusory local motion signals are common and widespread in the species studied, and that motion camouflage could therefore be the function associated with otherwise unexplained variation in wing pattern. The methods used in this analysis are excellent, as are the figure and supplementary movie, but I found the premise on which the conclusions are built more problematic.

The paper argues that colour pattern function in European butterflies is not generally well explained by current hypotheses. This implicitly supports the decision to not evaluate the motion confusion hypothesis against alternative hypotheses for wing colour pattern function. However, I would argue that this is not an accurate characterisation of current understanding. Camouflage, thermoregulation, warning, deimatic and sexual signal functions have all been demonstrated in different species of European butterfly. For other groups of species, the evidence is very limited and uncertain (e.g. Pieridae Lyytinen et al 1999 https://doi.org/10.1023/A:1011081800202) or absent entirely. Thus, I would argue that uncertain function for many species has more to do with a lack of investigation rather than research ruling out hypotheses leaving the function enigmatic after thorough research.

Not properly considering alternative functional explanations may lead to erroneous conclusions. Some functions have similar form requirements while others predict different phenotypes. For example, there is evidence that wing pattern in the European swallowtail Papilio machaon, a species with high motion confusion score, is part of a deimatic signal (Olofsson et al 2021 https://doi.org/10.1371/journal.pone.0047092), as might be the peacock butterfly Inachis io (Vallin et al. 2005). The white band on the white admiral, another species with a high motion confusion score, is thought to be a disruptive camouflage marking (Stobbe & Schaefer 2008). Disruptive camouflage, deimatic, aposematic and sexual signalling functions are all supported by high contrast markings, so as pointed out (L171), the evidence presented is equivocal whether motion confusion, disruptive camouflage, a signalling function, or multiple functions are in play. The solution to this problem would be testing alternate comparative hypotheses made by the different hypothesised functions. In which ecological conditions would high motion confusion be expected to evolve? In which would sexual signals be expected to evolve?

3. Artificial butterflies evolved for motion confusion resemble real butterflies.

This is a solid analysis using a very nice approach that I find quite convincing, but with the same issue. It would be greatly strengthened if it was able to show that motion confusion evolved artificial butterflies were more similar to European butterflies than warning signal or disruptive camouflage evolved artificial butterflies etc. I would suggest that comparisons are only made between species that have high scores on a metric, as species with low scores are unlikely to be using the potential function.

I would also recommend toning down the ‘independence’ of each investigation (L9 and elsewhere). They all rely on the same motion detection models, and they use the same set of parameters (as pointed out on L189). I think this is fine – there are higher priority additional analyses in my view (as suggested above) than sensitivity analyses over different flight behaviours and viewing directions etc. – there is no strong need to say they are independent lines.

In summary, I think this is a novel and exciting idea, and therefore of course somewhat speculative. Applying three approaches to investigate is a strength of the paper, and the methodologies of each approach are all excellent. However, for a paper of the highest quality, I think the evidence provided by each approach could and should be more convincing in support of the conclusion.

(Remarks on code availability)
I was unable to spend lots of time reviewing the code, but I was able to easily understand and access all of the data and analyses that I tried. The organisation and commenting seemed exemplary.

Referee #4

(Remarks to the Author)
Camouflage has a long history as one of the primary anti-predator strategies, yet it is well known that motion breaks camouflage. However, the mechanisms by which prey avoid attacks once they start moving remain poorly understood, and empirical evidence is limited. This study makes a significant step toward addressing this problem. The authors investigate how butterfly wing patterns interact with flight dynamics to generate illusory motion cues that may mislead predators. To do so, they combine high-speed recordings of butterfly take-offs with biologically inspired motion modelling, comparative analyses across hundreds of species, and in silico evolutionary simulations. This is a remarkably clever and creative piece of work. The multipronged approach is impressive, and the idea that color patterns may actively exploit limitations in predator motion perception has the potential to shift how we think about pattern–motion interactions in nature.

However, several conceptual, methodological, and interpretational issues (detailed below) make it difficult to fully evaluate the strength of the evidence in its current form. Clarifying how the modelling results translate to biologically meaningful effects, better defining the mechanism relative to existing concepts such as motion dazzle, and explaining the rationale behind key analytical decisions would greatly strengthen the manuscript. As it stands, I am unable to recommend publication at this stage, but I believe the work has the potential to make a substantial contribution after revision.

General comments
1. The effects described in the manuscript are demonstrated primarily through modelling, but it is unclear how well these results translate to real predator–prey interactions. I am fully aware of the practical challenges involved in testing these ideas with real predators. However, previous studies on motion-based antipredator strategies have routinely used human observers as a first step. Incorporating a similar 'simulated predator experiments with humans' at least would greatly strengthen the biological interpretation of the results. Alternatively, the authors could at least translate their modelling outputs into more interpretable quantities (e.g., how much the observed forward–sideways confusion would translate into shifts in perceived speed or direction) so readers can better assess the magnitude and ecological relevance of the effects. As it stands, without empirical validation through experiments, the inferences remain difficult to evaluate. For example, while the modelling shows that certain patterns produce forward-confusion, it is unclear whether this level of confusion is sufficient to reduce attack success in any meaningful way.

2. The current modeling results show that patterned wings generate stronger illusory motion cues than an unpatterned treatment, but it does not demonstrate that they are more different than camouflage. This is an important issue because motion usually breaks camouflage, and patterned wings could represent an alternative defence once the animal is moving. The authors do not address this point directly, and the backgrounds used in the modelling are always contrasting with the butterflies. Yet this question is central to understanding the broader relevance of the mechanism. Discussing this limitation, or clarifying that the study is not making claims about effectiveness relative to crypsis, would make readers more aware of this aspect.

3. Throughout the manuscript, the proposed mechanism ('motion confusion') is presented as if it is fundamentally different from motion dazzle, even described as a 'hitherto unknown' visual defence strategy. However, the effects described here, including changes in perceived speed and direction produced by high-contrast patterning, closely resemble classic motion-dazzle mechanisms demonstrated in other animals. It would be helpful for the authors to clarify whether their findings demonstrate a genuinely distinct mechanism, and if so, how it differs from existing explanations. Alternatively, do the results primarily offer empirical support for longstanding dazzle hypotheses when viewed through the lens of butterfly flight dynamics and EMD analyses? Clarifying this would help readers understand the novelty and avoid conceptual ambiguity.

4. Finally, several key analytical choices are not fully justified (e.g., viewing angle), and clearer explanations of why particular decisions were made would strengthen the manuscript (see specific comments).

Specific comments
1. Line 17 & 20: Highly relevant papers to cite.
Ioannou, C.C. and Krause, J., 2009. Interactions between background matching and motion during visual detection can explain why cryptic animals keep still. Biology Letters, 5(2), pp.191-193.
Tan, M., Zhang, S., Stevens, M., Li, D. and Tan, E.J., 2024. Antipredator defences in motion: animals reduce predation risks by concealing or misleading motion signals. Biological Reviews, 99(3), pp.778-796.

2. Line 37-38: This sentence is difficult to follow. It is not clear whether the authors are suggesting that species with striped wing patterns typically also possess eyespots and evasive behaviours, or whether eyespots are intended as an independent example of how wing patterns and flight behaviour might interact to affect motion detection. Note that deflection via eyespots does not require any movement component. So, this is misleading. Moreover, the cited study by De Bona et al. 2015 (Reference 17) addresses the intimidation (predator mimicry) hypothesis, not deflection. The paper below might be relevant for deflection.
Prudic, K.L., Stoehr, A.M., Wasik, B.R. and Monteiro, A., 2015. Eyespots deflect predator attack increasing fitness and promoting the evolution of phenotypic plasticity. Proceedings of the Royal Society B: Biological Sciences, 282(1798), p.20141531.

3. Line 40: I do not think this statement is accurate. Most avian predators are diurnal, so nocturnal moths are unlikely to be widely targeted by birds while in flight. Predation risk from birds is largely associated with daytime resting, where moths rely on crypsis rather than evasive flight.

4. Line 27-42: The final section of the introduction is not very compelling. It mixes several distinct mechanisms (e.g., deflection, intimidation, evasive behaviour) that do not necessarily affect motion misperception. As a result, it is difficult to see how these examples build toward the proposed hypothesis. I recommend rewriting this section so that the proposed mechanism is stated more clearly and the narrative focuses directly on how wing patterns and flight dynamics are expected to create misleading motion cues. For instance, would the authors consider their proposed mechanism to be distinct from, or a specific form of, motion dazzle? Clarifying this would help readers understand whether the idea is potentially novel or how it fits within existing mechanisms.

5. Line 52: The term 'deflection' is used somewhat loosely here. It can refer to misdirection toward less vulnerable body parts (e.g., wing margins; sensu stricto) or to mechanisms that misdirect attacks away from the animal entirely (which I assume is

what the authors intend). Please clarify which meaning is meant. If the latter, it may be clearer to refer to this as ‘misdirected attacks’. It may be worthwhile to define this in the last section of the introduction.

6. Line 57: The rationale for using only unpatterned treatments is not clear. It may be more appropriate to include patterned prey with the same overall contrast but without stripes (e.g., blotches or random patterns)? This would provide a more appropriate comparison.

7. Line 64-65: I may have missed it, but it is not clear that the results actually show ‘estimates of speed,’ as the figures only present forward and sideways confusion. Is this based on forwards-energy? Also, does it imply that a contrasting but unpatterned prey could be equally effective in misleading motion?

8. Line 109: It is not clear where these results are presented. Could the authors point to the relevant figure or supplementary material with numbers?

9. Line 133: Please move this definition of forward energy to the section above where the term is already introduced.

10. Line 146: P-value for random selection missing.

11. Line 149: I may have misunderstood, but this seems contradictory to the earlier interpretation where the authors suggest that unpatterned white prey should have a higher estimated speed (Line 65). Should this instead refer to overestimated forward speed?

12. Line 153: The x-axis label for panel c and the corresponding legend might be confusing, as the treatment ‘Natural’ is also included. Please clarify what ‘Natural’ represents in the legend.

13. Line 164 & 187-189: It is not intuitive why one would expect illusory motion to result in deflection of attack. Is this the same as what has been proposed for lizards (Murali et al., 2016)? Could the authors expand? Alternatively, I suggest rewriting this as ‘misdirected attacks’ rather than classical deflection (i.e., ‘attacks redirected to less vulnerable parts’). See my comment above on this.
Murali, G. and Kodandaramaiah, U., 2016. Deceived by stripes: conspicuous patterning on vital anterior body parts can redirect predatory strikes to expendable posterior organs. Royal Society Open Science, 3(6), p.160057.
Murali, G., Merilaita, S. and Kodandaramaiah, U., 2018. Grab my tail: evolution of dazzle stripes and colourful tails in lizards. Journal of Evolutionary Biology, 31(11), pp.1675-1688.

14. Line 266 onwards: It would be helpful to clarify why recordings were restricted to take-offs. Mid-flight wingbeat patterns can differ substantially. Please provide justification for limiting the recordings only to take-offs.

15. Line 267: It would be helpful if the authors could include the rationale behind selecting these five species for this analysis. Do these species represent the major colour-pattern morphotypes observed, and if so, how was this determined?

16. Line 274: For how long?

17. Line 293: Please include the number of individuals per species. Also, the actual number of video trials used in the analysis (i.e., to generate Figure 1) per species. I presume they are the same across treatments.

18. Line 311: Why were only dorsal patterns analysed and not both dorsal and ventral? Many species vary in ventral coloration and appearance change across viewing angles, so please justify this.

19. Line 326: The 3D flight rendering appears to be based on a single species, whereas the colour-pattern analyses encompass the full dataset of over 750 morphotypes. Because different species exhibit distinct flight dynamics, it is unclear whether a rendering based on one species can adequately represent the broader diversity being analysed. The authors should clarify the rationale for restricting the 3D component to a single species and discuss how this affects the generality of the conclusions drawn from the modelling. At the very least, why not use the flight dynamics of all seven morphotypes that were recorded, assuming these represent colour-pattern–related differences in flight behaviour?

20. Line 346: Please add justification for why the analysis was restricted only to one viewing angle.

21. Line 393: Please provide more detail on the key parameters used in the simulated evolution analyses (e.g., number of prey, population size, mutation rates). Please clarify how these parameter values were chosen, and how sensitive the results are to the specific values used. If these details are already included in the Supplementary Information, please link to the specific table or section.

22. Line 471: What were the predictor and response variables in this analysis, and which random effects were included?

23. Line 478: Could the authors briefly mention what these metrics represent? Are they the same as those described in the section on ‘butterfly wing features’?

24. General comment: Please specify the exact supplementary table or figure numbers when referring to them, as there are many, and the current references are unclear (e.g., Line 146).

(Remarks on code availability)
I reviewed the code provided by the authors, and the repository is generally well organized. The README file offers clear instructions for installation and execution. However, I was unable to install and run the main EMD module, which appears to be missing. I spent considerable time locating it but was not successful. As a result, I could not reproduce the analyses that rely on this component. Apart from this issue, the remaining scripts function as described.

Referee #5

(Remarks to the Author)
This work offers an original and convincing interpretation of how butterfly wing patterns and movements might serve to confuse predators. The methods are appropriate, and the main conclusions are convincing. The findings are both of broad significance and (at least to me) not a priori obvious. Overall, this is the most significant recent work on how defensive coloration works to conceal and confuse, and complements the wider literature on the evolution, development and function of butterfly wing patterns.

The key finding - expressed by the 'forwards confusion' measure - is that the relatively low beat frequency of butterfly wings combined with their pattern produces a forward motion signal that differs from that produced by a gliding butterfly (simple translation). By comparison the sideways confusion, forwards (motion) energy parameters (Fig 3a), and apparent speed need more explanation.

i) A gliding butterfly with stripes produces 'sideways confusion' (line. 109) presumably due to the 'aperture effect' (Adelson & Movshon. Nature 300:523, 1982). Sideways energy (l. 248) sums left and right energy, but for a bilaterally symmetrical pattern would directional signals cancel out, so the butterfly would not appear to deviate from its actual path (c.f. l. 53)?
The ms. could be more clear about the relationship between 'motion dazzle' (e.g. Ref 5) produced by simple translation (e.g. gliding) and the effects of wing movements. More generally, elementary motion detectors do not directly measure velocity, so the relevance of findings that forwards (motion) energy is greatest for a unform white butterfly (Fig. 3a), and inconsistently (?) that higher contrast [markings] resulted in higher estimates of speed (line 64) need more justification and explanation to support the main argument.
Fig 1 is quite difficult to understand without reference to the text (e.g. lines 246-250). Can more explanation be included in the legend, or even 1 added to the confusion parameter so that 0 (rather that -1) corresponds to no effect of wing movement.

(Remarks on code availability)

Version 1:

Reviewer comments:

Referee #2

(Remarks to the Author)
This is a much-improved paper compared with the version I originally commented on.

In particular, the behavioural experiments using humans to test the EMD model predictions is a valuable addition, making the interpretation of the modelling results much more robust (though see my last point at bottom of this paragraph). The inclusion of the new figure 2c is a great way to illustrate the human behavioural data in terms of error in attack location, but it would be more valuable if the authors referred to it when they describe the human behavioural experiment (lines 164-171). Further, there is no additional section in the Methods describing the human experiments. These are really important, as the authors highlight in the Response to Reviewers, so please add some details to the Methods, even if that is simply a sentence pointing to the details in the Supplementary Materials. One point of clarification here: the authors state that feedback was given to participants during the training tutorial, but not whether it was given in the main experiment (I assume not, but this needs to be made explicit). Finally, the authors state "Contrary to our predictions, sideways-confusion actually decreased the distance perpendicular to the butterfly (tightening attacks) and caused attacks to be further forward." That is interesting. It does potentially undermine the comparison between behavioural and modelling data. Some interpretation of this issue would be valuable here.

The Introduction is tighter now, and will likely fulfil most of the reviewer requirements suggested in response to the previous version. Yet it still offers an interesting read to those who are less expert.

The additions showing motion confusion directions in Figure 1 aided understand of the 2 dimensions of confusion, making this figure much easier to digest.

I liked this work when I first read the paper, I think it provides a much stronger contribution now and I recommend acceptance in Nature.

Some small points:

Line 131: I’m still non the wiser about what random forest models are. For the general reader, it would be useful to add a reference here giving an example of how they work in more detail. It’s in the Methods, but good to add to main text too.

Line 361: end of sentence missing.

(Remarks on code availability)

Referee #3

(Remarks to the Author)
The behavioral experiments complete the study, which provides a remarkable integration of convergent strands of investigation. The evidence for the proposal is now convincing. It changes current understanding and will inspire work for years to come. I congratulate the authors.

The many other changes made by authors have substantially improved the paper, and where suggestions were not implemented, the response letter is persuasive. This was generally because they are outside the scope of what is feasible for this study, which on reflection I agree with. I expect reviewers got excited about the idea and in part identified avenues for future research - I think this can be taken as an indicator of the influence the paper will have.

(Remarks on code availability)

Referee #4

(Remarks to the Author)
I was reviewer 4 in the first round of this manuscript. The authors have made substantial improvements, particularly by incorporating experimental validation of the model outputs. This directly addresses a major concern raised previously and significantly strengthens the paper. The authors have also made a commendable effort to incorporate and respond to all other reviewers’ comments. That said, I still have one concern regarding the newly added results.

The capture experiments are an important component of the manuscript, but their interpretation raises an important concern. The results focus primarily on attack distribution, and although the Supplementary Information presents hits across body parts, overall capture success or failure rates are not reported. These would be critical for evaluating whether the observed confusion translates into a meaningful reduction in capture success, rather than simply increasing error, which is central to the biological relevance of the proposed mechanism. I suggest that the authors include these results, at least in the Supplement, for example by reporting the proportion of successful captures across treatments.

Specific comments
1. Line 11-12: Would ‘high-contrast butterfly-like wing patterns’ be more accurate here, given that the opening sentence highlights the importance of high-contrast patterns in interfering with motion perception?

2. Line 36: Should it be ‘motion misperception’ instead of ‘motion detection’? Because the study shows that these patterns produce misleading motion cues rather than preventing motion detection altogether.

3. Line 38: I raised this point previously, but it remains unclear. The link between motion and any enhancement of deflection via eyespots during movement is not well explained. Are the authors suggesting that eyespots could also enhance confusion during motion?

4. Line 59: ‘real wing patterning’ change to ‘natural wing patterning’ to keep it consistent.

5. Line 65: Should this be higher forward energy? The authors appear to refer to these results again in the next section (lines 101–102), so it would help to define this upfront.

6. Line 96-100: It may be helpful to include an illustration in Figure 2 or in supplement highlighting these features (e.g., numbered and labelled alongside). For example, it is not immediately clear without referring to the Supplement what is meant by increased forewing contrast or how it differs from higher hindwing luminance.

7. Line 110-112: It is not entirely clear how these results differ from those presented above for features associated with sideways-confusion (lines 100–101). Are these results specific to the two families considered here? If so, why are features associated with forward confusion not presented as well? Please clarify.

8. Line 124 & 138: From the figure, it appears that the butterfly with the second-lowest forward confusion (Gonepteryx sp) shows a higher attack density entirely outside the body outline, which could be misinterpreted. It may be helpful to include a brief explanation of why elongated or elliptical attack patterns are expected when patterns produce forward-confusion in text or in the legend.

9. Line 125: Please provide the specific supplementary table number here.

10. Line 128: Is this confusion quantified for flapping only, or for a mix of flapping and gliding? Please give some info in the legend.

11. Line 132: Unclear which points the authors are referring to in the phylogeny.

12. Line 154: Should ‘patterns that were effective’ be replaced with ‘patterns that produced’ here? This is because effectiveness is not directly quantified, and the results are instead presented in terms of metrics such as forward confusion.

13. Line 208: The study below may be more relevant and recent, as it tests both capture success and speed perception of multiple striped objects within the same experimental setup.
Kodandaramaiah, Ullasa, et al. "What makes motion dazzle markings effective against predation?." Behavioral Ecology 31.1 (2020): 43-53.

14. Line 251-252: It would be helpful to justify how well this value generalises across a range of relevant predators.

15. Line 285-287: This section ends abruptly. Seems like some text may be missing. There are also formatting inconsistencies throughout the Methods, and variable names are not used consistently, giving an overall impression of incomplete or insufficiently checked sections. Please review carefully.

16. Line 349 onward: Please clarify which type of flight was used in the main analysis (flapping vs gliding).

17. Line 384: Were the average grey and white butterflies only included in the RF models? The main figure appears to show confusion values only for natural patterns, so it is not entirely clear why these comparisons were included.

18. Line 424: Please include details of the human-based capture experiments here, as this section is entirely missing.

19. Line 506: Again, the statistical analyses used to test attack distributions are not described. Please provide these details.

20. Line 507: “LMs” or “LMMs”? Please define.

21. Line 513: By ‘natural butterflies’, do the authors refer to all 757 morphotypes rather than the seven used in the initial analysis? Please clarify this in the text throughout.

(Remarks on code availability)
The code was reviewed in the previous round and appears sufficiently detailed to allow the results to be reproduced.

Referee #5

(Remarks to the Author)
I have reviewed a previous version of this ms. and am satisfied that the main findings are sound, original and significant. The insight is not intuitively obvious. The authors might mention that the question of how velocity is estimated from motion energy, and the extent to which sideways confusion is due to the aperture effect, but this points do not critically alter the conclusions. What we have is a wonderful insight into the function of butterfly flight, wing shape and coloration.

(Remarks on code availability)
None

Version 2:

Reviewer comments:

Referee #2

(Remarks to the Author)

(Remarks on code availability)

Referee #4

(Remarks to the Author)
The authors have satisfactorily addressed my previous concerns, especially regarding the interpretation of the behavioural capture experiments. I am happy with the revisions and have only a couple of minor comments below.

1. For Supplementary Fig. 32, it would be helpful to clarify in the caption whether the bounding area was calculated for each

frame of the animation and then summarized across frames.

2. The caption for Supplementary Figure 33 is missing.

(Remarks on code availability)

Open Access This Peer Review File is licensed under a Creative Commons Attribution 4.0 International License, which permits use, sharing, adaptation, distribution and reproduction in any medium or format, as long as you give appropriate credit to the original author(s) and the source, provide a link to the Creative Commons license, and indicate if changes were made.
In cases where reviewers are anonymous, credit should be given to 'Anonymous Referee' and the source.
The images or other third party material in this Peer Review File are included in the article's Creative Commons license, unless indicated otherwise in a credit line to the material. If material is not included in the article's Creative Commons license and your intended use is not permitted by statutory regulation or exceeds the permitted use, you will need to obtain permission directly from the copyright holder.
To view a copy of this license, visit https://creativecommons.org/licenses/by/4.0/

Referees' comments:

Referee #1 (Remarks to the Author):

“Butterfly wing patterns create powerful illusory motion cues”

The manuscript explores how butterfly wing patterns affect the accuracy of motion cues. The authors make use of an “Elementary Motion Detector” visual model that quantifies directional motion “energy” generated by pixel comparisons between consecutive frames of a video. Any directional energy values which indicate motion in directions other than the true direction of motion are considered to contribute to motion confusion, which the authors argue has the potential to mislead predators into mis-targeting their attacks on moving prey.

The authors apply their model to three different data sets: videos of real butterfly take-off motion, simulated butterfly flight of a wide range of different species’ patterns, and artificially generated patterns that undergo simulated selection for motion confusion. They find that many natural butterfly patterns have higher values for motion confusion than plain patterns, and that artificial patterns selected for motion confusion increase their similarity to natural patterns.

1. The manuscript is likely to be of wide interest, bringing a clear strategy for addressing what has been an under-explored aspect of animal colour patterns. This comes in the context of huge research interest in butterflies as a model for animal colour patterning, and with the possibility of applying the technique to a range of other taxa. The key question in my mind while reading the manuscript is: does “motion confusion” as measured here actually confuse predators? I appreciate that this is a slightly separate question to the one the authors have asked, and they do acknowledge the need for behavioural experiments (line 188). Although references are made to the EMD model being “well validated” (lines 184, 223), as far as I can see, only two of the referenced studies (36 and 37) compare it against receiver behaviour, both of which involve humans responding to computer-generated cues. How confident can we be that predators targeting flying prey use analogous methods for judging motion? In my opinion, this has the potential to be a classic and extremely well-cited paper if future behavioural experiments back up the model, but I’m not sure that is guaranteed.

Thank you for your positive assessment of our work. We are pleased to say that we have now completed behavioural validation of our results using human ‘predators’ trying to catch moving butterflies on a touch screen. Importantly, our methods incorporate temporally aliased videos on high speed monitors, avoiding motion artefacts (such as the ‘wagon wheel’ effect) that have not been controlled for in any previous studies. Our results match EMD model predictions: butterflies that are

predicted to have a high level of forward confusion have a more elongated kernel of click density, meaning that participants were more likely to attack behind the butterfly, and our statistical modelling confirms that the forward confusion metric is a significant predictor of how far back a participant's attack will be. Further details are available in the main manuscript (Figure 2, lines 56-68 and Supp material section 4), along with detailed descriptions of the experiment in the Supplementary Material (Section 4).

2. The authors have generally been careful in their language to avoid over-stretching the conclusions (e.g. line 163 "consistent with selection for motion confusion", line 166 "how motion confusion could operate"). On the other hand, this might be pedantic, but can we say that the motion illusion is "powerful" (line 1) when we don't know its effect? Can we say that motion confusion is an evolutionary "strategy" (line 12) when we don't know if it could be simply a biproduct of selection for warning colouration? For consideration for future work: are there differences between the patterns we would predict under motion confusion vs aposematism that could be used to separate the two strategies?

We believe our behavioural validation helps to support the use of the word "powerful", as we now have evidence that patterning can indeed cause behavioural effects that would have a substantial effect on mortality (e.g. see the 38% of body length misdirection effect size). We would also argue that the evidence we present in the manuscript is inconsistent with motion confusion being simply a by-product of warning coloration: for example, we find that butterflies rich in long-wavelength colouration (i.e. red), commonly associated with aposematism, are among the least motion confusing. We agree that better understanding the relationship between motion confusing and aposematic patterning is an important avenue for future research, but we would caution against setting these explanations up as mutually exclusive, as it is of course the case that one type of patterning could have multiple functions.

3. The manuscript is innovative in its approach, using sophisticated image analysis to generate motion confusion measures. The use of animation enables the authors to expand their taxonomic coverage from the smaller numbers of species used in live video capture, to encompass a comprehensive range of European species. This is a nice way to overcome the practical limitations of data collection, while keeping measurements grounded in reality. The methods generally appear to be very well implemented, with care taken to account for the visual systems of realistic signal receivers (passerine birds, which are justifiably assumed to be key selective agents here). However, all modelling is based on images filtered to a certain level of visual acuity (line 229). Although this value has a biological basis, it is an angle, and so the amount of spatial information visible depends heavily on the viewing distance. On what distance were the results based, and how robust are the results to changes in that value?

The results assume a distance of 34 to 193cm (depending on the size of the butterfly, from 1.08cm to 6.13cm respectively) to a small bird with spatial acuity of 6 cycles-per-degree. We chose these distances to be as biologically realistic as possible: they are close enough to the target that an avian predator would be likely to make their feedforward, ballistic attack. In addition, closer distances are challenging to model, as they require very high frame rates to avoid motion artefacts (this viewing distance already requires simulation at 2,000 fps). Longer viewing distances lead to the stripes no longer being resolvable, and are therefore irrelevant for the current hypotheses. We have scaled viewing distances to body-size (rather than fixed across all butterflies) because predator detection distances and responses will also scale with butterfly size.

We are grateful that the reviewer notes that our simulation parameters are grounded in reality. Here and elsewhere reviewers suggest exploring different model parameters such as viewing distance, viewing angle, background, flight dynamics, etc. While many of these questions may add interesting nuance to our findings, the additional work in terms of computing power and time (particularly when considering combinations of variables) would not be feasible given the already ambitious level of processing undertaken.

4. Animation of butterflies was based on the flight pattern of a single species (line 327). The authors adjust for speed and wingbeat frequency but the “shape” (I can’t think of a better word) of wing motion is constant across all animated species. I would imagine that there might be a strong interaction between wing morphology and flight patterns. How much do species vary in their flight patterns, and how much effect does the flight pattern have on the measures of confusion? Would it be valuable to carry out a test of robustness to changing the way in which the motion is animated?

Our behavioural validation work incorporates multiple flight trajectories (see Supplementary Materials, Section 4) and the effects are robust across different trajectories. We also focus on the wing dynamics at the point of take-off, when butterflies are likely to be most vulnerable to attack. On take-off, butterflies will be most likely to rely on high-amplitude clap-and-fling dynamics (that we have modelled), which are likely to be more conserved among species than later steady flight dynamics that vary among taxa. In addition, high temporal and spatial resolution 3D data for butterfly free-flight wing dynamics and flight patterns do not currently exist. As above, while there are clearly many interesting questions to ask about flight patterns, we would suggest these are beyond the scope of the current work.

5. There are some interesting effects of background matching that are evident in the supplementary information (lines 138-141 and Supp Fig 8). It seems that background

matching, and hence the background against which patterns are viewed, can have a strong effect on forwards confusion, particularly for low-contrast patterns. In fact, the highest values of forwards confusion (by some margin) arise not from patterned wings but from the averaged treatment. Does this mean that background matching could form an alternative motion-confusion strategy? How does this compare with the results of the evolutionary simulation? E.g. it appears from supp fig 17 that some "evolved" patterns have rather low contrast – is this adaptive in the context of the simulation? It would be valuable to discuss these points in the main text.

Natural backgrounds are always heterogeneous in terms of pattern, so an animal moving across these backgrounds would always be visible, making a background-matching strategy impossible. In Supplementary Figure 8, we would caution that while there are indeed a number of outliers, they are a very small percentage (9%) of the total. These outliers would be expected due to interactions between the butterfly and local (random) background features, which on occasion would be expected to combine and create backwards motion cues by chance. Indeed, we have preliminary data looking at confusion metrics on different backgrounds (see below), and it is clear that while the absolute numbers do vary, the relative ordering of the effects is fairly stable, suggesting that motion confusion effects are not strongly dependent on background type.

Regarding the contrast of evolved prey, it is important to note that these patterns have been put through a Gaussian spatial filter (simulating viewing distance and controlling for temporal aliasing effects), which has the effect of artificially reducing contrast. The unblurred evolved patterns have higher contrast (but we only show the wings as they were processed through the EMD).

6. The authors do briefly mention the above factors in the discussion (lines 189-194), suggesting that "Further work should also incorporate more realistic simulations that take into account … a wider range of flight dynamics, view angles and distances, and background types." But they are then somewhat dismissive of the importance: "we argue that these will likely add nuance to our findings, rather than changing any overall conclusions". Given points 3-5 above, I disagree that this can be stated with confidence. Based on the presented evidence, background can alter whether a plain or contrasting pattern is favoured by evolution. Viewing distance will determine whether a pattern is perceived as contrasting or plain in the first place. I therefore think that further consideration is needed of how much it is possible to generalise from the presented results to other viewing conditions.

We hope that our points above help to clarify our reasoning. In brief, while we have only pilot tests of orientation and background effects, they support our assertion that the motion confusion effects, particularly for highly confusing species such as swallowtails, are remarkably robust. Exploring any one of these dimensions in full

would be a considerable undertaking, and beyond the scope of the current study (which we believe is already extremely wide-reaching).

7. Given that the body has a much simpler motion trajectory than the wings, I wonder whether contrast between body and wings might be an important factor contributing to motion confusion? (Not necessarily requesting a change to the manuscript here, just making a comment)

Indeed - in our evolution simulations, it is interesting to note that the contrast of the body always decreased when selecting for motion confusion. Pilot evolutionary lines included the inverse of forwards-confusion which resulted in butterflies with white bodies. This aligns with the observation that almost all butterflies have low-contrast body coloration.

8. I found the key measures of forward confusion and sideways confusion very, well, confusing to get my head around in terms of how the values actually behave. After careful reading, it seems that “forwards confusion” is negative by default (i.e. in the absence of any misleading cues) and gets closer to zero as it increases, which I found counter-intuitive. If we’re measuring confusion, would it be possible to rescale so that the minimum (no confusion) is zero rather than -1? And/or it might be helpful to add a bit more description to the main text to help orient readers to what the numbers mean. Descriptions in the methods section are helpful in this regard (such as lines 253-4) but are less prominent especially on a first read-through.

We have updated Figure 1 to include arrows and its caption to aid understanding of forward and sideways confusion. Because the metrics are based on Michelson contrast, we want to keep them on the same scale to aid readers who are familiar with this approach. We hope the changes to the text make it easier to interpret.

9. No results are presented to back up the statement about motion confusion being ancestral (line 194). Have the authors carried out ancestral reconstruction based on their data in figure 2b?

We have not carried out ancestral state reconstruction, so have now removed this sentence as it was misleading.

10. What was a typical evolutionary trajectory like over the course of the 20 generations in the simulation (line 422)? Were phenotypes converging on an asymptote by the end of the simulation? How was the duration of 20 generations chosen, and what would happen if simulations were left to run for longer?

We have now included a graph in the supplementary material to help answer this question (See section 3.iii). This clearly shows that the phenotypes for most

populations were converging on an asymptote by the end of the simulation. We chose the duration of 20 generations based on previous research that has shown this is generally sufficient (e.g. https://www.biorxiv.org/content/biorxiv/early/2025/03/06/2025.03.01.640995.full.pdf, https://academic.oup.com/evolut/article/76/5/870/6881792).

11. All figures and statistical tests should quote sample sizes. Boxplots (e.g. Fig 1b) and error bars (e.g. Fig 3c) need defining.

We have now provided the sample sizes and error bar descriptions for all plot figure legends.

12. The videos are very clear and helpful.

Thank you!

13. The manuscript is concise, well written and well argued. Some minor points requiring clarification or correction:

o Line 23: slightly over-generalised. Animal locomotion doesn't require limb movements, or even limbs.

We have added "often". L24

o Line 31-32: can you provide examples of where butterfly wing patterning cannot be explained by pre-existing theories?

As noted by Reviewer 3 below, the evidence base for many of the pre-existing theories of butterfly wing patterning is actually surprisingly limited, particularly for European species. As we mention above, we think it is important to recognise that there is no reason to consider different theories to be mutually exclusive, and we expect that motion confusion is one of many selective pressures on butterfly patterning.

o Line 37: how is "evasive flight behaviour" characterised?

We have reworded this to "unpredictable" which we hope is a clearer description of what we mean here. L38.

o Line 47: "in each video frame" – I found this a bit confusing as motion can't be detected from a single frame. Later it becomes clear that comparisons are between consecutive frames, but it might be useful to clarify here.

This has been reworded to "in consecutive video frames".

o Line 48: are the four directions defined relative to direction of travel?

For the real butterfly analyses and the behavioural validation, the four directions are defined by the start and end points of the butterfly trajectories. For the simulated flights, the butterflies do not change direction, so forwards is always the direction of heading.

o Line 62: I might be getting the baseline treatment the wrong way round, but I think that the beta values should be negative rather than positive for unpatterned compared to patterned?

Thanks, we have corrected this to "patterned compared to unpatterned". L58.

o Line 88: define "var exp" – I assume "variance explained". Why are these values quite high when the R2 values are so low?

Apologies, we have realised that the way we were reporting this was not as clear as it could be, because of the way the statistics are summarised by the random forest functions we used we have now reworded "var exp" (which is indeed variance explained) as "pseudo-R^2". The other number we were reporting was in fact the MSE of the out-of-the-bag errors. The variance explained/pseudo-R^2 is calculated as 1-MSE/Var(y), and hence a small MSE corresponds to a larger pseudo-R^2.

o Line 105-7: this seems to reiterate lines 94-5 unless it is based on a different analysis?

These are indeed two separate analyses: the former are statistics based on the random forest models to identify the most important pattern characteristics for forward and sidewards confusion respectively. The latter statistics show the correlation between forward and sideways confusion.

o Line 109-110: Comparisons between flapping and gliding are justified in the supplementary information but the data needs to be referenced here.

We have briefly justified the flapping vs gliding comparison in the main text, highlighting that this isolates the effect of biological wing motion from wing pattern and wing shape. Lines 113-117.

o Line 115: In Fig 2b, bars would make more sense than lines since the exact ordering of clades depends on arbitrary choices. Can Fig 2c be labelled with whether those features affect forward or sideways confusion or both?

We have edited Figure 2b to use bars rather than lines, and we agree it is now clearer for the reader to identify local peaks.

o Line 122: selection is surely regular not random?

The butterflies were selected randomly from within the 10 degrees interval. We have reworded to make this clearer. L135.

o Line 132: is confusion truly maximised? Or would higher values be possible? Clarify that these three outcomes were under different selection regimes.

Reworded to "the genetic algorithm (GA) evolved butterfly patterns that were selected for either…" L147-148.

o Line 149: shouldn't it be over-estimate rather than under-estimate?

Thank you, corrected.

o Line 150: check the numerical scales for figure 3b.

Fixed.

o Line 158: quote the separate contributions for PC1 and PC2 so that the reader can assess their relative contribution (and/or use consistent scales on the x and y axes in fig 3b)

We have now included the separate contributions of PC1 and PC2 on L154-156.

o Line 175: define "fluid motion vision"

We have removed this phrasing from this part of the discussion, and have instead defined this concept in more depth in the methods section.

o Line 223: I'm not sure what is meant by validation here, I don't see any comparison to crab behaviour or similar in Santon et al.

We mean 'validation' in the sense of this paper being the first demonstration of using this version of the EMD model (of course, there are many previous implementations). We have reworded this sentence to try to make this clearer.

o Line 272: what was the orientation of the butterflies relative to the camera?

The butterflies were typically oriented with their dorsal surface patterning facing the camera. We have added a sentence to the methods to make this clear.

o Line 300: sentence is cut off, but this might be due to a redaction for blinding the review?

Apologies, this was an oversight, and ethical approval information has now been included. We have also included this for the new human validation experiments.

eCORN000385 and 9474868 (butterfly flight and human experiments respectively)

o Line 440: This seems to imply that each video frame is being treated as a separate data point. It would be helpful to state this explicitly.

Yes, for real flight we used each consecutive frame pair as a separate data point. We have now clarified this in the methods.

o Supplementary Figure 2: check the y axis scales. I suggest writing “Raw” in sentence case rather than all-caps, to avoid confusion with the data format mentioned elsewhere.

We have fixed the y axis scales, and have also re-titled these graphs to improve clarity.

o Supplementary Table 2: the estimates of exactly zero are a little surprising, they may be correct but just flagging for a double-check.

We have now double checked this and can confirm these values are correct (as none of the real butterflies tested had an average luminance that was that similar to the background).

o Supplementary Figure 5: “values4” typo?

Thanks, this has been fixed.

o Supplementary info lines 361-94 are missing an explanation/caption.

An explanation of these settings has now been incorporated.

Referee #1 (Remarks on code availability):

The code is well-documented and clear, and I commend the authors for the openness with which they have shared their working. I have not gone as far as trying to run the code for myself, so I can't comment on the accuracy.

Referee #2 (Remarks to the Author):

It has been suggested for a while that the patterning on some animals could combine with their motion to cause visual motion processing to deliver incorrect, and therefore misleading, motion information to the visual systems of predators. Some neat and very basic demonstrations of this were made by Zanker and colleagues a few years ago using 2-frame motion (ref 9 here, but also Zanker JM. 2019. Prey capture: becoming invisible when you move. Curr. Biol. 29(18):R875–77), this has been elaborated recently to demonstrations using realistic motions that dynamic cuttlefish patterning delivers (ref 23 here: Santon et al, 2025), but there have been no systematic studies to test this hypothesis using real animal patterns. This work does that. A recent review has highlighted the crucial gap in the literature that the work described here starts to fill (Harris et al, 2025, Annu. Rev. Vis. Sci. 11:331–57; https://doi.org/10.1146/annurev-vision-101222- 051652). The research described in this paper is the first work, to my knowledge, to do this, and thus the paper is important and highly novel: just the sort of work that Nature should be publishing. It is inter-disciplinary, working across the areas of evolutionary biology, vision science and computational modelling of vision, and thus should be of interest to a wide range of audiences.

Many thanks for the appraisal of our study's timeliness and value!

I like the work very much, but I do have some comments that I hope would encourage the authors to improve the paper. There is a huge amount of scientific work presented in this manuscript, and while I appreciate that this makes choosing illustrative figures difficult, the choices made are not always clear. I think this can be remedied easily by making some slightly different choices (e.g. the Suppl. Videos are very compelling and informative, but what they illustrate is not well described in the text).

We address these points below.

Below follow comments on specific sections and figures.

Title:

The key point being made in this paper is that interplay between the physical motion of the butterfly wings and the butterfly pattern result in incorrect visual motion processing. May I suggest that the title emphasises this inter-play. It is the effect of both motion and pattern on early motion processing that is the crucial step forward in this research. In vision science, many would describe the motions delivered to the visual by a combination of pattern and physical motion as 'errors' in motion processing, rather than 'illusory'.

We take your point that the key message of our manuscript is the interaction between butterfly motion and wing patterning and we have therefore amended the title to reflect this: 'Butterfly wing patterns in flight create powerful illusory motion cues'.

We also considered carefully whether to use the word 'illusory' in the title. We think it is appropriate because the journal is targeted towards a general audience, and while we understand that there are debates about using the term in vision science, the broadly accepted definition of illusion ('effects that act to distort the perception of the viewer', Kelley & Kelley 2014 Behavioral Ecology, 25, 450-463) should capture what we intend to express with the title for the majority of readers. We also note that reviewer #3 (below) uses the terms 'illusory motion' and 'motion illusion'.

Introduction:

The introduction is engaging, comprehensive, and covers all the bases in terms of the evolution of pattern. For me, what's missing is a paragraph describing what we know about early motion processing in predators. This would broaden the interest-base for readership of this paper. The work is inter-disciplinary, but isn't really flagged as such. The elementary motion detectors (EMD) that are used for modelling here, are based on a huge body of work from the human (and insect) vision research literature. Part of the key novelty of this work is that no-one has applied that body of work to real animal motion before in a sophisticated way. I would suggest including a short explanation for what EMD's are, and the evidence to support their existence. You have this in the Methods, but it would be great to move some of it to the Intro. In a recent paper, Santon et al (2025), describe the idea of 'generation of motion noise' as form of camouflage to explore how the motion generated by cuttlefish pattern change can potentially disrupt perceived motion. The effects described here elaborate the early motion detector (EMD) model used in that paper to give a much more detailed account of the motion of a whole range of lepidoptera species. Some of the details of the EMD motion model as elaborated in that paper, would be very usefully added here, to aid the reader in understanding more of the methods.

Thanks for this suggestion, unfortunately we are very restricted by space in the main body of the manuscript. However, we have now added a brief introductory sentence for EMDs as suggested.

Methods/Results

Motion confusion and Figure 1:

The analysis of motion is performed along the cardinal directions. This seems a sensible choice, keeping the motion calculations down to a manageable size. It was

not explained how forward, backward, left and right were defined. Was this with respect to the video frame, or the direction of motion of the animal? If the latter, how was the ground-truth motion direction and speed calculated? There is a clue in the figure legend of Suppl Fig 1, where the authors say “Note, as the butterfly does not fly in a straight line forwards and backwards, energy will not always correspond with the heading of the butterfly.” This suggests that the motion directions are not referenced to the instantaneous direction of motion of the butterfly (its heading). This is an important technical point which could be explained in more detail. Given that the authors later do some clever modelling of flight dynamics (supplementary video 2 – Butterfly blender rendering), these details could be nailed down more carefully.

For the real butterfly analyses and the behavioural validation, the four directions are defined by the start and end points of the butterfly trajectories. For the simulated flights, the butterflies do not change direction, so forwards is always the direction of heading.

Thank you for picking up the wording about speed. This was imprecise terminology on our part (EMD models do not estimate speed) and we have now corrected this throughout the manuscript.

The results shown in Figure 1 clearly show the significant differences between patterned and non-patterned stimuli, but I found the data of Suppl Fig 1 much more compelling. You might want to consider promoting Supple Fig 1 to part of a full figure.

Again, we are unfortunately very limited on space and we have had to prioritise visualisations that encompass the full range of the data compared to Supplementary Figure 1, where we show data for one example only (and it would be difficult to translate into a version that could accurately represent all butterflies).

However, some motion errors (or as the authors define it, motion confusion) occur for all motions detected by EMD’s. I also wondered what the EMD model would deliver for a much simpler moving stimulus? It is important for the audience to realise that errors in motion direction and speed will likely occur for any object moving. For example, if the animal body was emulated without the wings (eg. one could use rectangle to illustrate the approximate location of the butterfly body, estimated frame-by-frame).

As part of our behavioural validation where we used computer generated butterflies along simulated flightpaths, we also rendered a white triangle of the same width as our butterflies along these flightpaths. This acts as a ground-truth for the EMD of the path movement without interference from the wing behaviour of the butterflies. The mean forwards-confusion measure for our butterflies was -0.32 ± 0.7 (standard deviation), while a triangle had a mean of -0.95 ± 0.2. A forwards-confusion value of

-1 is purely forwards with no backwards motion. Similarly sideways confusion for the butterflies was 0.02 ± 0.2 and was only -0.46 ± 0.1 for the triangle. These differences, as with differences observed for gliding see Supp Material 2.iiii, are due to the absence of backwards and sideways movements generated by the wingbeats.

Analysis across species and Figure 2:

I really liked the analysis across 397 species, and 757 morphotypes. This represents careful and diligent work. Figure 2a and b really need to be bigger. It could be just the pdf, but the resolution of the image is too low to illustrate the butterfly patterning well.

We have reworked Figure 2 to try to increase the size of the subplots. We have a high resolution SVG version of the plot which will be available with the final manuscript.

What is a forest plot as used in this context (figure 2c)? I'm familiar with such plots used to illustrate data such as log-odds ratios in meta-analyses. I don't think the authors are using the term in the same way here. Could the authors please explain what they are plotting in figure 2c? There are some details of 'Random Forest' modelling given in the Methods, but for the uninitiated (including me) there is not a clear explanation of what is plotted, nor why.

We have rearranged Figure 2 to encompass data from our behavioural validation experiment, so Figure 2c has been removed. However, in brief, Supplementary Figure 10 shows the SHAP values, which tell you how much each feature in the dataset contributes to the predictions of your model. We then visualise these in Supplementary Figures 12 and 13 which shows the wing images and the metrics to give the reader visual demonstration of how changes in the feature value map to butterfly appearance.

Evolving butterflies using genetic algorithms and figure 3:

Again, great work. The power of this work is that the genetic algorithms used do indeed 'evolve' the kinds of patterns that have been suggested to be effective at disrupting the overall motion signals. For the first time, to my knowledge, the results here show that they do. That the evolved patterns are similar to those of real butterflies is also a really important result.

Thanks!

I found the interaction with overall wing shape particularly interesting. The method is very nicely illustrated in Suppl. Video 3. However, the text in this part of the Results section is difficult to follow without a careful reading of the Methods section. I

suggest the authors take a look at this and work towards a simple explanation in this section, summarising what was done, and what was measured, so that is clear what each part of the figure is illustrating.

For example, it would be useful to describe in more detail what the PCA hulls graphs illustrates (Figure 3b). The data of figure 3C are also important, demonstrating how close some natural butterfly patterns are to these genetically evolved optimal patterns. These details are elaborated nicely in the Methods section, but some could really do with being promoted to the main text so that the crucial points can be appreciated by a non-technical audience.

We have reworded the caption for Figure 3 to improve clarity.

Typos and other small points:

Line 300: sentence about approval is not finished. Is this where any ethical approval should be mentioned?

Apologies, this was an oversight and has now been included.

Line 501: Is the code for the elaboration of the EMD model available? This would be a very useful addition to open-source code that would allow other groups to apply this form of modelling to other natural motions, and indeed to other contexts including the design of human environments (e.g. driving, trips hazards in urban spaces, etc).

We have made a short EMD script that is now available as part of our supplementary material. Please note that this script will take .avi files or image stacks but all visual-system-specific pre-processing must be done before using this script.

Referee #2 (Remarks on code availability):

I have looked at the code materials, not reviewed the code in detail, nor tried to run it. I hope that other technical referees with more current coding skills will be willing to do that.

I may have missed it but, Is the code for the elaboration of the EMD model available as a stand-alone? I think it sits in the ImageJ code folder, but that seems to include all sorts of material that might come from other projects too. I didn't see any README files there. This would be a very useful addition to open-source code that

would allow other groups to apply this form of modelling to other natural motions, and indeed to other contexts including the design of human environments (e.g. driving, trips hazards in urban spaces, etc).

See above.

Referee #3 (Remarks to the Author):

The paper 'Butterfly wing patterns create powerful illusory motion cues' presents three strands of investigation into the hypothesis that butterfly wing patterns can function in motion camouflage. Misleading predators about the true direction or speed of movement could then reduce the probability of successful attack. This is what many in the field call dazzle camouflage, thought the authors have chosen not use this term.

Dazzle camouflage/pattern based motion illusions have been investigated for several decades with a complex and not yet especially solid evidence basis (meta-analysis in Ref#5, also reviewed in Tan et al. 2024 https://doi.org/10.1111/brv.13044 which would be a useful citation). Lab experiments (generally humans or birds catching artificial prey on monitors) have had low replicability, and there is currently little evidence that 1.) this class of camouflage mechanism can reduce predation in natural conditions and 2.) that it is utilised by any animals, though it has been suggested for several species and groups of species. To my knowledge, butterfly wing patterns have not yet been suggested as functioning in motion confusion, so the proposal that is explored here is novel. I comment on each of the three elements in turn:

Many thanks for the assessment of our work's novelty. We have added Tan et al. 2024 as suggested.

1.) Analysis of high-speed videography of several butterfly species using elementary motion detectors suggests that illusory motion signals are produced.

EMDs are simple local motion detectors. Motion of contrasting elements either within a wing, or between a wing and a background, on a different vector to the global motion (such as a vertical wing stripe on a flapping wing) will generate an 'illusory' response. Dealing with noise (variability) in local motion signals is a challenge for all visual systems when trying to estimate global motion, and they are adapted to do so. What matters in a predator-prey interaction is how noise affects the global motion percept – when do errors occur? This depends on how local motion signals from different areas of the object are combined. Intuitively the accuracy of this should increase as the local motion signals are more consistent, which is the argument made here (L51) and in many global motion models, but this may not be the case – Green & Pratte (2022 10.1037/xhp0000971), for example, provide evidence that

global motion perception either happens with a high degree of precision, or not at all. The global motion model used (Michelson contrast between energy in different directions) in this study is very basic and should be properly justified as having a basis in biological reality as it is key to conclusions.

Thank you for drawing our attention to this interesting paper. If we have understood it correctly, it suggests that "motion perception is discrete when it is accomplished by segmenting local motion energy into signal and noise, but is continuous when local motion directions are pooled to derive an average direction". The latter seems entirely consistent with our approach; the former seems to refer to transparent motion perception, which is less relevant to our stimuli. Michelson contrast has been commonly used in motion modelling (e.g. https://doi.org/10.1016/S0042-6989(99)00147-9).

Better, alternative global motion perception models could be evaluated. The gold standard would be experimental evidence that the direction and strength of local motion signals correspond to predator targeting errors. I appreciate that this is probably another project, but without it, the conclusions of the current work are inevitably less convincing.

We completely agree that behavioural validation is the 'gold standard', and as such, we have now completed a validation experiment. See further details above (both in response to reviewer 1 and at the beginning of our full response).

2. Wing patterns of European butterflies can have high levels of motion confusion. This is a very interesting analysis that investigates EMD outputs of a model receiver viewing European butterfly wing patterns on a 3D animated butterfly. It then identifies what pattern features are associated with modelled illusory movement, and finally examines phylogenetic patterns in the degree of motion confusion. It concludes that illusory local motion signals are common and widespread in the species studied, and that motion camouflage could therefore be the function associated with otherwise unexplained variation in wing pattern. The methods used in this analysis are excellent, as are the figure and supplementary movie, but I found the premise on which the conclusions are built more problematic.

The paper argues that colour pattern function in European butterflies is not generally well explained by current hypotheses. This implicitly supports the decision to not evaluate the motion confusion hypothesis against alternative hypotheses for wing colour pattern function. However, I would argue that this is not an accurate characterisation of current understanding. Camouflage, thermoregulation, warning, deimatic and sexual signal functions have all been demonstrated in different species of European butterfly. For other groups of species, the evidence is very limited and uncertain (e.g. Pieridae Lyytinen et al 1999 https://doi.org/10.1023/A:1011081800202) or absent entirely. Thus, I would argue

that uncertain function for many species has more to do with a lack of investigation rather than research ruling out hypotheses leaving the function enigmatic after thorough research.

We agree that the evidence base remains limited for many putative functions of butterfly patterning in European species. We have reworded this section to try to make it clearer that our point is that the function of these high contrast dorsal wing patterns is still unknown. We also reiterate the point discussed earlier in our response to Reviewer 1, that proposed hypotheses do not need to be mutually exclusive. Comparisons between these hypotheses would therefore require experimental manipulation across multiple proximate mechanisms, together with ultimate effects, across hundreds of species. Such analyses are simply not feasible.

Not properly considering alternative functional explanations may lead to erroneous conclusions. Some functions have similar form requirements while others predict different phenotypes. For example, there is evidence that wing pattern in the European swallowtail Papilio machaon, a species with high motion confusion score, is part of a deimatic signal (Olofsson et al 2021 https://doi.org/10.1371/journal.pone.0047092), as might be the peacock butterfly Inachis io (Vallin et al. 2005). The white band on the white admiral, another species with a high motion confusion score, is thought to be a disruptive camouflage marking (Stobbe & Schaefer 2008). Disruptive camouflage, deimatic, aposematic and sexual signalling functions are all supported by high contrast markings, so as pointed out (L171), the evidence presented is equivocal whether motion confusion, disruptive camouflage, a signalling function, or multiple functions are in play. The solution to this problem would be testing alternate comparative hypotheses made by the different hypothesised functions. In which ecological conditions would high motion confusion be expected to evolve? In which would sexual signals be expected to evolve?

Olofsson et al (2021) tested only swallowtail butterflies, and therefore had no control for wing patterning: they themselves say point out that their experiments cannot disentangle "whether the conspicuous coloration is essential or whether the size and sudden opening of the wings explains the strong evasive responses of the birds". We would therefore argue this evidence does not conclusively demonstrate that patterning is a component of the deimatic signal.

Stobbe and Schaefer (2008) demonstrate that contrasting patterns do not necessarily increase predation rates, although it is not clear from their results whether these patterns truly need to be disruptive (as they do not test a high contrast, non-disruptive control), although this has been shown in other species e.g. Seymoure & Aiello 2015, Journal of Evolutionary Biology, 28, 1618-1624.

We do not dispute that high contrast markings are likely to have multiple functions in many cases, and we agree that it will be very interesting for future work to disentangle these more fully. However, we believe this to be beyond the scope of the current manuscript, where we aim only to fully document (for the first time) that motion confusion is a plausible explanation for these patterns.

Understanding more about the ecological conditions under which different signals evolve would also be a very interesting direction for future research. In the current manuscript, we took the decision to not include ecological variables, as the information available for European butterflies is very patchy.

3. Artificial butterflies evolved for motion confusion resemble real butterflies.

This is a solid analysis using a very nice approach that I find quite convincing, but with the same issue. It would be greatly strengthened if it was able to show that motion confusion evolved artificial butterflies were more similar to European butterflies than warning signal or disruptive camouflage evolved artificial butterflies etc. I would suggest that comparisons are only made between species that have high scores on a metric, as species with low scores are unlikely to be using the potential function.

We agree that comparisons between motion confusion, warning coloration and disruptive camouflage are a clear area for future research. However, this remains impractical for a genetic algorithm approach at the present time. For motion confusion, we are fortunate to have biologically-relevant metrics that we can select for (e.g. forward confusion, sideways confusion). This is not the case for aposematism or disruptive camouflage. For example, while we could select for high GabRat scores (Troscianko et. al 2016, BMC Evolutionary Biology https://link.springer.com/article/10.1186/s12862-016-0854-2) we already know (from our own pilot work) that this will generate highly unrealistic stripes perpendicular to the edges. Using humans as model predators (e.g. to select for targets that are found quickly or slowly) is theoretically possible as an alternative approach, but is unrealistically labour intensive for the current manuscript (e.g. recent work in our group has required 1 year plus of data collection for these types of genetic algorithm experiments with humans, https://doi.org/10.1101/2025.03.01.640995) and we think this really constitutes a separate research question.

I would also recommend toning down the ‘independence’ of each investigation (L9 and elsewhere). They all rely on the same motion detection models, and they use the same set of parameters (as pointed out on L189). I think this is fine – there are higher priority additional analyses in my view (as suggested above) than sensitivity analyses over different flight behaviours and viewing directions etc. – there is no strong need to say they are independent lines.

We have removed the word independent at L9. We have also now added another line of evidence (behavioural validation) in support of our hypothesis.

In summary, I think this is a novel and exciting idea, and therefore of course somewhat speculative. Applying three approaches to investigate is a strength of the paper, and the methodologies of each approach are all excellent. However, for a paper of the highest quality, I think the evidence provided by each approach could and should be more convincing in support of the conclusion.

We hope that the additional validation work ties these results together convincingly, although the reviewer doesn't specify what evidence would be more convincing.

Referee #3 (Remarks on code availability):

I was unable to spend lots of time reviewing the code, but I was able to easily understand and access all of the data and analyses that I tried. The organisation and commenting seemed exemplary.

Thank you!

Referee #4 (Remarks to the Author):

Camouflage has a long history as one of the primary anti-predator strategies, yet it is well known that motion breaks camouflage. However, the mechanisms by which prey avoid attacks once they start moving remain poorly understood, and empirical evidence is limited. This study makes a significant step toward addressing this problem. The authors investigate how butterfly wing patterns interact with flight dynamics to generate illusory motion cues that may mislead predators. To do so, they combine high-speed recordings of butterfly take-offs with biologically inspired motion modelling, comparative analyses across hundreds of species, and in silico evolutionary simulations. This is a remarkably clever and creative piece of work. The multipronged approach is impressive, and the idea that color patterns may actively exploit limitations in predator motion perception has the potential to shift how we think about pattern–motion interactions in nature.

Thanks for this appraisal of the novelty and value of our study.

However, several conceptual, methodological, and interpretational issues (detailed below) make it difficult to fully evaluate the strength of the evidence in its current form. Clarifying how the modelling results translate to biologically meaningful effects, better defining the mechanism relative to existing concepts such as motion dazzle, and explaining the rationale behind key analytical decisions would greatly strengthen

the manuscript. As it stands, I am unable to recommend publication at this stage, but I believe the work has the potential to make a substantial contribution after revision.

We believe this concern is now addressed by our extensive validation work (discussed above), and this reviewer makes valuable suggestions that we respond to in specific detail below.

General comments
1. The effects described in the manuscript are demonstrated primarily through modelling, but it is unclear how well these results translate to real predator–prey interactions. I am fully aware of the practical challenges involved in testing these ideas with real predators. However, previous studies on motion-based antipredator strategies have routinely used human observers as a first step. Incorporating a similar 'simulated predator experiments with humans' at least would greatly strengthen the biological interpretation of the results. Alternatively, the authors could at least translate their modelling outputs into more interpretable quantities (e.g., how much the observed forward–sideways confusion would translate into shifts in perceived speed or direction) so readers can better assess the magnitude and ecological relevance of the effects. As it stands, without empirical validation through experiments, the inferences remain difficult to evaluate. For example, while the modelling shows that certain patterns produce forward-confusion, it is unclear whether this level of confusion is sufficient to reduce attack success in any meaningful way.

We completely agree that while challenging, behavioural validation of this work is vital for strengthening the biological interpretation of this work. To this end, we have now completed behavioural validation of our results using human 'predators' trying to catch moving butterflies on a touch screen. Importantly, our methods incorporate temporally aliased videos on high speed monitors, avoiding motion artefacts (such as the 'wagon wheel' effect) that have not been properly controlled for in previous studies. Our results match what would be predicted from our EMD models: butterflies that are predicted to have a high level of forward confusion have a more elongated kernel of click density, meaning that participants were more likely to click behind the butterfly, and our statistical modelling confirms that the forward confusion metric is a significant predictor of how far back a participant's click will be. Further details are available in the main manuscript (Figure 2c and lines 119-126), along with detailed descriptions of the experiment in the Supplementary Material (Section 4).

2. The current modeling results show that patterned wings generate stronger illusory motion cues than an unpatterned treatment, but it does not demonstrate that they are more different than camouflage. This is an important issue because motion usually breaks camouflage, and patterned wings could represent an alternative defence once the animal is moving. The authors do not address this point directly, and the backgrounds used in the modelling are always contrasting with the

butterflies. Yet this question is central to understanding the broader relevance of the mechanism. Discussing this limitation, or clarifying that the study is not making claims about effectiveness relative to crypsis, would make readers more aware of this aspect.

Please see our response to Reviewer 1 (point 5, emphasising that a moving animal cannot camouflage against a natural background). Additionally, initial pilot work with different backgrounds: in brief, motion confusion effects are relativelys table across different background conditions and dorsal viewing angles.

We have not made claims about motion confusion relative to crypsis because it is not clear to us that these mechanisms must be in opposition: signalling is distance-dependent, so it is completely possible (indeed likely according to theories of complementary coloration) that a butterfly with high contrast patterning is cryptic when viewed from far away.

3. Throughout the manuscript, the proposed mechanism (‘motion confusion’) is presented as if it is fundamentally different from motion dazzle, even described as a ‘hitherto unknown’ visual defence strategy. However, the effects described here, including changes in perceived speed and direction produced by high-contrast patterning, closely resemble classic motion-dazzle mechanisms demonstrated in other animals. It would be helpful for the authors to clarify whether their findings demonstrate a genuinely distinct mechanism, and if so, how it differs from existing explanations. Alternatively, do the results primarily offer empirical support for longstanding dazzle hypotheses when viewed through the lens of butterfly flight dynamics and EMD analyses? Clarifying this would help readers understand the novelty and avoid conceptual ambiguity.

We wished to avoid some of the conceptual confusions surrounding ‘motion dazzle’ as this term has been used to describe both a type of patterning and to describe a presumed function. However, we agree that the suggested mechanistic definition of dazzle used in the most recent work on this question (e.g. Scott-Samuel et. al 2023, Biological Journal of the Linnean Society, 140, 485-503 https://academic.oup.com/biolinnean/article/140/4/485/7252242) does map well onto our own ‘motion confusion’ terminology, and we agree that we are indeed offering support for longstanding dazzle hypotheses. Our behavioural validation work also supports our link to higher ‘motion confusion’ causing both consistent misdirection and higher variance in attack locations. We have clarified this at L54-54, and have removed the “hitherto unknown” wording from the abstract.

4. Finally, several key analytical choices are not fully justified (e.g., viewing angle), and clearer explanations of why particular decisions were made would strengthen the manuscript (see specific comments).

As discussed above, we have comments from reviewers that suggest exploring different model parameters such as viewing distance, viewing angle, background, flight dynamics, etc. While many of these questions may add interesting nuance to our findings, the additional work in terms of computing power and time (particularly when considering combinations of variables) would not be feasible given the already ambitious level of processing undertaken. Although regarding viewing angle in particular, we assumed that on take-off (when butterflies are most vulnerable) predators would most likely have a dorsal view (which aligns with the fact that butterfly dorsal wing coloration is often much more high contrasting than their often cryptic ventral wing surfaces).

Specific comments

1. Line 17 & 20: Highly relevant papers to cite.
Ioannou, C.C. and Krause, J., 2009. Interactions between background matching and motion during visual detection can explain why cryptic animals keep still. Biology Letters, 5(2), pp.191-193.
Tan, M., Zhang, S., Stevens, M., Li, D. and Tan, E.J., 2024. Antipredator defences in motion: animals reduce predation risks by concealing or misleading motion signals. Biological Reviews, 99(3), pp.778-796.

Thank you for these suggested references: we have now incorporated them into the manuscript at L17 and L26 respectively.

2. Line 37-38: This sentence is difficult to follow. It is not clear whether the authors are suggesting that species with striped wing patterns typically also possess eyespots and evasive behaviours, or whether eyespots are intended as an independent example of how wing patterns and flight behaviour might interact to affect motion detection. Note that deflection via eyespots does not require any movement component. So, this is misleading. Moreover, the cited study by De Bona et al. 2015 (Reference 17) addresses the intimidation (predator mimicry) hypothesis, not deflection. The paper below might be relevant for deflection.
Prudic, K.L., Stoehr, A.M., Wasik, B.R. and Monteiro, A., 2015. Eyespots deflect predator attack increasing fitness and promoting the evolution of phenotypic plasticity. Proceedings of the Royal Society B: Biological Sciences, 282(1798), p.20141531.

Thank you for this reference, we have now incorporated this in place of the De Bona paper. We have also clarified our point about eyespots at L39, where we intended to suggest that motion may enhance their deflective roles.

3. Line 40: I do not think this statement is accurate. Most avian predators are diurnal, so nocturnal moths are unlikely to be widely targeted by birds while in flight. Predation risk from birds is largely associated with daytime resting, where moths rely on crypsis rather than evasive flight.

While we fully agree that many birds are diurnal and birds do often attack resting butterflies and moths, there are a large number of species that target nocturnal moths in flight e.g. nightjars and allies (Caprimulgiformes); Papuan swiftlets; various nocturnal owls that take large insects in flight; potoos, oilbirds and allies (Nyctibiiformes).

4. Line 27-42: The final section of the introduction is not very compelling. It mixes several distinct mechanisms (e.g., deflection, intimidation, evasive behaviour) that do not necessarily affect motion misperception. As a result, it is difficult to see how these examples build toward the proposed hypothesis. I recommend rewriting this section so that the proposed mechanism is stated more clearly and the narrative focuses directly on how wing patterns and flight dynamics are expected to create misleading motion cues. For instance, would the authors consider their proposed mechanism to be distinct from, or a specific form of, motion dazzle? Clarifying this would help readers understand whether the idea is potentially novel or how it fits within existing mechanisms.

(See response to reviewer #4, General comment 3 above). 'Dazzle' has been defined very broadly (Scott-Samuel et al 2023 define it as 'coloration that interferes with the interception of a moving object, due to perceptual distortions of target speed, trajectory and/or range'). We have tried to define more clearly throughout the introduction that we consider motion confusion to be synonymous with this mechanistic definition of dazzle, which we hope makes this section more understandable.

5. Line 52: The term 'deflection' is used somewhat loosely here. It can refer to misdirection toward less vulnerable body parts (e.g., wing margins; sensu stricto) or to mechanisms that misdirect attacks away from the animal entirely (which I assume is what the authors intend). Please clarify which meaning is meant. If the latter, it may be clearer to refer to this as 'misdirected attacks'. It may be worthwhile to define this in the last section of the introduction.

We think both types of deflection are relevant in this case, as both would increase a prey's chance of survival. We have reworded to 'misdirect' but are happy to take the reviewer's advice if they have suggestions for better terminology for this intended meaning.

6. Line 57: The rationale for using only unpatterned treatments is not clear. It may be more appropriate to include patterned prey with the same overall contrast but without stripes (e.g., blotches or random patterns)? This would provide a more appropriate comparison.

We think this comparison is better made in our genetic algorithm analyses, where we generated and tested 41,248 unique wing phenotypes, exploring a vast phenotypic pattern space. This includes butterflies evolved under random selection that were compared to those evolved under selection for different types of motion confusion. For the real butterflies, it is not clear to us how best to generate patterned but non-striped comparisons without introducing quite arbitrary decisions about placement of markings.

7. Line 64-65: I may have missed it, but it is not clear that the results actually show 'estimates of speed,' as the figures only present forward and sideways confusion. Is this based on forwards-energy? Also, does it imply that a contrasting but unpatterned prey could be equally effective in misleading motion?

Thank you for picking up the wording about speed. This was imprecise terminology on our part (EMD models do not estimate speed) and we have now corrected this.

8. Line 109: It is not clear where these results are presented. Could the authors point to the relevant figure or supplementary material with numbers?

We have now clarified our reference to flapping and gliding (see responses to Reviewer 1)

9. Line 133: Please move this definition of forward energy to the section above where the term is already introduced.

Forwards energy is first defined in the 'Motion confusion' section (L47-54) and is included here as a reminder for the reader.

10. Line 146: P-value for random selection missing.

We have now added the missing P values from the table in the Supp material section 3.

11. Line 149: I may have misunderstood, but this seems contradictory to the earlier interpretation where the authors suggest that unpatterned white prey should have a higher estimated speed (Line 65). Should this instead refer to overestimated forward speed?

Thank you, Reviewer 1 also spotted this issue, and we have now corrected it.

12. Line 153: The x-axis label for panel c and the corresponding legend might be confusing, as the treatment 'Natural' is also included. Please clarify what 'Natural' represents in the legend.

We have re-clarified in the legend that Natural refers to the butterflies from Collins Guide to Butterflies.

13. Line 164 & 187-189: It is not intuitive why one would expect illusory motion to result in deflection of attack. Is this the same as what has been proposed for lizards (Murali et al., 2016)? Could the authors expand? Alternatively, I suggest rewriting this as 'misdirected attacks' rather than classical deflection (i.e., 'attacks redirected to less vulnerable parts'). See my comment above on this.
Murali, G. and Kodandaramaiah, U., 2016. Deceived by stripes: conspicuous patterning on vital anterior body parts can redirect predatory strikes to expendable posterior organs. Royal Society Open Science, 3(6), p.160057.
Murali, G., Merilaita, S. and Kodandaramaiah, U., 2018. Grab my tail: evolution of dazzle stripes and colourful tails in lizards. Journal of Evolutionary Biology, 31(11), pp.1675-1688.

We have reworded to 'misdirect', but as discussed above, we believe that these types of patterns are likely to cause both 'misses' and attacks redirected to less vulnerable parts e.g. in the case of swallowtails, the forward confusion effects direct attacks backwards, making them more likely to miss or at least only hit the relatively less important tails.

14. Line 266 onwards: It would be helpful to clarify why recordings were restricted to take-offs. Mid-flight wingbeat patterns can differ substantially. Please provide justification for limiting the recordings only to take-offs.

We recorded take-offs because most predator attacks begin on stationary butterflies (now stated on L296). Additionally, it is very difficult to do controlled recordings of free flying butterflies!

15. Line 267: It would be helpful if the authors could include the rationale behind selecting these five species for this analysis. Do these species represent the major colour-pattern morphotypes observed, and if so, how was this determined?

These five species were available at the earlier stage in the project when we were carrying out recordings: they should be considered as a 'proof of principle'. We used the Collins data to ensure we were covering the full range of colour-pattern morphotypes observed in nature.

16. Line 274: For how long?

Durations of videos are now provided on L307-308.

17. Line 293: Please include the number of individuals per species. Also, the actual number of video trials used in the analysis (i.e., to generate Figure 1) per species. I presume they are the same across treatments.

This information is now included at L292-293.

18. Line 311: Why were only dorsal patterns analysed and not both dorsal and ventral? Many species vary in ventral coloration and appearance change across viewing angles, so please justify this.

Butterflies typically close their wings when not flying or displaying, showing only their (often) cryptic ventral surfaces. On flight, the high-contrast dorsal patterns (that our hypotheses set out to explain) become visible. It therefore makes most sense to focus on dorsal patterning visible on take-off (as justified above). Additionally, ventral patterning is less well documented: for example, we would not have been able to get ventral patterning records for all species in the Collins book.

19. Line 326: The 3D flight rendering appears to be based on a single species, whereas the colour-pattern analyses encompass the full dataset of over 750 morphotypes. Because different species exhibit distinct flight dynamics, it is unclear whether a rendering based on one species can adequately represent the broader diversity being analysed. The authors should clarify the rationale for restricting the 3D component to a single species and discuss how this affects the generality of the conclusions drawn from the modelling. At the very least, why not use the flight dynamics of all seven morphotypes that were recorded, assuming these represent colour-pattern–related differences in flight behaviour?

The subsample of species we have videos for are not fully representative of the range of flight behaviours, and as discussed further above (see response to reviewer #1 comment 4), adding complexity in the form of a larger number of flight paths would increase processing times beyond what is reasonable, especially when considering the combinations with other factors. We do cover gliding behaviour further in the supplementary material which covers a large amount of butterfly flight variation, and adjust wingbeat frequency based on the butterfly's wing area/body size. We are therefore confident our analyses cover a good proportion of butterfly flight dynamics.

20. Line 346: Please add justification for why the analysis was restricted only to one viewing angle.

We have added a sentence to justify this: largely, we used one viewing angle for tractability. L376-379.

21. Line 393: Please provide more detail on the key parameters used in the simulated evolution analyses (e.g., number of prey, population size, mutation rates). Please clarify how these parameter values were chosen, and how sensitive the results are to the specific values used. If these details are already included in the Supplementary Information, please link to the specific table or section.

This information is now available in the supplementary materials (Section 3.ii).

22. Line 471: What were the predictor and response variables in this analysis, and which random effects were included?

Correct to LM as random effects weren't used for the in silico butterflies as each population was reduced to 1 individual.

23. Line 478: Could the authors briefly mention what these metrics represent? Are they the same as those described in the section on 'butterfly wing features'?

Yes, these are the same as those described in the section on butterfly wing features. We have now clarified this.

24. General comment: Please specify the exact supplementary table or figure numbers when referring to them, as there are many, and the current references are unclear (e.g., Line 146).

We have reorganised the Supplementary Materials to improve clarity and ease of reference (each section is now numbered e.g. 1.ii).

Referee #4 (Remarks on code availability):

I reviewed the code provided by the authors, and the repository is generally well organized. The README file offers clear instructions for installation and execution. However, I was unable to install and run the main EMD module, which appears to be missing. I spent considerable time locating it but was not successful. As a result, I could not reproduce the analyses that rely on this component. Apart from this issue, the remaining scripts function as described.

We have made a short EMD script that is now available as part of our supplementary material. Please note that this script will take .avi files and image stacks but all relevant pre-processing must be done before using this script.

Referee #5 (Remarks to the Author):

This work offers an original and convincing interpretation of how butterfly wing patterns and movements might serve to confuse predators. The methods are

appropriate, and the main conclusions are convincing. The findings are both of broad significance and (at least to me) not a priori obvious. Overall, this is the most significant recent work on how defensive coloration works to conceal and confuse, and complements the wider literature on the evolution, development and function of butterfly wing patterns.

Thanks for the positive assessment of our study's novelty and convincing findings.

The key finding - expressed by the 'forwards confusion' measure - is that the relatively low beat frequency of butterfly wings combined with their pattern produces a forward motion signal that differs from that produced by a gliding butterfly (simple translation). By comparison the sideways confusion, forwards (motion) energy parameters (Fig 3a), and apparent speed need more explanation.

i) A gliding butterfly with stripes produces 'sideways confusion' (line. 109) presumably due to the 'aperture effect' (Adelson & Movshon. Nature 300:523, 1982). Sideways energy (l. 248) sums left and right energy, but for a bilaterally symmetrical pattern would directional signals cancel out, so the butterfly would not appear to deviate from its actual path (c.f. l. 53)?

Indeed, which is why we couldn't use Michelson contrast as left versus right for quantifying sideways motion confusion from dorsally rendered flight. Nevertheless, our validation experiment and analysis of real butterfly flight show that sideways motion confusion (as we have analysed it) does produce meaningful effects, albeit much less intuitive than those of forwards confusion (in line with the reviewer's point).

The ms. could be more clear about the relationship between 'motion dazzle' (e.g. Ref 5) produced by simple translation (e.g. gliding) and the effects of wing movements.

See response to reviewer #2 above regarding EMD output for a simple stimulus.

More generally, elementary motion detectors do not directly measure velocity, so the relevance of findings that forwards (motion) energy is greatest for a unform white butterfly (Fig. 3a), and inconsistently (?) that higher contrast [markings] resulted in higher estimates of speed (line 64) need more justification and explanation to support the main argument.

Apologies, we didn't mean to imply that we measured speed as EMDs can't do this. We have now corrected this sentence.

Fig 1 is quite difficult to understand without reference to the text (e.g. lines 246-250). Can more explanation be included in the legend, or even 1 added to the confusion parameter so that 0 (rather that -1) corresponds to no effect of wing movement.

We have updated Figure 1 and its caption to aid understanding of forward and sideways confusion. Because the metrics are based on Michelson contrast, we want to keep them on the same scale to aid readers who are familiar with this approach.

Editor comments:

The revised manuscript, "Butterfly wing patterns in flight create powerful illusory motion cues", has now been seen by four of our original five referees. I am sorry this has taken so long. The reason was that Referee 1 (R1) failed to deliver a report, or even respond to our requests. Therefore I asked the remaining referees if they'd examine the comments raised by R1 on the earlier version of the ms, and your responses to them. R3, R4 and R5 did so and these comments are appended to the end of this letter, after the referees' own comments.

The manuscript is clearly getting there, but we feel that at least one more revision is required so that you can accommodate the referees' remaining points.

As the work now includes human research participants, you will need to be clear about who these people were and what they did together with any relevant statements about ethics, consent and so on. You will need to explain your sampling strategy and how it could bias the results, etc. In summary, you will need to fill out the "human" part of the reporting summary.

> [Response] Thank you for your kind words and for your efforts to locate Referee 1. We have now included an Ethics Statement in the Methods section, moving the ethics for our butterfly recording into a single section. We have now moved the majority of our method descriptions for our “Butterfly Game” to the main text. We have also provided a completed reporting summary, although we have provided three versions as we weren’t sure if the human experiment needed to be split into a different document. We have also edited the manuscript in accordance with this second round of reviewer responses.

Referees' comments:

Referee #2 (Remarks to the Author):
This is a much-improved paper compared with the version I originally commented on.

> [Response] Thank you!

In particular, the behavioural experiments using humans to test the EMD model predictions is a valuable addition, making the interpretation of the modelling results much more robust (though see my last point at bottom of this paragraph). The inclusion of the new figure 2c is a great way to illustrate the human behavioural data in terms of error in attack location, but it would be more valuable if the authors referred to it when they describe the human behavioural experiment (lines 164-171).

> [Response] We have now done this (reference to Figure 2C included at L126).

Further, there is no additional section in the Methods describing the human experiments. These are really important, as the authors highlight in the Response to Reviewers, so please add some details to the Methods, even if that is simply a sentence pointing to the details in the Supplementary Materials.

> [Response] Thanks for this point; we have now included the majority of the details of the human experiments in the Methods section, leaving the figures and the additional results within the supplementary materials.

One point of clarification here: the authors state that feedback was given to participants during the training tutorial, but not whether it was given in the main experiment (I assume not, but this needs to be made explicit).

> [Response] We have now clarified this. Feedback was only given during the training trials but not the experiment: L599-602.

Finally, the authors state “Contrary to our predictions, sideways-confusion actually decreased the distance perpendicular to the butterfly (tightening attacks) and caused attacks to be further forward.” That is interesting. It

does potentially undermine the comparison between behavioural and modelling data. Some interpretation of this issue would be valuable here.

[Response] Given the tight correlation between forwards and sideways confusion in real butterfly wings (fig 2a), and evidence from the GA-based experiment that selection in real butterflies acts primarily on forwards (rather than sideways) confusion (Fig 3b), we would actually predict this observed effect in our human trials. As such, we have adjusted the description to reflect this. We now state:

Based on our simulated flight data, this may be expected as a byproduct of strong selection for forwards confusion, given their tight correlation (again see fig 2a) and the closer resemblance of fowards-confusion selected butterflies to natural (fig 3c).

The Introduction is tighter now, and will likely fulfil most of the reviewer requirements suggested in response to the previous version. Yet it still offers an interesting read to those who are less expert.

The additions showing motion confusion directions in Figure 1 aided understand of the 2 dimensions of confusion, making this figure much easier to digest.

I liked this work when I first read the paper, I think it provides a much stronger contribution now and I recommend acceptance in Nature.

[Response] We thank you for your kind words and appreciate your approval of our work.

Some small points:
Line 131: I'm still non the wiser about what random forest models are. For the general reader, it would be useful to add a reference here giving an example of how they work in more detail. It's in the Methods, but good to add to main text too.

[Response] We have now added a reference and some further explanation of random forest models at L92-94 as requested.

Line 361: end of sentence missing.

[Response] We apologise for this error, the sentence has now been fixed.

L373-375 "A custom-written ImageJ + Python script was then used to automatically replace the images used for the butterfly's body, hindwings, and forewings with those of a selected butterfly, as well as to adjust the wing beat frequency and render the animation."

Referee #3 (Remarks to the Author):

The behavioral experiments complete the study, which provides a remarkable integration of convergent strands of investigation. The evidence for the proposal is now convincing. It changes current understanding and will inspire work for years to come. I congratulate the authors.

The many other changes made by authors have substantially improved the paper, and where suggestions were not implemented, the response letter is persuasive. This was generally because they are outside the scope of what is feasible for this study, which on reflection I agree with. I expect reviewers got excited about the idea and in part identified avenues for future research - I think this can be taken as an indicator of the influence the paper will have.

[Response] We thank the reviewer for their generous comments. We likewise hope that this work will help spark a new wave of motion confusion/dazzle research.

Referee #4 (Remarks to the Author):

I was reviewer 4 in the first round of this manuscript. The authors have made substantial improvements, particularly by incorporating experimental validation of the model outputs. This directly addresses a major concern raised previously and significantly strengthens the paper. The authors have also made a commendable effort to incorporate and respond to all other reviewers' comments. That said, I still have one concern regarding the newly added results.

The capture experiments are an important component of the manuscript, but their interpretation raises an important concern. The results focus primarily on attack distribution, and although the Supplementary Information presents hits across body parts, overall capture success or failure rates are not reported. These would be critical for evaluating whether the observed confusion translates into a meaningful reduction in capture success, rather than simply increasing error, which is central to the biological relevance of the proposed mechanism. I suggest that the authors include these results, at least in the Supplement, for example by reporting the proportion of successful captures across treatments.

[Response] At the outset of our experiment, we designed our butterflies to occupy a consistent visual area across treatments, given that our core focus is on the influence of wing patterns. However, there are large differences in wing shape between the species selected, and the swallowtail's long tails result in a vastly larger bounding area and, as a result, hitbox, due to the 30px expansion of the click coordinate. Please see the new supplementary material, Figure 32. These resulted in the swallowtail butterfly being hit more. While the wing areas were equal, the area where a hit could be detected was greater for the swallowtail. Importantly, however, these tails and associated markings would be expected to deflect attacks towards these expendable tail sections, as seen in nature for swallowtails and for lizards.

Chotard, A., Ledamoisel, J., Decamps, T., Herrel, A., Chaine, A.S., Llaurens, V. and Debat, V., 2022. Evidence of attack deflection suggests adaptive evolution of wing tails in butterflies. Proceedings of the Royal Society B: Biological Sciences, 289(1975), p.20220562.

Arnold, E.N., 1984. Evolutionary aspects of tail shedding in lizards and their relatives. Journal of Natural History, 18(1), pp.127-169.

A successful hit to a butterfly does not equate to death. Numerous butterfly species are commonly recorded with damage to the tips of their forewing and, as above, to the trailing edges of the hind wings. Our evidence of attack redirection to the back of butterflies supports our hypothesis regarding the adaptive evolution of butterfly wing tails. When tail hits were counted as hits, no significant differences in hit probabilities were observed between butterflies, though having a larger hitbox area at the time of a hit did increase the probability of a hit. Crucially, if tail clicks were excluded as hits due to them being expendable, then the swallowtail was significantly less likely to be clicked on than all other butterflies. This is because 36% of hits for the swallowtail butterfly were at the tail. For full transparency, we have now included these analyses in the Supplementary Materials, but we maintain our focus on continuous metrics in our main-text analysis for the reasons detailed above.

1. Line 11-12: Would 'high-contrast butterfly-like wing patterns' be more accurate here, given that the opening sentence highlights the importance of high-contrast patterns in interfering with motion perception?

[Response] We have reworded as suggested. L11-12

2. Line 36: Should it be 'motion misperception' instead of 'motion detection'? Because the study shows that these patterns produce misleading motion cues rather than preventing motion detection altogether.

[Response] We have reworded as "motion perception".L37.

3. Line 38: I raised this point previously, but it remains unclear. The link between motion and any enhancement of deflection via eyespots during movement is not well explained. Are the authors suggesting that eyespots could also enhance confusion during motion?

[Response] Indeed, evidence for a direct interaction of eyespots and motion is currently lacking (the cited reference includes studies with free-flying butterflies, but the role of motion is not considered in detail in this paper and the butterflies tested do not have high-contrast wing markings other than eyespots). We have reworded to “high contrast markings” and now focus on evidence relating to hindwing tails in this sentence.L38-39

4. Line 59: ‘real wing patterning’ change to ‘natural wing patterning’ to keep it consistent.

[Response] Rephrased as requested.L57

5. Line 65: Should this be higher forward energy? The authors appear to refer to these results again in the next section (lines 101–102), so it would help to define this upfront.

[Response] Thanks, we have clarified that this refers to increased EMD values across all directions L65-66.

6. Line 96-100: It may be helpful to include an illustration in Figure 2 or in supplement highlighting these features (e.g., numbered and labelled alongside). For example, it is not immediately clear without referring to the Supplement what is meant by increased forewing contrast or how it differs from higher hindwing luminance.

[Response] Unfortunately, we could not include such an illustration due to limited space in the main figure: the sheer number of features measured >70, and the arbitrariness of creating illustrations for each feature meant that we didn’t want to mislead the reader. We have included plots in the Supplementary Material (Supplementary Fig.12-14(that visualise these dimensions and have now referred the reader to these for greater clarification in the main text L126-128.
.

7. Line 110-112: It is not entirely clear how these results differ from those presented above for features associated with sideways-confusion (lines 100–101). Are these results specific to the two families considered here? If so, why are features associated with forward confusion not presented as well? Please clarify.

[Response] In the first paragraph, we considered RF models only, looking at how butterfly features affect motion confusion outputs (forwards and sideways confusion L92-105. We have revised the text for the following paragraph to make it clearer that we are considering in more detail how these traits are distributed within the phylogeny in fig 2b L107-111.

8. Line 124 & 138: From the figure, it appears that the butterfly with the second-lowest forward confusion (Gonepteryx sp) shows a higher attack density entirely outside the body outline, which could be misinterpreted. It may be helpful to include a brief explanation of why elongated or elliptical attack patterns are expected when patterns produce forward-confusion in text or in the legend.

[Response] Thanks, this is a good point and we have now included this at L126-128.

9. Line 125: Please provide the specific supplementary table number here.

[Response] We have now added these. L126.

10. Line 128: Is this confusion quantified for flapping only, or for a mix of flapping and gliding? Please give some info in the legend.

[Response] This is confusion quantified for flapping only, and we have now clarified this at L132.

11. Line 132: Unclear which points the authors are referring to in the phylogeny.

[Response] Apologies, we changed from points in the phylogeny to bars during a previous round of revisions, and the text did not get updated properly here. This is now fixed. L136-137.

12. Line 154: Should ‘patterns that were effective’ be replaced with ‘patterns that produced’ here? This is because effectiveness is not directly quantified, and the results are instead presented in terms of metrics such as forward confusion.

[Response] We have modified this as suggested.L158.

13. Line 208: The study below may be more relevant and recent, as it tests both capture success and speed perception of multiple striped objects within the same experimental setup.
Kodandaramaiah, Ullasa, et al. "What makes motion dazzle markings effective against predation?." Behavioral Ecology 31.1 (2020): 43-53.

[Response] Thanks for this suggestion, we have now included this reference.

14. Line 251-252: It would be helpful to justify how well this value generalises across a range of relevant predators.

[Response] Acuity scales with the size of the bird (allometric scaling) - so should generalise to a range of bird species, with larger birds attacking larger butterflies. We have added a sentence and reference to this effect at L255

.

15. Line 285-287: This section ends abruptly. Seems like some text may be missing. There are also formatting inconsistencies throughout the Methods, and variable names are not used consistently, giving an overall impression of incomplete or insufficiently checked sections. Please review carefully.

[Response] Apologies, this was an issue with the tracked changes version of the manuscript and has now been fixed. L255.

16. Line 349 onward: Please clarify which type of flight was used in the main analysis (flapping vs gliding).

[Response] We have edited now to clarify the type of flight (flapping) used throughout the methods.

17. Line 384: Were the average grey and white butterflies only included in the RF models? The main figure appears to show confusion values only for natural patterns, so it is not entirely clear why these comparisons were included.

[Response] We did not include grey and white butterflies in the RF models. These were used only in the initial EMD analyses of the real butterflies, and in linear model comparisons in the supplementary material to test if patterns tend to increase motion confusion. We have included more signposting in the Methods section to make it clearer which parts of the results each section refers to.

18. Line 424: Please include details of the human-based capture experiments here, as this section is entirely missing.

[Response] Thanks for this point. We have now moved the human experiment descriptions into the main text.

19. Line 506: Again, the statistical analyses used to test attack distributions are not described. Please provide these details.

[Response] See response above - we do not think it would be meaningful to analyse attack distributions in this way.

20. Line 507: “LMs” or “LMMs”? Please define.

[Response] We have clarified that we mean LMMs here.

21. Line 513: By 'natural butterflies', do the authors refer to all 757 morphotypes rather than the seven used in the initial analysis? Please clarify this in the text throughout.

[Response] We have clarified this at L660.

Referee #5 (Remarks to the Author):

I have reviewed a previous version of this ms. and am satisfied that the main findings are sound, original and significant. The insight is not intuitively obvious. The authors might mention that the question of how velocity is estimated from motion energy, and the extent to which sideways confusion is due to the aperture effect, but this points do not critically alter the conclusions. What we have is a wonderful insight into the function of butterfly flight, wing shape and coloration.

[Response] The reviewer raises very interesting points. We avoid speculating on how velocity is estimated from motion energy, as this is not well understood, even in humans. Similarly, the extent to which sideways confusion is due to the aperture effect is interesting but may be less relevant with moving surfaces that are constantly changing shape and direction, meaning that there is no static aperture. While these questions warrant future exploration, they are beyond the scope of this paper.

Referee #5 (Remarks on code availability):

None

Comments on your responses to R1's earlier comments -- From R2

R2 on R1 comments

I've read R1's review and the authors' responses, and think that if I were the reviewer making these points, I would be satisfied. If they were to remain concerned, I think it would be around the use of a single 'clap and fling' wing dynamic in both the computational motion analysis and the behavioural experiments. I mention this in case it is the sticking point for other reviewers too. Personally, I think this is reasonable, given the limited data that exists regarding butterfly flight dynamics. As the authors note, this is for future work.

[Response] Thank you; this does not appear to be a major sticking point for the other reviewers, and we agree with your general conclusion that this is a reasonable simplification given the current state of knowledge about butterfly flight dynamics.

Comments on your responses to R1's earlier comments - From R4

Thank you for the opportunity to comment on this manuscript again. I have carefully reviewed the authors' responses to Reviewer 1.

One of the major concerns raised by Reviewer 1 in the first round of review was the lack of behavioural evidence supporting the authors' central claims. The authors have now addressed this concern through the addition of new experiments with human predators, which substantially strengthen the manuscript.

However, I note that the concerns I raised in my second-round review regarding the interpretation of these new results remain and should be considered separately.

[Response] Please see our main response to R4 above.

In addition, I found several of the authors' responses regarding assumptions related to viewing distance, flight dynamics, and background effects to be reasonable. However, many of these justifications appear only in the response letter, not in the manuscript itself. If possible, I would strongly suggest that the authors incorporate these assumptions, their biological rationale, and the limitations on generalising beyond the specific conditions modelled directly in the methods section. This would improve transparency and help readers evaluate and test these predictions in natural systems.

[Response] Thank you. We have now modified the methods section to include more of our justifications.

L252-263 “For our model, videos were first processed by spatially and temporally filtering contrasts not visible to the viewer to create ‘fluid motion vision’. Spatial contrast limits were based on those of small passerines (~ 6 cycles/degree) observing at 34cm (smallest) to 193cm (largest) away, depending on the size of the butterfly. Avian spatial acuity scales with eye and body size[46], so these viewing parameters should scale allometrically with larger or smaller predators and butterflies. Temporal limits were chosen based on a 100Hz critical flicker fusion frequency (cFFF) for a passerine within a forest habitat [46,47].We chose these distances to be as biologically realistic as possible: they are close enough to the target that an avian predator would be likely to initiate their ballistic attack. Closer distances are challenging to model, as they require very high frame rates to avoid motion artefacts (this viewing distance already required simulations at 2,000 fps). Meanwhile, longer viewing distances lead to the stripes no longer being resolvable, and are therefore irrelevant for the current hypotheses and attack ranges.”
”

The authors also appear to have addressed the remaining comments satisfactorily.

[Response] Thank you.

Comments on your responses to R1’s earlier comments - From R5

R1: The manuscript is likely to be of wide interest, ….. The key question in my mind while reading the manuscript is: does “motion confusion” as measured here actually confuse predators? …. In my opinion, this has the potential to be a classic and extremely well-cited paper if future behavioural experiments back up the model, but I’m not sure that is guaranteed.

AUTHOR: Thank you for your positive assessment of our work. We are pleased to say that we have now completed behavioural validation of our results using human ‘predators’ trying to catch moving butterflies on a touch screen…... Further details are available in the main manuscript (Figure 2, lines 56-68 and Supp material section 4), along with detailed descriptions of the experiment in the Supplementary Material (Section 4).

R5: This is a perfectly reasonable response. The ideal test would be to show that patterns caused birds to make errors in line with model predictions, but in my view it would be unreasonable to reject the work at this point for that reason. The evidence presented is impressive for the range of methods used to make the case for motion confusion and, as I said in my review, far more convincing and comprehensive than anything else in the literature. It is also novel & unexpected.

[Response] Thank you and yes we agree birds would be the ideal experiment subjects though there are numerous hurdles that need to be overcome to design a similar game for birds with speeds that are of comparable levels to butterflies.

R1 The authors have generally been careful in their language to avoid over-stretching the conclusions (e.g. line 163 "consistent with selection for motion confusion", line 166 "how motion confusion could operate"). …. but can we say that the motion illusion is "powerful" (line 1) when we don't know its effect? Can we say that motion confusion is an evolutionary "strategy" (line 12) when we don't know if it could be simply a biproduct of selection for warning colouration? For consideration for future work: are there differences between the patterns we would predict under motion confusion vs aposematism that could be used to separate the two strategies?

AUTHOR:Our behavioural validation supports the use of the word "powerful", as we now have evidence that patterning can indeed cause behavioural effects that would have a substantial effect on mortality (e.g. see the 38% of body length misdirection effect size). The evidence we present … is inconsistent with motion confusion being simply a by-product of warning coloration: for example, we find that butterflies rich in long-wavelength colouration (i.e. red), commonly associated with aposematism, are among the least motion confusing. We agree that better understanding the relationship between motion confusing and aposematic patterning is an important avenue for future research, but we would caution against setting these explanations up as mutually exclusive, as it is of course the case that one type of patterning could have multiple functions.

R5: I think this is a reasonable response. The authors could have done more to show that real lepidopteran aposematic patterns (e.g. on moths with high wing beat frequencies) that do not act to produce motion confusion are different from butterfly patterns, but this seems like a distraction. In addition it is thought that few European Butterflies are aposematic (either noxious or Batesian mimics), so the argument is irrelevant

[Response] Thanks for this relevant point, and we agree with your conclusion here.

R1 …., all modelling is based on images filtered to a certain level of visual acuity (line 229). Although this value has a biological basis, it is an angle, and so the amount of spatial information visible depends heavily on the viewing distance. On what distance were the results based, and how robust are the results to changes in that value?

AUTHOR: The results assume a distance of 34 to 193cm (depending on the size of the butterfly, from 1.08cm to 6.13cm respectively) to a small bird with spatial acuity of 6 cycles-per-degree. We chose these distances to be as biologically realistic as possible: they are close enough to the target that an avian predator would be likely to make their feedforward, ballistic attack. In addition, closer distances are challenging to model, as they require very high frame rates to avoid motion artefacts (this viewing distance already requires simulation at 2,000 fps). Longer viewing distances lead to the stripes no longer being resolvable, and are therefore irrelevant for the current hypotheses. We have scaled viewing distances to body-size (rather than fixed across all butterflies) because predator detection distances and responses will also scale with butterfly size.

R5: I think this is a reasonable response.

[Response] Thank you.

R1: Animation of butterflies was based on the flight pattern of a single species (line 327). The authors adjust for speed and wingbeat frequency but the "shape" (I can't think of a better word) of wing motion is constant across all animated species. I would imagine that there might be a strong interaction between wing morphology and flight patterns. How much do species vary in their flight patterns, and how much effect does the flight pattern have on the measures of confusion? Would it be valuable to carry out a test of robustness to changing the way in which the motion is animated?

AUTHOR: Our behavioural validation work incorporates multiple flight trajectories (see Supplementary Materials, Section 4) and the effects are robust across different trajectories. We also focus on the wing dynamics at the point of take-off, when butterflies are likely to be most vulnerable to attack. On take-off, butterflies will be most likely to rely on high-amplitude clap-and-fling dynamics (that we have modelled), which are likely to be more conserved among species than later steady flight dynamics that vary among taxa. In addition, high temporal and spatial resolution 3D data for butterfly free-flight wing dynamics and flight patterns do not currently exist. As

above, while there are clearly many interesting questions to ask about flight patterns, we would suggest these are beyond the scope of the current work.

R5: I think this is a reasonable response.

[Response] Thank you.

R1: There are some interesting effects of background matching that are evident in the supplementary information (lines 138-141 and Supp Fig 8). It seems that background matching, and hence the background against which patterns are viewed, can have a strong effect on forwards confusion, particularly for low-contrast patterns. In fact, the highest values of forwards confusion (by some margin) arise not from patterned wings but from the averaged treatment. Does this mean that background matching could form an alternative motion-confusion strategy? How does this compare with the results of the evolutionary simulation? E.g. it appears from supp fig 17 that some "evolved" patterns have rather low contrast – is this adaptive in the context of the simulation? It would be valuable to discuss these points in the main text.

AUTHOR: Natural backgrounds are always heterogeneous in terms of pattern, so an animal moving across these backgrounds would always be visible, making a background-matching strategy impossible. In Supplementary Figure 8, we would caution that while there are indeed a number of outliers, they are a very small percentage (9%) of the total. These outliers would be expected due to interactions between the butterfly and local (random) background features, which on occasion would be expected to combine and create backwards motion cues by chance. Indeed, we have preliminary data looking at confusion metrics on different backgrounds (see below), and it is clear that while the absolute numbers do vary, the relative ordering of the effects is fairly stable, suggesting that motion confusion effects are not strongly dependent on background type. Regarding the contrast of evolved prey, it is important to note that these patterns have been put through a Gaussian spatial filter (simulating viewing distance and controlling for temporal aliasing effects), which has the effect of artificially reducing contrast. The unblurred evolved patterns have higher contrast (but we only show the wings as they were processed through the EMD).

R5: I think this is a reasonable response.

[Response] Thank you.

R1: The authors do briefly mention the above factors in the discussion (lines 189-194), suggesting that "Further work should also incorporate more realistic simulations that take into account … a wider range of flight dynamics, view angles and distances, and background types." But they are then somewhat dismissive of the importance: "we argue that these will likely add nuance to our findings, rather than changing any overall conclusions". Given points 3-5 above, I disagree that this can be stated with confidence. Based on the presented evidence, background can alter whether a plain or contrasting pattern is favoured by evolution. Viewing distance will determine whether a pattern is perceived as contrasting or plain in the first place. I therefore think that further consideration is needed of how much it is possible to generalise from the presented results to other viewing conditions.

AUTHOR: We hope that our points above help to clarify our reasoning. In brief, while we have only pilot tests of orientation and background effects, they support our assertion that the motion confusion effects, particularly for highly confusing species such as swallowtails, are remarkably robust. Exploring any one of these dimensions in full would be a considerable undertaking, and beyond the scope of the current study (which we believe is already extremely wide-reaching).

R5: I think this is a reasonable response.

[Response] Thank you.

R1: Given that the body has a much simpler motion trajectory than the wings, I wonder whether contrast between body and wings might be an important factor contributing to motion confusion? (Not necessarily requesting a change to the manuscript here, just making a comment)

AUTHOR: Indeed - in our evolution simulations, it is interesting to note that the contrast of the body always decreased when selecting for motion confusion. Pilot evolutionary lines included the inverse of forwards-confusion which resulted in butterflies with white bodies. This aligns with the observation that almost all butterflies have low-contrast body coloration.

R5: I think this is a reasonable response. The point made in the response is that the body would give a veridical motion signal thereby detracting from the confusion effect produced by the wings, though the referee's comment is vague in line with their general level of approach.

> [Response] Thank you.

R1: I found the key measures of forward confusion and sideways confusion very, well, confusing to get my head around in terms of how the values actually behave. After careful reading, it seems that "forwards confusion" is negative by default (i.e. in the absence of any misleading cues) and gets closer to zero as it increases, which I found counter-intuitive. If we're measuring confusion, would it be possible to rescale so that the minimum (no confusion) is zero rather than -1? And/or it might be helpful to add a bit more description to the main text to help orient readers to what the numbers mean. Descriptions in the methods section are helpful in this regard (such as lines 253-4) but are less prominent especially on a first read-through.

AUTHOR: We have updated Figure 1 to include arrows and its caption to aid understanding of forward and sideways confusion. Because the metrics are based on Michelson contrast, we want to keep them on the same scale to aid readers who are familiar with this approach. We hope the changes to the text make it easier to interpret.

R5: I think this is a reasonable response. This is not a substantive point, but the original figure did take some thought to understand.

> [Response] We apologise for the initial vagueness of the figure and we are glad that the tweaks have amended this issue in the eyes of our reviewers.

R1: No results are presented to back up the statement about motion confusion being ancestral (line 194). Have the authors carried out ancestral reconstruction based on their data in figure 2b?

AUTHOR: We have not carried out ancestral state reconstruction, so have now removed this sentence as it was misleading.

R5: I think this is a reasonable response.

> [Response] Thank you.

R1: What was a typical evolutionary trajectory like over the course of the 20 generations in the simulation (line 422)? Were phenotypes converging on an asymptote by the end of the simulation? How was the duration of 20 generations chosen, and what would happen if simulations were left to run for longer?

AUTHOR: We have now included a graph in the supplementary material to help answer this question (See section 3.iii). This clearly shows that the phenotypes for most populations were converging on an asymptote by the end of the simulation. We chose the duration of 20 generations based on previous research that has shown this is generally sufficient (e.g. https://www.biorxiv.org/content/biorxiv/early/2025/03/06/2025.03.01.640995.full.pdf,https://academic.oup.com/evolut/article/76/5/870/6881792).

R5: I think this is a reasonable response.

> [Response] Thank you.

R1: All figures and statistical tests should quote sample sizes. Boxplots (e.g. Fig 1b) and error bars (e.g. Fig 3c) need defining.

AUTHOR: We have now provided the sample sizes and error bar descriptions for all plot figure legends.

R5: I think this is a reasonable response.

[Response] Thank you.

R1: The manuscript is concise, well written and well argued. Some minor points requiring clarification or correction:

o Line 23: slightly over-generalised. Animal locomotion doesn't require limb movements, or even limbs.

AUTHOR: We have added "often". L24

o Line 31-32: can you provide examples of where butterfly wing patterning cannot be explained by pre-existing theories?

AUTHOR: As noted by Reviewer 3 below, the evidence base for many of the pre-existing theories of butterfly wing patterning is actually surprisingly limited, particularly for European species. As we mention above, we think it is important to recognise that there is no reason to consider different theories to be mutually exclusive, and we expect that motion confusion is one of many selective pressures on butterfly patterning.

R5: I think this is a reasonable response.

[Response] Thank you.

Referee #4 (Remarks to the Author):

The authors have satisfactorily addressed my previous concerns, especially regarding the interpretation of the behavioural capture experiments. I am happy with the revisions and have only a couple of minor comments below.

1. For Supplementary Fig. 32, it would be helpful to clarify in the caption whether the bounding area was calculated for each frame of the animation and then summarized across frames.

Author. The figure caption has now been clarified to make it clear that it is showing the distribution of bounding areas across each frame: "**Supplementary Fig.32 | Butterfly hitbox bounding area,** boxplots with violons showing the differences in distribution of bounding hitbox area across frames between the butterfly phenotypes. Colour images of butterflies were used from the Collins Butterfly Guide with permission from Richard Lewington."

2. The caption for Supplementary Figure 33 is missing.

Author. We have now added the missing caption: "**Supplementary Fig.33 | Hitbox hits with and without tail hits,** shows the proportions of hits for each butterfly with and without tail hits included. Colour images of butterflies were used from the Collins Butterfly Guide with permission from Richard Lewington."

Reporting Summary

"Please see the 'Checklists extended comments for authors' document for a list of figures and figure legends that require additional information."

Author. We have now responded to these and have updated the legends accordingly, please see 'Checklists extended comments for authors - responses'

"Please provide the version numbers of the following software in the reporting summary. For example: Blender.

Please ensure all the data collection/data analysis software/packages mentioned in the manuscript are also listed in the reporting summary (with version numbers). For example: CHRONOS.

Please provide the GitHub web-links for the custom codes in the manuscript as well, under the separate code availability section."

Author. Version numbers for MATLAB have already been provided. We are unable to provide a version number for CHRONOS as CHRONOS is camera manufacturer (hardware) used for filming. The model number has also been provided.

"Please ensure that only the information related to databases/datasets generated and/or used in the study are mentioned in this section of the RS and the data availability section of the manuscript.

Information regarding code availability should be provided directly in the "software and code" section of the Reporting summary and code availability section of the manuscript."

Author. References to code availability have been removed from this section.

"Please outline any potential self-selection bias or other biases that may be present and how these are likely to impact results."

Author. We have added the following statement: *'While there was some potential for self-selection bias from the method of recruitment, it was required as per University of Exeter's recruitment policies and was not expected to influence our hypotheses which are based on low-level motion detection and visual processing.'*

"For butterfly game, please clearly describe how sample size was determined, detailing any statistical methods used to predetermine sample size OR if no sample size calculation was performed, describe how sample sizes were chosen and provide a rationale for why these sample sizes are sufficient."

Author. We have added the following statement: *'We used pilot data from 20 participants to build simple simulations for whether a sample size of 100 was sufficient to detect significant differences within our dataset and we found a 90% power for detecting an estimated marginal mean difference in number of hits of 0.45'*

"For (1), please state how often the experiments were replicated or performed independently and confirm if all attempts at replication were successful.

For (2), please provide a rationale as to why this was not replicated in this study.

For (3) and (4), please confirm if all attempts at replication were successful."

Author. This section has now been modified to:

(1) Multiple flights were taken for each species which act as replicates, but no replication of the experiment was carried out.

(2) No additional replication was needed as this study entirely relied on 3D simulations where any replication would produce exactly the same result.

(3) 10 replicates were performed for each wing shape and selection pressure. Differences between the replicates were accounted for in the statistical analyses. But the experiment itself was not replicated.

(4) 600 replicates were carried out for each treatment group, across the 100 participants. But no replication of the actual experiment was carried out.

"For (2) and (3), please describe how covariates were controlled or if this was not applicable to the study, explain why."

Author. This section has now been modified to:

(1) No randomization, butterflies were filmed opportunistically in order of capture.

(2) No randomization required, because this was simulation data and no humans or animal observers were involved.

(3) No randomization required because this was simulation data and no humans or animal observers were involved.

(4) Participants were assigned a pre-generated random order sequence of trials from a list of 100. Participants undertook these sequences in order of arrival for the experiment.

For (1), (2) and (3), please provide a rationale as to why blinding was not performed in the study.

Author. This section has now been modified to:

(1) No blinding was required for this study, all butterfly footage was analyzed automatically using elementary motion detectors.

(2) No blinding was required for this study as it was a simulation.

(3) No blinding was required for this study as it was a simulation.

(4) All data was collected automatically by the touch screen game.

Please ensure that the following modules which are not relevant to your study, are marked as 'N/A': Antibodies, Eukaryotic cell lines, Palaeontology and archaeology, Clinical data, Dual use research of concern, Plants.

We observed 'Animals and other organisms' module is applicable and the corresponding module has been filled. Please check the box as "involved in the study".

Author. We have now ticked and marked these boxes. Interestingly making plants N/A does not remove the plant tab.

Please state here that no laboratory animals were used in the study.

Author. We have updated the statement, but we still list the captive species here as no other method has been provided to list them and they are not wild.

Species:
1. Hypolimnas misippu, 3x male sex and 3x female sex, adult
2. Papilio dardanus ochracea, 3x male sex and 2x female sex, adult
Origin:
No laboratory animals were used in the study. Species were captive reared from larvae as a part of a private butterfly collection as opposed to from laboratory lines. The exact age of the butterflies could not be determined. Butterflies that were heavily worn or damaged were avoided.

Please specify the age of wild animals here.

Author. Butterflies are now listed as "adult", and the following statement has been added: *"The exact age of the butterflies could not be determined. Butterflies that were heavily worn or damaged were avoided."*

Please state here that no field collected samples were used in the study.

Author. We have updated the statement to simply say: "*No field collected samples were used in the study.*"
