# Supplementary material for: Butterfly wing patterns in flight create powerful illusory motion cues
Source: Nature. 2026 Sep 30;658(8137):958–62. doi: 10.1038/s41586-026-11062-w (PMC13686948; doi:10.1038/s41586-026-11062-w)
Supplement: Supplementary file 3 [file 41586_2026_11062_MOESM1_ESM.docx]

Supplementary Material

**Butterfly wing patterns create powerful illusory motion cues**

George RA Hancock^1^, Emmanuelle Briolat^1^, Anna Hughes^2^, Laura A Kelley^1^, Jolyon Troscianko^1^*

*Corresponding author: [J.Troscianko@exeter.ac.uk](mailto:J.Troscianko@exeter.ac.uk)

1. Centre for Ecology & Conservation, University of Exeter, Penryn, TR10 9FE, UK

2. Department of Psychology, University of Essex, Wivenhoe Park, Colchester, CO4 3SQ

**1. Real butterfly take-off recordings**	**1**

1. i. Motion energy across frames	1

1. ii. Ratios of energy across directions	3

1. iii. Influence of patterning on motion confusion metrics	3

**2. Motion confusion across European butterflies**	**5**

2. i. Butterfly phylogeny principal coordinates	5

2. ii. Influence of sex on motion confusion metrics	7

2. iii. Influence of patterning and gliding on motion confusion metrics	7

2. iv. Empirical image analysis of butterfly wing patterning	10

2. v. Random forests for motion confusion metrics	12

2. vi. SHAP feature plots	16

**3. Butterfly artificial evolution with genetic algorithms**	**19**

3. i. Butterfly pattern generation	19

3. ii. ImageGA settings	23

3. iii. Evolved butterfly phenotypes	24

3. iv. Influence of selection treatment on motion confusion metrics	28

3. v. PCA loadings	29

3. vi. Pairwise comparison table	31

**4. Behavioural validation of motion confusion measures for butterflies**	**32**

4. i. Summary	32

4. ii. Butterfly species	32

4. iii. Butterfly animations and flight behaviour	34

4. iv. Rendering and EMD measurement	37

4. v. Butterfly game	38

4. vi. Data collection and statistical analyses	40

4. vii. Results	40

**References**	**46**

# 1. Real butterfly take-off recordings

## 1. i. Motion energy across frames

**Supplementary Fig.1 | Motion energy and confusion across frames, *a.*** example motion forwards and backwards motion energy level for a red admiral, *Vanessa atalanta,* in free flight with is natural patterning and with its patterned averaged. Backwards energy is depicted as negative as it is an opponent to forwards energy. Lines represent the forwards energy subtracted by the backwards energy. The initial peak in forwards and backwards energy represents the point of take-off for the butterfly and is also when backwards energy is at its highest. Patterning increases the overall energy compared to the unpatterned and increases the ratio of backwards energy in proportion to forwards energy at multiple instances during flight. **b.** Shows the position of the butterfly across time, starting from the bottom, by masking the butterfly for each frame, adding each frame together, and then dividing by the number of frames. Note, as the butterfly does not fly in a straight line forwards and backwards, energy will not always correspond with the heading of the butterfly.

## 1. ii. Ratios of energy across directions

**Supplementary Fig.2 | Motion energy ratios,** depicts the average motion energy (EMD output) across all frames for all butterfly flights, with the different colour treatments. Energy is split into the four cardinal directions (backwards, forwards, left, and right). The left panel shows the raw average, while the right panel shows the energy divided by the greatest energy value across the four directions. Energy in general is higher for the white treatment compared to other treatments as the white is more contrasting against the background, followed by the patterned. Butterfly wing patterning overall increases the proportion of backwards, left, and right energy compared to forwards. Illustrations of treatments were created by George RA Hancock.

## [1. ii](http://1.ii)i. Influence of patterning on motion confusion metrics

Forwards-Confusion

**Supplementary Table 1** **| Tukey posthoc test for real butterfly take-off forwards-confusion**

| **Contrast** | **Estimate** | **SE** | **DF** | **T ratio** | **P value** |
| --- | --- | --- | --- | --- | --- |
| Patterned vs Averaged | 0.079 | 0.007 | 9350 | 11.504 | <0.001 |
| Patterned vs Black | 0.078 | 0.007 | 9350 | 11.478 | <0.001 |
| Patterned vs White | 0.078 | 0.007 | 9350 | 11.472 | <0.001 |
| Averaged vs Black | 0 | 0.007 | 9350 | -0.026 | 1 |
| Averaged vs White | 0 | 0.007 | 9350 | -0.032 | 1 |
| Black vs White | 0 | 0.007 | 9350 | -0.006 | 1 |

Sideways-Confusion

**Supplementary Table 2** **| Tukey posthoc test for real butterfly take-off sideways-confusion**

| **Contrast** | **Estimate** | **SE** | **DF** | **T ratio** | **P value** |
| --- | --- | --- | --- | --- | --- |
| Patterned vs Averaged | 0.044 | 0.005 | 9352 | 9.538 | <0.001 |
| Patterned vs Black | 0.044 | 0.005 | 9352 | 9.538 | <0.001 |
| Patterned vs White | 0.044 | 0.005 | 9352 | 9.538 | <0.001 |
| Averaged vs Black | 0 | 0.005 | 9352 | 0.105 | 1 |
| Averaged vs White | 0 | 0.005 | 9352 | 0.104 | 1 |
| Black vs White | 0 | 0.005 | 9352 | -0.001 | 1 |

Forwards-Energy

All butterfly pattern treatments were significantly different from one another for forwards-energy. On average butterflies with a higher contrast against the background (white) have increased forwards-energy. Patterning increases forwards-energy compared to averaged and produces marginally greater forwards energy then black, likely due to the white colours present on the different butterfly species. The averaged butterflies had significantly lower forwards energy then all other treatments due to having the lowest contrast from the background.

**Supplementary Table 3** **| Tukey posthoc test for real butterfly take-off forwards-energy**

| **Contrast** | **Estimate** | **SE** | **DF** | **T ratio** | **P value** |
| --- | --- | --- | --- | --- | --- |
| Patterned vs Averaged | 0.081 | 0.004 | 9362 | 22.286 | <0.001 |
| Patterned vs Black | 0.010 | 0.004 | 9362 | 2.629 | 0.0426 |
| Patterned vs White | -0.415 | 0.004 | 9362 | -114.567 | <0.001 |
| Averaged vs Black | -0.071 | 0.004 | 9362 | -19.658 | <0.001 |
| Averaged vs White | -0.496 | 0.004 | 9362 | -136.854 | <0.001 |
| Black vs White | -0.424 | 0.004 | 9362 | -117.196 | <0.001 |

**Supplementary Fig.3 | Influence of patterning on forwards-energy,** boxplots with violins for forward energy across all frames for all 7 butterfly wing morphotypes with patterning, and without patterning (averaged, black, and white). Illustrations of treatments were created by George RA Hancock.

# 2. Motion confusion across European butterflies

## 2. i. Butterfly phylogeny principal coordinates

For our principal coordinates:

High PCo1 values corresponded with Nymphalids, predominantly Satyrinae, while low values corresponded with Lycaenidae.

High PCo2 values corresponded with Hesperiidae and to a lesser extent Pieridae and Papilonidae, while low values corresponded with Lycanidae and Nymphalidae.

High PCo3 values corresponded with Hesperiidae while low values corresponded with Pieridae. Intermediate values comprised all other families.

See Supplementary Fig. 4 and Fig. 5.

**Supplementary Fig.4 | Principal coordinates for European butterflies,** plots show the principal coordinates (PCos) for the 6 European butterfly families. The left-hand plot shows the positions of PCo1 and PCo2, while the right-hand plot shows PCo1 and PCo3.

**Supplementary Fig.5 | Circular phylogeny of PCo values,** plots show the three principal coordinates (PCos) for the 6 European butterfly families in relation to the shorter phylogeny. Colour values for the inner circle denote the butterfly family and colour values for the outer circle denote the PCo value with lighter blue indicating positive values (+) and darker values denoting negative (-).

## 2. ii. Influence of sex on motion confusion metrics

**Supplementary Fig.6 | Influence of sex on butterfly motion confusion for flapping flight,** each plot shows a boxplot for each of the two sexes and points with lines linking members of the same species.

Given that many butterfly species were illustrated as sexually dimorphic in appearance (358 of the 397 species) and males are frequently more contrasting in appearance than females, we opted to compare our three motion confusion metrics between males and females (See Supplementary Fig.6). To compare each sex we used linear mixed models with the motion confusion metric as the response variable and the sex as the predictor variable with the species binomial as a random effect.

Males were found to have no significant difference in forwards-confusion (male vs female, forwards-confusion: β = 0.006, t value_357_ = 1.82, p = 0.0696), marginally lower forwards-energy than females (male vs female, forwards-energy: β = -0.057, t value_357_ = -2.073, p = 0.039), and significantly greater sideways confusion (male vs female, sideways-confusion: β = 0.025, t value_357_ = 6.49, p < 0.001).

## 2. iii. Influence of patterning and gliding on motion confusion metrics

To confirm our results for the influence of patterning on motion confusion in butterflies for the real butterfly take-offs, we repeated the comparison of motion confusion measures for butterflies with their natural wing patterns and those without their wing patterns (white and averaged wing luminance). Numerous butterfly species, in particular larger species, undergo periods of unpowered flight where the wings are held open in a glide. These periods of differential patterns of biological motion are likely to influence the intensity of motion in different directions and as a result motion confusion. As the 3D blender model allowed us to render butterfly flights with and without flapping flight we also quantified how wing patterning influenced the EMD when gliding (no flapping).

To compare the effects of patterning and gliding for each motion confusion metric we used linear mixed models. The confusion metric was given as the response variable, and both the pattern treatment (patterned, averaged, or white), the flight method (gliding or flapping) and the interaction between them were used as predictor variables. The unique morphotype of the butterfly was used as a random effect.

As with the real butterflies patterning increased motion confusion compared to the unpatterned treatments, however motion confusion was in some instances higher for the averaged butterflies when the butterflies near perfectly matched the background average (See Supplementary Fig.7 and Table 4-6). Flapping flight was found to be integral to the generation of forwards-confusion effects with the level of backward motion detected being near zero in the absence of wing movement. Meanwhile gliding exacerbated the difference between patterned and the unpatterned treatments for sideways-confusion and increased the level of forward-energy across all treatments (given the reduction in movement in alternative directions), but did not interact with patterning for forward-energy. Outliers for flapping flight where averaged butterflies caused high degrees of forwards and sideways motion confusion were due to instances where the butterflies matched the luminance of the patternless background (See Supplementary Fig. 8).

**Supplementary Fig.7 | Influence of pattern and gliding on motion confusion metrics,** levels of forwards-confusion, sideways-confusion, and forwards-energy for simulated butterfly flight when flapping their wings (above) and when their wings are static (gliding). Butterflies were rendered either with patterns, their averaged luminance or as white. Illustrations of treatments were created by George RA Hancock.

**Supplementary Fig.8 | Influence of background match on motion confusion metrics,** how does log difference in luminance from the background influence motion confusion for the three pattern treatments, averaged, patterned, and white. Illustrations of treatments were created by George RA Hancock.

**Supplementary Table 4** **| Linear mixed model for forwards-confusion, gliding and pattern type**

| **Contrast** | **Estimate** | **SE** | **DF** | **T ratio** | **P value** |
| --- | --- | --- | --- | --- | --- |
| Patterned vs Averaged | -0.012 | 0.002 | 3780 | -5.217 | <0.001 |
| Patterned vs White | -0.031 | 0.002 | 3780 | -13.739 | <0.001 |
| Gliding vs Flapping | -0.378 | 0.002 | 3780 | -167.952 | <0.001 |
| Patterned vs Average : Gliding | 0.012 | 0.002 | 3780 | 3.635 | <0.001 |
| Patterned vs White : Gliding | 0.031 | 0.002 | 3780 | 9.659 | <0.001 |

**Supplementary Table 5** **| Linear mixed model for sideways-confusion, gliding and pattern type**

| **Contrast** | **Estimate** | **SE** | **DF** | **T ratio** | **P value** |
| --- | --- | --- | --- | --- | --- |
| Patterned vs Averaged | -0.044 | 0.004 | 3780 | -11.05 | <0.001 |
| Patterned vs White | -0.082 | 0.004 | 3780 | -20.54 | <0.001 |
| Gliding vs Flapping | -0.143 | 0.004 | 3780 | -35.51 | <0.001 |
| Patterned vs Average : Gliding | -0.177 | 0.004 | 3780 | -31.21 | <0.001 |
| Patterned vs White : Gliding | -0.140 | 0.004 | 3780 | -24.55 | <0.001 |

**Supplementary Table 6** **| Linear mixed model for forwards-energy, gliding and pattern type**

| **Contrast** | **Estimate** | **SE** | **DF** | **T ratio** | **P value** |
| --- | --- | --- | --- | --- | --- |
| Patterned vs Averaged | -0.991 | 0.046 | 3780 | -21.658 | <0.001 |
| Patterned vs White | 2.536 | 0.046 | 3780 | 55.412 | <0.001 |
| Gliding vs Flapping | 0.862 | 0.046 | 3780 | 18.837 | <0.001 |
| Patterned vs Average : Gliding | -0.102 | 0.046 | 3780 | -1.578 | 0.115 |
| Patterned vs White : Gliding | 0.018 | 0.046 | 3780 | 0.282 | 0.778 |

## 2. iv. Empirical image analysis of butterfly wing patterning

To quantify the appearance of butterfly wings we used image statistics maps for the forewing and hindwing to allow us to measure the mean, standard deviation, x-axis gradient, and y-axis gradient of each statistic. For the gradients a positive gradient indicates an increase in the map’s value from left to right / top to bottom of the image, and a negative gradient indicates a decrease. To illustrate how these maps work we provide examples for three butterfly wings (See Supplementary Fig 8). For each map the largest spatial scale, s6, was equivalent to ¼ the square root of the wings area in pixels.

For each butterfly, metrics are split between the forewing and hindwing, e.g. fw.pat.E.mean is the mean energy for the forewing and hw.pat.VH.stdev is the variation in vertical horizontal orientation for the hindwing. Pat stands for patterning measure and is used to distinguish wing shape measures, .shp.

L = luminance, P =periodicity, VH = vertical-horizontal, OA = obtuse - acute, and DR = directionality.

**Luminance map -**

Simply created by converting the image from sRGB to blue-tit double cone quantum catch.

**Periodicity map -**

For each pixel, periodicity is calculated as the weighted average spatial scale (S={s1,s2,…,s6}), where each scale is weighted by the absolute value of the difference of gaussian (DoG) output. Higher periodicity (values closer to 1) indicate larger spatial scales.

Px = pixel value

s = scale

wS = $\sum_{s\in S}^{s} \frac{abs(px)s}{s}$ = Weighted Sum

E = $\sum_{s\in S}^{s} abs(px)s$= Total Energy

Periodicity = $\frac{wS}{E}$

**Energy map -**

For each pixel, energy is calculated as the sum absolute value of each pixel across spatial scales (S={s1,s2,…,s6}).

px = pixel value

s = scale

E = $\sum_{s\in S}^{s} abs(px)s$ = Total Energy

**VH, OA & Directionality maps-**

Unlike periodicity and energy, these metrics use Gabor filters at six orientations (0, 30, 60, 90, 120, and 150 degrees) but at the same six spatial scales as the DoG. These orientations are then converted into spatial maps in four different directions by calculating the energy across each orientation.

px = pixel value

a = angle in radians

V (0 degrees) = $\sum_{s\in S} sin(a)*abs(px)s$

H (90 degrees) = $\sum_{s\in S} cos(a)*abs(px)s$

A (135 degrees) = $\sum_{s\in S} sin(a*\frac{\pi}{4})*abs(px)s$

O (135 degrees) = $\sum_{s\in S} cos(a*\frac{\pi}{4})**abs(px)s$

Then each image (V, H, A, O) is used to create the following image statistics map. VH where each pixel is the value for V - H, OA where each pixel is the value of O - A, and Directionality where each pixel is the value is √(VH^2^ + OA^2^) - E, with positive values being more directional.

**Supplementary Fig.9 | Image statistics maps,** example image statistic maps for three different butterfly species. DBL, blue-tit cone catch for the wing downscaled so that the ¼ the wings area was 64 pixels. Lighter values indicate lighter regions. Periodicity, a spatial scale map where brighter values indicate higher spatial frequencies. Energy, brighter values indicate regions of greater internal contrast from the rest of the wing. Vertical - Horizontal, shows VH where red values are more vertical and green values are more horizontal. Acute - Obtuse, shows OA where yellow values are closer to 135 degrees and blue values are closer to 45 degrees. Directionality, where lighter yellower values indicate more directional patterns. Images of butterflies were used from the Collins Butterfly Guide with permission from Richard Lewington.

## 2. v. Random forests for motion confusion metrics

See the empirical image analysis of butterflies above for pattern variable denotation. Butterfly phylogeny is given using principal coordinates (PCos) 1-3.

**Supplementary Table 7** **| SHAP table for forwards-confusion**. Variables are ordered by absolute mean SHAP value.

| **Feature** | **Node purity** | **Mean SHAP ± std.dev** |
| --- | --- | --- |
| fw.pat.L.stdev | 0.6014 | 0.0051 ± 0.0109 |
| fw.pat.E.mean | 0.6713 | 0.0046 ± 0.0101 |
| hw.pat.VH.mean | 0.3468 | 0.0042 ± 0.0112 |
| hw.pat.L.mean | 0.5995 | 0.0038 ± 0.0157 |
| PCo3 | 0.1752 | -0.0021 ± 0.0054 |
| fw.pat.VH.mean | 0.4878 | 0.0019 ± 0.0135 |
| hw.shp.rough | 0.1854 | 0.0018 ± 0.0047 |
| fw.pat.L.mean | 0.2887 | 0.0014 ± 0.0058 |
| hw.pat.L.x | 0.1802 | 0.0012 ± 0.0046 |
| fw.pat.E.x | 0.2406 | 0.0012 ± 0.0075 |
| PCo1 | 0.1950 | 0.001 ± 0.0031 |
| hw.pat.P.mean | 0.1343 | -0.0005 ± 0.0038 |
| fw.pat.L.x | 0.2393 | -0.0003 ± 0.004 |
| fw.col.by.mean | 0.2451 | 0.0002 ± 0.0069 |
| PCo2 | 0.1347 | -0.0001 ± 0.0026 |

**Supplementary Table 8** **| SHAP table for sideways-confusion**. Variables are ordered by absolute mean SHAP value.

| **Feature** | **Node purity** | **Mean SHAP ± std.dev** |
| --- | --- | --- |
| hw.pat.VH.mean | 0.8423 | 0.0111 ± 0.0272 |
| hw.pat.L.mean | 1.1025 | 0.0054 ± 0.0234 |
| body.length | 0.3049 | 0.0024 ± 0.0104 |
| fw.pat.L.mean | 0.7927 | 0.002 ± 0.0161 |
| hw.pat.P.stdev | 0.3445 | 0.0018 ± 0.0066 |
| hw.shp.rough | 0.3436 | 0.0013 ± 0.0108 |
| PCo3 | 0.1722 | -0.0011 ± 0.0046 |
| hw.pat.DR.mean | 0.1333 | 0.0011 ± 0.0032 |
| fw.pat.VH.mean | 0.2764 | 0.0011 ± 0.0067 |
| hw.pat.L.x | 0.2066 | 0.0008 ± 0.0074 |
| hw.pat.L.stdev | 0.3355 | 0.0007 ± 0.0071 |
| fw.shp.length | 0.1267 | -0.0001 ± 0.0022 |
| hw.shp.length | 0.1788 | -0.0001 ± 0.0023 |
| PCo2 | 0.2653 | -0.0001 ± 0.0055 |
| hw.pat.P.x | 0.2025 | 0 ± 0.0048 |

**Supplementary Table 9** **| SHAP table for forwards-energy**. Variables are ordered by absolute mean SHAP value.

| **Feature** | **Node purity** | **Mean SHAP ± std.dev** |
| --- | --- | --- |
| hw.pat.L.mean | 3.7952 | 0.0081 ± 0.0641 |
| fw.pat.L.mean | 2.5395 | 0.0052 ± 0.0396 |
| fw.pat.E.mean | 0.4418 | 0.0026 ± 0.0099 |
| fw.pat.L.stdev | 0.2084 | 0.0022 ± 0.0079 |
| hw.pat.L.stdev | 0.0936 | 0.0019 ± 0.0051 |
| hw.pat.E.mean | 0.0858 | 0.0011 ± 0.0047 |
| PCo3 | 0.5321 | 0.001 ± 0.0057 |
| fw.pat.L.x | 0.6294 | 0.0007 ± 0.0071 |
| hw.pat.P.x | 0.0745 | 0 ± 0.0015 |

**Supplementary Fig.10 | SHAP values for motion confusion metrics,** the ranked SHAP values for the reduced forest tree for forwards-confusion, sideways-confusion, and forwards-energy. Positive values indicate instances where the feature increases the motion confusion metric and negative values indicate instances where the variable decreases it.

**Supplementary Fig.11 | Random forest accuracy plots,** the predicted motion confusion values against the observed motion confusion values for forwards-confusion, sideways-confusion and forwards-energy when using the reduced model.

## 2. vi. SHAP feature plots

Here, we provide scatter plots with the images for each butterfly for the top 8 SHAP variables not including the PCos.

**Supplementary Fig.12 | SHAP features for forwards-confusion**, shows the top 8 SHAPs for forwards-confusion, not including PCos. Each point shows the wing images for the specific butterfly at that point. Positive values indicate higher forwards-confusion for all SHAPs. Broadly, having more contrasting, vertically oriented patterns, brighter wings, and rough (tailed) hindwings predict increased forwards-confusion. Images of butterflies were used from the Collins Butterfly Guide with permission from Richard Lewington.

**Supplementary Fig.13 | SHAP features for sideways-confusion**, shows the top 8 SHAPs for sideways-confusion, not including PCos. Each point shows the wing images for the specific butterfly at that point. Positive values indicate higher sideways-confusion for all SHAPs. Broadly, vertically orientated patterns, that are brighter for butterflies with larger bodies as well as having rough hindwings (tails) and more directional patterns predict higher sideways-confusion. Images of butterflies were used from the Collins Butterfly Guide with permission from Richard Lewington.

**Supplementary Fig.14 | SHAP features for forwards-energy,** shows the top 8 SHAPs for forwards-confusion not including PCos. Each point shows the wing images for the specific butterfly at that point. Images of butterflies were used from the Collins Butterfly Guide with permission from Richard Lewington.

# 3. Butterfly artificial evolution with genetic algorithms

## 3. i. Butterfly pattern generation

Butterfly wing pattern generation was derived from the pattern generator included in the CamoEvo toolbox v2.0 <https://github.com/GeorgeHancock471/CamoEvo-v2.0-2022_Plugins>. This system uses reaction-diffusion patterns to generate biologically relevant patterns that can be evolved under different selection pressures.

The pattern generator was modified to allow for separate generation of pattern dimensions for the forewing, hindwing and body using masks (see supplementary Fig.15), to add a sin convolution function with different strengths to create eye spot like patterns, two separate colours for the maculation and the background (note butterflies were achromatic for this experiment), and to produce asymmetric edge enhancement patterns (see supplementary Table 10 for full trait list).

**Supplementary Table 10** **| Gene function table for butterfly pattern generator**

| **Gene Label** | **Function** |
| --- | --- |
| dim_for_xcp | Forewing reaction diffusion pattern x coordinate |
| dim_for_ycp | Forewing reaction diffusion pattern y coordinate |
| dim_for_wdt | Forewing reaction diffusion pattern selection width |
| dim_for_asr | Forewing reaction diffusion pattern selection aspect ratio |
| dim_for_agl | Forewing reaction diffusion pattern rotation |
| dim_hnd_xcp | Hindwing reaction diffusion pattern x coordinate |
| dim_hnd_ycp | Hindwing reaction diffusion pattern y coordinate |
| dim_hnd_wdt | Hindwing reaction diffusion pattern selection width |
| dim_hnd_asr | Hindwing reaction diffusion pattern selection aspect ratio |
| dim_hnd_agl | Hindwing reaction diffusion pattern rotation |
| dim_bod_xcp | Body reaction diffusion pattern x coordinate |
| dim_bod_ycp | Body reaction diffusion pattern y coordinate |
| dim_bod_wdt | Body reaction diffusion pattern selection width |
| dim_bod_asr | Body reaction diffusion pattern selection aspect ratio |
| dim_bod_agl | Body reaction diffusion pattern rotation |
| ptn_grd_cvr | Pattern area cover |
| ptn_grd_sig | Pattern gradient blur |
| ptn_grd_hgt | Pattern gradient radius |
| ptn_grd_xps | Pattern gradient x coordinate |
| ptn_grd_yps | Pattern gradient y coordinate |
| ptn_grd_sin | Pattern sin convolution, used to produce eye spot like patterns |
| ptn_bil_sub | Asymmetry noise subtraction before min = 0, for symmetrical patterns just adds noise to pattern shape. |
| ptn_bil_sig | Asymmetry noise blurring gaussian sigma, for symmetrical patterns just adds noise to pattern shape. |
| ptn_bil_int | Asymmetry noise intensity level, for symmetrical patterns just adds noise to pattern shape. |
| ptn_edg_sig | Wing edge, gaussian blur sigma of mask used to shape pattern to wing edge |
| ptn_edg_rto | Wing edge, ratio between positive and negative |
| ptn_edg_dfm | Wing edge, reform intensity |
| eem_int_lvl | Edge enhancement internal intensity level |
| eem_int_sig | Edge enhancement internal gaussian blur |
| eem_int_exp | Edge enhancement internal expansion level |
| eem_int_xst | Edge enhancement internal x offset |
| eem_int_yst | Edge enhancement internal y offset |
| eem_ext_lvl | Edge enhancement external intensity level |
| eem_ext_sig | Edge enhancement external gaussian blur |
| eem_ext_exp | Edge enhancement external expansion level |
| eem_ext_xst | Edge enhancement external x offset |
| eem_ext_yst | Edge enhancement external y offset |
| col_mc1_lmv | Maculation colour L* 1 |
| col_mc1_rgv | Maculation colour a* 1 |
| col_mc1_byv | Maculation colour b* 1 |
| col_mc2_lmv | Maculation colour L* 2 |
| col_mc2_rgv | Maculation colour a* 2 |
| col_mc2_byv | Maculation colour b* 2 |
| col_bg1_lmv | Background colour L* 1 |
| col_bg1_rgv | Background colour a* 1 |
| col_bg1_byv | Background colour b* 1 |
| col_bg2_lmv | Background colour L* 2 |
| col_bg2_rgv | Background colour a* 2 |
| col_bg2_byv | Background colour b* 2 |
| grd_mac_hgt | Maculation colour gradient radius |
| grd_mac_sig | Maculation colour gradient sigma |
| grd_mac_sin | Maculation colour gradient sin |
| grd_mac_agl | Maculation colour gradient angle |
| grd_bgd_hgt | Background colour gradient radius |
| grd_bgd_sig | Background colour gradient sigma |
| grd_bgd_sin | Background colour gradient sin |
| grd_bgd_agl | Background colour gradient angle |
| grd_blr_mac | Background colour gradient radius |
| spk_nm1_lgt | Speckling 1 light intensity |
| spk_nm1_drk | Speckling 1 dark intensity |
| spk_nm1_sig | Speckling 1 gaussian blur |
| spk_nm1_ycd | Speckling 1 y coordinate |
| spk_nm2_lgt | Speckling 2 light intensity |
| spk_nm2_drk | Speckling 2 dark intensity |
| spk_nm2_sig | Speckling 2 gaussian blur |
| spk_nm2_ycd | Speckling 2 y coordinate |

**Supplementary Fig.15 | Butterfly wing shape extraction,** shows how the wing shape masks and pattern area masks were made for a butterfly using a nymphalid as an example. First the wing was masked and then an area was drawn for the forewing, hindwing and body pattern. Red overlay shows the butterfly wing mask over the mask areas. White indicates a region where patterns are contained within. Images of butterflies were used from the Collins Butterfly Guide with permission from Richard Lewington.

**Supplementary Fig.16 | Example wing patterns,** shows one whole population (N=24) for each of the three butterfly wing shapes used. All patterns are from the first generation (generation=0) where each of the genes is randomly generated in a uniform distribution from 0-1 for each gene.

## 3. ii. ImageGA settings

Sex = **0 [False]**

Population Size = **24**

Number of Generations = **20**

Starting Gene Distribution = **Uniform**

Deletion Pool = **66.6%**

Breeding Pool = **33.3%**

Point Mutation Prob = **0.001**

Lvl1 Mutation Prob = **0.005**

Lvl2 Mutation Prob = **0.005**

Link Mutation Prob = **0.3333**

Rescue Number = **0 [False]**

Adaptive Mutations = **0 [False]**

Gene Displace Prob = 0.01

Adaptive Mutations = true

Crossover Type = **random**

Crossover Probability = **1.00**

Recombination = **incomplete**

Mating System = **random**

Breeding Pool Assignment = **ranked**

For CamoEvo’s genetic algorithm, the following settings were used.

Sex was disabled, meaning that any individual could mate with any individual in the breeding pool. The population size was set to 24 individuals with a uniform distribution for the starting genes as opposed to a random or normal distribution. Two-thirds of the population were deleted each generation, with the best third surviving and reproducing. The probability of a point mutation occurring for each gene was 0.001, for a lvl 1 group mutation 0.005, and a lvl 2 group mutation 0.005 ([see ImageGA handbook for more details](https://github.com/GeorgeHancock471/CamoEvo-v2.0-2022_Windows/blob/main/ImageGA_Guide.pdf)). The probability of a group mutation affecting other members of the group was 0.333. The rescue number was set to zero, as there was no noise in the simulation's selection of the fitness metrics. Genes with similar values had a 0.01% chance of being displaced during crossover, increasing diversity. Crossover for each gene was determined randomly, as opposed using one-point (single break) or two-point (two breaks) crossover. Recombination was incomplete, meaning that genes were combined with a weighted average simulating recombination for polygenic inheritance. Individuals within the breeding population were randomly paired and assigned by rank.

## 3. iii. Evolved butterfly phenotypes

**Supplementary Fig.17 | Forwards-Confusion evolved butterflies,** showing one individual for each population evolved for forwards-confusion from the final generation (generation=20) and separated by the three wing shapes. Colour images of butterflies were used from the Collins Butterfly Guide with permission from Richard Lewington.

**Supplementary Fig.18 | Sideways-Confusion evolved butterflies,** showing one individual for each population evolved for sideways-confusion from the final generation (generation=20) and separated by the three wing shapes. Colour images of butterflies were used from the Collins Butterfly Guide with permission from Richard Lewington.

**Supplementary Fig.19 | Forwards-Energy evolved butterflies,** shows one individual for each population evolved for forwards-energy from the final generation (generation=20) and separated by the three wing shapes. Colour images of butterflies were used from the Collins Butterfly Guide with permission from Richard Lewington.

**Supplementary Fig.20 | Random-Selection evolved butterflies,** showing one individual for each population evolved with randomised fitness values from the final generation (generation=20) and separated by the three wing shapes. Colour images of butterflies were used from the Collins Butterfly Guide with permission from Richard Lewington.

## 3. iv. Influence of selection treatment on motion confusion metrics

**Supplementary Fig.21 | Forwards- and sideways-confusion correlation,** shows the linear regression plot for forwards- and sideways-confusion for the unevolved generation 0 butterflies and the final generation 20 for the three different evolution treatments. Butterflies under random-selection were excluded as they weren’t measured for EMD.

**Supplementary Fig.22 | Change in fitness between start and end,** shows the scaled (mean = 0, std.dev = 1) fitness for the three evolution treatments for the three evolution treatments and separated by wingshape. Left to right and with decreasing luminance are the three wing shapes (Papilonidae, Nymphalidae, and Hesperiidae)

Butterflies significantly improved in fitness across generations for all non-random treatments when tested using a linear model with scaled fitness as the response variable and generation (start and end) as the predictor variable (end vs start, β = 1.788675, t value = 185.2, SE = 0.010, p <0.001). Butterflies under random-selection were excluded as they weren’t measured for EMD.

Fitness for most GA butterfly populations had started to plateau towards the final generation, particularly for those selected for Forwards-Energy, which typically peaked around generation 8 (See Supp Fig 23).

**Supplementary Fig.23 | Change in fitness across generations,** shows the cubic relationship between generation and fitness for each of the four GA selection pressures. Note how random remains flat. Faint lines show each individual population while the thick dark line shows the mean trend across all populations.

## 3. v. PCA loadings

**Supplementary Table 11** **| Ordered image pattern feature loadings for PC1 and PC2 variables are sorted by their absolute contribution. PC1 is primarily pattern contrast on the forewing and hindwing and PC2 is primarily pattern orientation and luminance.**

| **PC1** | | **PC2** | |
| --- | --- | --- | --- |
| ***Feature*** | ***Loading*** | ***Feature*** | ***Loading*** |
| fw.pat.DR.stdev | 0.268563 | hw.pat.OA.x | -0.29989 |
| hw.pat.E.mean | 0.267379 | hw.pat.VH.x | 0.282803 |
| fw.pat.E.mean | 0.264071 | fw.pat.VH.x | 0.278714 |
| fw.pat.VH.stdev | 0.263816 | hw.pat.OA.stdev | 0.258527 |
| fw.pat.OA.stdev | 0.263306 | fw.pat.L.mean | -0.25602 |
| hw.pat.L.stdev | 0.262894 | fw.pat.DR.x | 0.23709 |
| fw.pat.L.stdev | 0.262455 | fw.pat.DR.mean | 0.23574 |
| hw.pat.VH.stdev | 0.258653 | hw.pat.DR.stdev | 0.209364 |
| hw.pat.DR.stdev | 0.258521 | fw.pat.P.stdev | 0.207509 |
| hw.pat.E.stdev | 0.255711 | hw.pat.L.mean | -0.20327 |
| fw.pat.E.stdev | 0.254993 | hw.pat.E.x | 0.201842 |
| hw.pat.OA.stdev | 0.245247 | hw.pat.VH.stdev | 0.200999 |
| fw.pat.L.mean | 0.181333 | fw.pat.L.x | -0.1738 |
| hw.pat.L.mean | 0.180075 | fw.pat.E.mean | -0.16771 |
| fw.pat.L.x | 0.15633 | fw.pat.L.stdev | -0.1601 |
| fw.pat.P.stdev | -0.13928 | fw.pat.OA.mean | -0.1536 |
| hw.pat.P.stdev | -0.13533 | hw.pat.E.stdev | 0.151398 |
| hw.pat.P.mean | -0.12265 | fw.pat.VH.mean | 0.140332 |
| fw.pat.P.mean | -0.12035 | hw.pat.E.mean | 0.139249 |
| hw.pat.DR.mean | -0.05912 | hw.pat.DR.mean | 0.12115 |
| fw.pat.E.x | 0.055724 | hw.pat.DR.x | 0.118256 |
| fw.pat.DR.x | 0.055361 | hw.pat.L.x | 0.117725 |
| fw.pat.VH.x | 0.0539 | hw.pat.P.mean | -0.11541 |
| hw.pat.P.x | 0.049861 | fw.pat.P.mean | 0.114024 |
| hw.pat.OA.mean | -0.04717 | fw.pat.E.x | 0.112363 |
| hw.pat.OA.x | -0.04444 | fw.pat.DR.stdev | -0.09109 |
| fw.pat.DR.mean | -0.04351 | fw.pat.OA.stdev | -0.09089 |
| hw.pat.DR.x | 0.041132 | fw.pat.OA.x | -0.09034 |
| hw.pat.VH.x | 0.037225 | fw.pat.E.stdev | -0.07976 |
| fw.pat.VH.mean | 0.033612 | hw.pat.L.stdev | 0.070147 |
| hw.pat.L.x | -0.02826 | hw.pat.VH.mean | -0.06782 |
| fw.pat.P.x | 0.025351 | hw.pat.P.stdev | 0.062152 |
| fw.pat.OA.x | 0.02347 | fw.pat.VH.stdev | -0.03231 |
| hw.pat.E.x | 0.021196 | hw.pat.DR.y | -0.02788 |
| fw.pat.OA.mean | 0.01417 | fw.pat.OA.y | 0.018925 |
| hw.pat.VH.y | 0.01213 | hw.pat.P.x | -0.01756 |
| hw.pat.E.y | 0.009363 | hw.pat.E.y | -0.01744 |
| hw.pat.L.y | -0.00881 | hw.pat.P.y | 0.012326 |
| fw.pat.VH.y | -0.00772 | fw.pat.E.y | 0.010222 |
| fw.pat.DR.y | -0.00724 | hw.pat.OA.mean | -0.00961 |
| fw.pat.L.y | 0.002425 | hw.pat.VH.y | -0.00776 |
| fw.pat.OA.y | -0.00219 | fw.pat.P.x | 0.007445 |
| hw.pat.OA.y | -0.0021 | hw.pat.OA.y | -0.00628 |
| fw.pat.E.y | -0.00138 | fw.pat.P.y | -0.00521 |
| hw.pat.VH.mean | -0.00134 | fw.pat.VH.y | -0.00456 |
| fw.pat.P.y | 0.001317 | fw.pat.DR.y | -0.0044 |
| hw.pat.DR.y | -0.00032 | fw.pat.L.y | 0.004068 |
| hw.pat.P.y | -0.00031 | hw.pat.L.y | -0.00308 |

## 3. vi. Pairwise comparison table

**Supplementary Table 12** **| Tukey posthoc test for real pairwise comparisons between butterflies (GA generated natural) and all naturally occurring butterflies**

| **Contrast** | **Estimate** | **SE** | **DF** | **T ratio** | **P value** |
| --- | --- | --- | --- | --- | --- |
| Nature vs  Unevolved | -0.10241 | 0.000996 | 2847828 | -102.792 | <.0001 |
| Nature vs  Forwards-Confusion | 0.05566 | 0.00461 | 2847828 | 12.066 | <.0001 |
| Nature vs  Sideways-Confusion | -0.00096 | 0.00416 | 2847828 | -0.231 | 0.9999 |
| Nature vs  Forwards-Energy | -0.12157 | 0.00447 | 2847828 | -27.212 | <.0001 |
| Nature vs  Random-Selection | -0.06167 | 0.00454 | 2847828 | -13.589 | <.0001 |
| Unevolved vs  Forwards-Confusion | 0.15806 | 0.00455 | 2847828 | 34.742 | <.0001 |
| Unevolved vs  Sideways-Confusion | 0.10145 | 0.00409 | 2847828 | 24.813 | <.0001 |
| Unevolved vs  Forwards-Energy | -0.01916 | 0.0044 | 2847828 | -4.353 | 0.0002 |
| Unevolved vs  Random-Selection | 0.04074 | 0.00447 | 2847828 | 9.105 | <.0001 |
| Forwards-Confusion vs Sideways-Confusion | -0.05662 | 0.00608 | 2847828 | -9.308 | <.0001 |
| Forwards-Confusion vs Forwards-Energy | -0.17723 | 0.0063 | 2847828 | -28.14 | <.0001 |
| Forwards-Confusion vs Random-Selection | -0.11733 | 0.00635 | 2847828 | -18.481 | <.0001 |
| Sideways-Confusion vs Forwards-Energy | -0.12061 | 0.00597 | 2847828 | -20.191 | <.0001 |
| Sideways-Confusion vs Random-Selection | -0.06071 | 0.00603 | 2847828 | -10.074 | <.0001 |
| Forwards-Energy vs Random-Selection | 0.0599 | 0.00624 | 2847828 | 9.593 | <.0001 |

# 4. Behavioural validation of motion confusion measures for butterflies

## 4. i. Summary

To test whether or not our motion confusion metrics could predict changes in predator attack behaviour, we constructed a high-framerate (240hz) touch screen butterfly capture game using the 3D simulations of butterflies from sections 2 and 3. This involved the following: subselecting five butterflies from Collins ' Guide to Butterflies that were representative of different levels of motion confusion; rendering the butterflies onto 10 different flight paths to create animations for a touch-screen game; and recruiting 100 volunteers to collect data on how motion confusion measures prior to the player touching the screen influenced where they clicked relative to the butterfly.

## 4. ii. Butterfly species

For the game, we sub-selected 5 butterfly phenotypes from the range of Collins Guide to Butterflies. These butterflies were selected from across the motion confusion gradient for forwards- and sideways-confusion.

The phenotypes were as follows, from highest to lowest level of forwards-confusion (See Supp Table.13 and Supp Fig.24):

*Brintesia circe (Male) - B.c.*

*Papilio alexanor* (Female) - P.a. *NB this species has the highest sideways confusion*

*Pyrgus sidae (Male) - P.s.*

*Ginepteryx cleobule (Male) - G.c.*

*Lycaena tityrus* (Female) - L.t.

**Supplementary Table 13** **| Selected butterfly phenotype, the butterfly’s family and their motion confusion measures.** Colour images of butterflies were used from the Collins Butterfly Guide with permission from Richard Lewington.

| ***Phenotype*** | **Family** | **Forwards Confusion** | **Sideways Confusion** | **Forwards Energy** |
| --- | --- | --- | --- | --- |
| *Brintesia circe*  (Male)   | Nymphalidae | -0.37 | 0.14 | 0.10 |
| *Papilio alexanor*  (Female)   | Papilonidae | -0.42 | 0.28 | 0.07 |
| *Pyrgus sidae*  (Male)   | Hesperiidae | -0.62 | 0.004 | 0.05 |
| *Ginepteryx cleobule*  (Male)   | Pieridae | -0.64 | -0.07 | 0.09 |
| *Lycaena tityrus*  (Female)   | Lycanidae | -0.66 | -0.15 | 0.11 |

**Supplementary Fig.24 | Forwards and sideways confusion for 5 selected butterflies,** shows the forwards and sideways confusion measures relative to other butterflies. Blue-line shows the linear correlation between sideways- and forwards-confusion while the points represent the values for the other butterflies. Images of butterfly wings were used from the Collins Butterfly Guide with permission from Richard Lewington.

## 4. iii. Butterfly animations and flight behaviour

*The scripts used to create the flight paths and to animate the butterflies along the paths are included in BF_Data_Repository/Analysis_04_Game/Blender_Files/.*

As with the Collins and GA simulations of butterflies, we used Blender to create artificial renderings of butterflies. Butterflies were rescaled so that they had the same wing area. Butterflies had a mean body length of 2.42cm when rendered on the screen. Wing beat frequencies were adjusted based on body size and wing area, as with our previous simulations. However, unlike the previous simulations, butterflies were animated with an average flight speed of ~40cm/second for a period of 1.458 seconds, with multiple wing beat cycles per flight and sinusoidal flight paths, to simulate natural flight behaviour and make them more difficult to capture. Butterflies initiated their flight from the bottom left corner of a 1280 by 720 px background image, which was the same colour as the previously used backgrounds (87,87,87 RGB) and would then fly off the screen either from near the bottom right or top right corners. The start position of the butterflies was offset from the centre point in a 50° arc (± 25°) centred on 45° from the left corner of the background (See Supp Fig.25).

**Supplementary Fig.25 | Butterfly path generation illustration.,** Butterflies start in a position randomly offset in a 50 degree arc from the start position. White dashed line shows the arc of butterfly start positions, black line shows an example flight path.

Sinusoidal flight was encoded by first determining the start and end positions of the butterfly and the heading required.

*Start Position = r_0_ = (x_0_ , y_0_)*

*End Position = r_1_ = (x_1_ , y_1_)*

*Path Vector = d = (x_1_− x_0_ , y_1_−y_0_)*

*Base Heading = θ_0_ =atan2(d)*

*Path length =∥d∥*

*Duration = T*

*Speed = v = L/T*

Sine oscillations were then overlaid on top of the path in two forms. The first was a sine function that influenced the heading of the butterfly. This used two sine functions with different offsets in phase; the second function was absoluted and multiplied by the first to create variations in amplitude of the wave across the flight.

E.g. Python Code

*heading_sin_amp_deg	= random.uniform(30.0, 40.0)*

*heading_sin_freq	= random.uniform(1.0, 2.5)*

*heading_phase_offset_1 = random.uniform(0, 2*math.pi)*

*heading_phase_offset_2 = random.uniform(0, 2*math.pi)*

*sin_str = abs(math.sin(0.5 * math.pi * heading_sin_freq * time_sec + heading_phase_offset_2))*

*heading_offset = sin_str * heading_sin_amp * math.sin(2 * math.pi * heading_sin_freq **

*time_sec + heading_phase_offset_1)*

*heading = base_heading + heading_offset*

*x += math.cos(total_heading) * avg_speed*

*y += math.sin(total_heading) * avg_speed*

Then an additional wave that only influenced the x and y coordinate and not the heading was overlaid on top of the x and y position to simulate subtle non-heading orientated movements, such as those generated by the wind or movements perpendicular to the heading of the animal, which butterflies and various other animals are capable of. This would have resulted in both variations in speed and in lateral position.

E.g. Python Code

*pos_phase_offset = rand["pos_phase_offset"]*

*x += rand["pos_sin_amp"] * math.sin(2 * math.pi * rand["pos_sin_freq"] * time_sec +*

*pos_phase_offset)*

*y += rand["pos_sin_amp"] * math.cos(2 * math.pi * rand["pos_sin_freq"] * time_sec +*

*pos_phase_offset)*

We generated 50 random flight paths within the parameter range for heading and positional waves set above. We then subselected 5 paths where the endpoint was in the top right corner and 5 where the endpoint was in the bottom left corner. The 5 selected paths for each end point were uniformly selected from low to high levels of linearity (inverse of the level of displacement from the straight line path from start to end). The settings of each of these paths were saved as .json files, which could be reloaded using Blender and Python, allowing the butterfly phenotypes to be mapped to the same motion paths. See Supp Fig.26 for illustrations of each flight path.

**Supplementary Fig.26 | Flight paths,** shows the 10 unique flight paths used for the butterfly game. The number assigned to each flight path is shown above the path. Absolute values greater than 1 are outside of the bounds of the screen. Dashed lines indicate the centre position of the screen. By default, butterflies start at the bottom left and end on the right side of the screen. During the actual game, the stimuli were randomly flipped on the x and y axes, meaning the butterfly could start from any corner of the screen. Whether the path ends on the same vertical region of the screen as it started or on the opposite end is indicated using the colour of the line.

When animated along each path, butterflies were coded to bank perpendicularly to their heading when turning. This was done to emulate how real butterflies orient as they change heading and to make the flights more dynamic. The degree to which the butterfly banked was dependent on the angular change in heading, with more extreme changes resulting in steeper bankings. See supplementary video 4.

## 4. iv. Rendering and EMD measurement

Butterfly videos were rendered at a frame rate of 1000fps and then underwent temporal motion smoothing with the same Gaussian blur levels used for the EMD analyses. As birds see faster than humans, and pilot experiments found humans struggled to catch butterflies moving faster than 50cm/second, we opted to create targets that flew across the screen at 40cm/second, but had the equivalent wingbeat frequency and blur to a butterfly travelling at double the speed 80cm/second, which was in the range of speeds for the free-flying butterfly take-offs. This was achieved by resampling the number of frames and the dividing the wingbeat frequency by two for the Blender render prior to applying the blurring method (See Supp Fig.27).

**Supplementary Fig.27 | Example butterfly renders,** singular frame for *Brintesia circe* showcasing the original Blender rendered frame, the spatio-temporally smoothed frame and the hitbox for the frame.

After applying temporal smoothing, we downsampled the number of frames to match the frame rate of the monitor, 240 fps, for the games(ee section 4.v.) For each flight path and each phenotype, a second video was rendered using a labelled hitbox created for the butterfly’s image. This hitbox was colour-coded such that the red channel could be used to indicate whether a butterfly was hit (butterfly = 255 R), and the green channel could indicate which region of the butterfly was hit (body = 10 G, forewing = 200 G, hindwing = 255 G, or tail = 30 G).

For each butterfly and for each path, we measured EMD using the same method of alignment as the butterflies filmed in free flight. The images were re-oriented so that the start and end points were in a straight vertical line upwards. The forwards (up), backwards (down), left, and right EMD were then used to calculate the confusion measures (forwards and sideways confusion) for the butterflies. This gave us the EMD and confusion measures for every frame. As confusion varied throughout the flight for each butterfly due to differences in wingbeat cycle and heading (See supp Fig.28), we created a measure for the mean EMD values and confusion values starting from 400 milliseconds (96 frames) before each frame. Though it is worth noting that changes in the orientation of the butterfly will have resulted in noise in the motion confusion measures, as they aren’t always aligned with the direction of movement. For instance, forwards-confusion was higher for P.a. than B.c. on average, likely due to movements perpendicular to the start and end heading.

**Supplementary Fig.28 | EMD measures of game flight paths., a.** Forwards Confusion, **b,** Sideways Confusion, **c,** Log Forwards Energy. Colour images of butterflies were used from the Collins Butterfly Guide with permission from Richard Lewington.

## 4. v. Butterfly game

For the experiment, we recruited 100 anonymous volunteer players from across the University of Exeter’s Penryn Campus (Penryn, Cornwall, UK, TR10 9FE) to play a touch-screen-based butterfly-catching game. Volunteers were recruited via email and by signs, with ethical approval from the University of Exeter ethics committee (REF: 9474868). The game was run using an ASUS Aspire Nitro V15 laptop [ASUS Computer International, 48720 Kato Road, Fremont, CA 94538, United States] with an inbuilt nvidia GEFORCE RTX graphics card, allowing us to display the game with a secondary screen for the experiment, a GPU-powered 240hz ASUS XG32UCWMG gaming monitor. The screen for the experiment was 71cm wide and 40cm high, with a maximum resolution of 1920 x 1080 when in enhanced frame rate mode. While the uppermost framerate of the screen was 480 hz, we selected a vertical refresh rate of 240 hz for increased stability.

To convert the screen into a touchscreen, we used a greentouch infra-red (IR) frame, placed over the monitor and with a thin (2mm) thick acrylic sheet cut to fit behind it to protect the screen. IR frames allow objects, i.e. a participant's finger, that penetrate the frame to be registered as clicks (See Supp Fig.29). IR frames are advantageous compared to alternative touch device methods due to their high response rate >240 hz and their ability to be paired with almost any screen[^1,2^](https://www.zotero.org/google-docs/?5s9Kjr). Players were seated on a chair positioned ~50cm away from the screen and were asked to adjust the height of the screen and to adjust themselves to where they could comfortably reach all four corners of the screen without fully extending their arm, and so that their head faced the centre point of the screen. The mean body length of the butterflies was 2.42cm , giving an angular width of ~2.80° and an angular speed of ~58.99° per second.

**Supplementary Fig.29 | Behavioural Experiment Setup., a.** Arrangement of infrared (IR) touch frame, protective acrylic sheet and computer screen. **b.** Position of the player relative to the screen.

To create the game, we used MATLAB R2025 along with the Psychtoolbox-3 and a suite of custom functions for initialising the screen and Psychtoolbox and for registering the touch output as clicks, dubbed PECK as they were initially developed for experiments using birds[^3^](https://www.zotero.org/google-docs/?snGcsM). As PECK has not yet been released a stripped version of it including only functions necessary for the experiment has been provided within the supp “/Analysis_04_Game/Game_Environment/”.

Each player undertook 30 trials rendered at 240 hz with six replicates of each butterfly phenotype. The trials used random flight paths, and each path could be flipped on the x and/or y axis. This gave a total of 3000 trials across 100 participants. Before each trial, the player was first instructed to click on a white circle positioned in the same corner from which the butterfly would start. This meant the player's finger always started behind the butterfly. The butterfly would then immediately appear, and the player would have to attack it using a single finger. Once the player touched the screen, the butterfly would pause, and text would appear revealing whether the player had successfully hit or if they had missed the butterfly. If the butterfly's hitbox was within a 30 pixel radius of the click, it was considered a hit. This was to account for the size of the participant's finger.

Before the experiment commenced, the player was given a series of instructions teaching them how to play the game and asking them to use only their dominant hand and one finger when playing the game. Players also did a short tutorial to make sure that they knew what they were doing and that they could catch the butterflies at the speed they travelled for the experiment. This tutorial operated by tasking the player to try and click on butterflies of three increasing speeds, 0.5x, 0.75x and 1.0x the speed of the butterfly for the experiment. To progress to the next speed, the player needed to successfully catch two butterflies at that speed. Players on average took 14 ± 6 std.dev trials to complete the tutorial. Additionally, during the tutorial, when the player clicked the screen, they were also given information about whether their click was to the front or back and left or right of the butterfly. These trials did not count towards the 30 trials used for the actual experiment.

*For an example of someone playing the experiment, see Supp Video 4.*

## 4. vi. Data collection and statistical analyses

For each trial, we recorded the unique ID assigned to the player, the flight path and butterfly phenotype used, the order number of the trial, whether the path was flipped on the x and/or y axis, the coordinates and frame of the touch/click if there was one and the RGB values of the nearest pixel of the hitbox to the click. By using the heading of the butterfly, we were able to calculate the angle of the click relative to the butterfly and the distance of the click on different vectors, the distance parallel to the butterfly’s (y vector) heading and the distance perpendicular (x vector). As click distances were defined relative to the butterfly headings, the y vector was allowed to be positive or negative, with negative values indicating clicks behind the centre of the butterfly and positive values being in front.

To test whether or not our motion confusion measures influenced how the butterflies clicked relative to the butterflies, we used a linear mixed model (lme4 package)[^4^](https://www.zotero.org/google-docs/?mWxMtT), with the vector distance as the response variable and the axis (x or y) and the predicted motion confusion measures, forwards-confusion, sideways-confusion, and the forwards energy, of the butterfly as the predictor variables, with the player ID, the trial order and the path ID as random effects, in addition to frame rate as during some trials frame-rate dropped to ~220hz. For the model, we included interactions between the axis and each confusion measure. Our expectations were that forwards-confusion should influence the click distance parallel to the butterfly (y) and that sideways-confusion should influence the click distance perpendicular (x).

In addition to the linear model, we used 2D kernel density estimation with the Mass package for our vectors to determine how the butterfly phenotypes influenced the shape of the distribution of clicks[^5^](https://www.zotero.org/google-docs/?2qOduJ). We extracted the grid for the top 25% densest regions of clicks for each butterfly and used ellipse statistics (centroid coordinates, angle, aspect ratio, major and minor width) to describe the shape of where players tended to click. We also used a binomial generalised linear mixed model with the same random effects as the linear model to test whether EMD measures influenced the likelihood of clicking forewing or hindwing. Clicks to the body and misses were excluded from this analysis.

For each model, continuous predictor and response variables were re-scaled to a mean of 0 and a standard deviation of 1.

## 4. vii. Results

*Click Information*

Across the experiment, we collected data from 3,000 trials (30 trials x 100 players). Of these trials, 2,985 (99.5%) included clicks to the screen, and 1,182 had clicks within 30 pixels of the butterfly’s hitbox (1.1cm).

*Influence on click position*

Across trials, the majority of clicks took place behind the butterflies with a mean click distance of -77.6 pixels ± 72.5 std.dev (-2.8cm, 1.2 x the average body length of the butterflies) parallel to the butterfly, with comparable distances perpendicular to the butterfly 79.1 pixels ± 56.0 std.dev. Given the erratic flight of the butterflies the click distributions were unsurprisingly quite noisy.

All three of our confusion measures were found to significantly influence the click position relative to the butterfly (p<0.05, see Supp Table 14). Forwards-confusion caused attacks to be further to the side (perpendicular, x) and to the back of the butterflies, while forwards energy also caused attacks to be further to the back of the butterfly, but did not influence the distance perpendicular to the butterfly. Sideways confusion resulted in attacks being further forward and closer to the body of the butterfly. Based on the simulated flight data, this may be expected as a byproduct of strong selection for forwards confusion, given their tight correlation and greater explanatory power of forwards confusion to predict real butterfly wing patterning

**Supplementary Table 14| Linear mixed model for motion confusion measures. Note, response and predictor variables were rescaled so estimates do not reflect actual distances.**

| **Variable** | **Estimate** | **SE** | **DF** | **T ratio** | **P value** |
| --- | --- | --- | --- | --- | --- |
| Intercept | 0.42 | 0.06 | 10.94 | 7.14 | <0.001 |
| Axis = Y | -0.83 | 0.02 | 5824.98 | -36.51 | <0.001 |
| Mean Forwards-Confusion 400ms from Click | 0.06 | 0.03 | 5929.31 | 2.13 | 0.033 |
| Mean Sideways-Confusion 400ms from Click | -0.09 | 0.04 | 5945.90 | -2.30 | 0.022 |
| Mean Forwards Energy 400ms from Click | 0.02 | 0.03 | 5898.96 | 0.54 | 0.587 |
| Y : Forwards-Confusion | -0.15 | 0.04 | 5824.98 | -3.92 | <0.001 |
| Y : Sideways-Confusion | 0.23 | 0.05 | 5824.98 | 4.24 | <0.001 |
| Y : Forwards Energy | -0.13 | 0.04 | 5824.98 | -3.32 | <0.001 |

Comparisons of the ellipses for the denser regions (top 25%) of the click kernel densities of the butterflies revealed further variation in the click distributions (See Supp Table 15 and Supp Fig.30). Attacks against species with higher motion confusion tended to be further backwards barring (P.s) and had a narrower aspect ratio with attacks oriented backwards (+90°).

Notably for the swallowtail (P.a), attacks were concentrated towards the tail, supporting the hypothesis that swallowtail butterflies use their tails as an anti-predator defence. Like the tails of some lizards, swallowtail tails can break off when attacked allowing the butterfly to escape.

**Supplementary Table 15 | Ellipse shape statistics given in pixels, above, and cm, below. Centroid x and y denote the centre point of the ellipse. The major and minor axes are the longest diameter and the shorter perpendicular diameter, respectively; the aspect ratio is the major/minor axis; and the angle is the orientation of the major axis. The average body length of a butterfly was 2.42cm. Butterflies are ordered by forwards-confusion.**

| **Pixels (px)** | | | | | | |
| --- | --- | --- | --- | --- | --- | --- |
| **Butterfly** | **centroid_x** | **centroid_y** | **major_axis** | **minor_axis** | **aspect_ratio** | **angle (°)** |
| L.t | 57.54 | -57.02 | 76.90 | 66.39 | 1.16 | 37.82 |
| G.c | 53.20 | -80.37 | 66.21 | 60.10 | 1.10 | -39.41 |
| P.s | 44.29 | -73.78 | 76.51 | 61.13 | 1.25 | 97.24 |
| P.a | 52.02 | -82.00 | 75.20 | 60.32 | 1.25 | 113.65 |
| B.c | 43.51 | -80.23 | 81.74 | 50.70 | 1.61 | 86.73 |
| **Centimetres (cm)** | | | | | | |
| L.t | 2.10 | -2.08 | 2.80 | 2.42 | 1.16 | 37.82 |
| G.c | 1.94 | -2.93 | 2.41 | 2.19 | 1.10 | -39.41 |
| P.s | 1.61 | -2.69 | 2.79 | 2.23 | 1.25 | 97.24 |
| P.a | 1.90 | -2.99 | 2.74 | 2.20 | 1.25 | 113.65 |
| B.c | 1.59 | -2.93 | 2.98 | 1.85 | 1.61 | 86.73 |

**Supplementary Fig.30 | Butterfly click distributions,** enlarged version of the Fig 2c. Crosses and ellipses show the shape statistics for the 25% densest regions of each butterfly, with the crosses indicating the centroid and the ellipses showcasing the orientation, size and aspect ratio. Butterfly outlines showcase the to-scale butterfly when the wings were fully extended during flight. Images of butterflies and their wing patterns were used from Collins Butterfly Guide with permission from Richard Lewington.

*Influence of phenotype on the hitbox hit*

Despite the majority of attacks landing behind the butterfly, the majority of hits for most butterflies were on the forewings (See Supp Fig.31), except for the swallowtail butterfly (P.a.), where the majority of hits were on the hindwing, especially the tail. Using the binomial model with Tukey post hoc comparisons, only the swallowtail was significantly different from the other butterflies with a greater frequency of hindwing hits than any other species (See Supp Table 16).

**Supplementary Fig.31 | Hitbox hit, a.** Ratio of hits for each hitbox, note: only the swallowtail (P.a.) had a tail hitbox. **b.** Ratio of forewing to hindwing hits, note tail hits here are encoded as hindwing and body hits are excluded. Colour images of butterflies were used from the Collins Butterfly Guide with permission from Richard Lewington.

**Supplementary Table 16 | Tukey post hoc comparisons for binomial model of forewing vs hindwing hits. Negative values indicate more forewing hits and positive values indicate more hindwing hits then the point of comparison. E.g. L.t has more forewing hits than P.a.**

| **Contrast** | **estimate** | **SE** | **z.ratio** | **p.value** |
| --- | --- | --- | --- | --- |
| L.t vs G.c | -0.39 | 0.20 | -1.92 | 0.30 |
| L.t vs P.s | -0.26 | 0.20 | -1.33 | 0.67 |
| L.t vs P.a | -1.35 | 0.21 | -6.55 | <0.001 |
| L.t vs B.c | -0.46 | 0.19 | -2.36 | 0.13 |
| G.c vs P.s | 0.12 | 0.20 | 0.61 | 0.97 |
| G.c vs P.a | -0.97 | 0.21 | -4.63 | <0.001 |
| G.c vs B.c | -0.07 | 0.20 | -0.36 | 1.00 |
| P.s vs P.a | -1.09 | 0.21 | -5.24 | <0.001 |
| P.s vs B.c | -0.20 | 0.20 | -1.00 | 0.86 |
| P.a vs B.c | 0.89 | 0.20 | 4.44 | <0.001 |

*Influence of phenotype on hit rate*

For the experiment, all butterflies were rescaled to have the same wing pixel area. This ensured they all had equivalent visual areas and sizes. However, due to differences in the wing shape of the butterflies, the bounding area of the butterfly and the area that was hittable (given the 30px radial expansion of the click coordinate) varied. For instance, the European swallow tail (P.a). had on average a larger bounding area then all other butterflies (Supp Fig.32). This was due to the narrower wing tips and tails increasing the bounds of the butterfly. In contrast, species with flatter wing edges, such as G.c and B.c, had smaller bounding areas.

**Supplementary Fig.32 | Butterfly hitbox bounding area,** boxplots with violons showing the differences in distribution of bounding hitbox area across frames between the butterfly phenotypes. Colour images of butterflies were used from the Collins Butterfly Guide with permission from Richard Lewington.

We found no significant effect of butterfly type on the frequency of hits, regardless of whether hitbox area was accounted for, though L.t was clicked on average more so than other butterflies and G.c. the least (Fig. 33a and Supp Table 17). Furthermore, having a larger bounding area at the time of click did increase the chance of a hit.

However, when the tail was excluded from hits, P.a. was the hardest to hit with a significantly lower hit probability then all other butterflies (Fig. 33 and Supp Table 18). This was due to the high proportion of attacks directed towards the tail: 36% (Fig.31).

**Supplementary Fig.33 | Hitbox hits with and without tail hits,** shows the proportions of hits for each butterfly with and without tail hits included. Colour images of butterflies were used from the Collins Butterfly Guide with permission from Richard Lewington.

**Supplementary Table 17 | Model output for click hit success for each butterfly when tail hits are counted as hits, with butterfly type and scaled hitbox area as predictors and binary hit or miss as the response.**

| **Linear Model with Butterfly Tails** | | | | |
| --- | --- | --- | --- | --- |
| **Variable** | **Estimate** | **SE** | **Z value** | **P value** |
| Intercept | -0.45 | 0.30 | -1.52 | 0.13 |
| Butterfly = G.c | -0.15 | 0.13 | -1.17 | 0.24 |
| Butterfly = P.s | -0.13 | 0.13 | -1.01 | 0.31 |
| Butterfly = P.a | 0.05 | 0.13 | 0.37 | 0.71 |
| Butterfly = B.c | -0.15 | 0.13 | -1.18 | 0.24 |
| scale(hitbox_bound area) | 0.38 | 0.05 | 8.26 | <0.001 |

| **Posthoc comparisons with Butterfly Tails** | | | | |
| --- | --- | --- | --- | --- |
| **Contrast** | **Estimate** | **SE** | **z.ratio** | **p.value** |
| L.t vs G.c | 0.15 | 0.13 | 1.17 | 0.77 |
| L.t vs P.s | 0.13 | 0.13 | 1.01 | 0.85 |
| L.t vs P.a | -0.05 | 0.13 | -0.37 | 1.00 |
| L.t vs B.c | 0.15 | 0.13 | 1.18 | 0.76 |
| G.c vs P.s | -0.02 | 0.13 | -0.18 | 1.00 |
| G.c vs P.a | -0.20 | 0.14 | -1.46 | 0.59 |
| G.c vs B.c | 0.00 | 0.13 | -0.02 | 1.00 |
| P.s vs P.a | -0.18 | 0.13 | -1.36 | 0.66 |
| P.s vs B.c | 0.02 | 0.13 | 0.17 | 1.00 |
| P.a vs B.c | 0.20 | 0.13 | 1.49 | 0.57 |

**Supplementary Table 18 | Model output for click hit success for each butterfly when tail hits are not counted as hits, with butterfly type and scaled hitbox area as predictors and binary hit or miss as the response.**

| **Linear Model without Butterfly Tails** | | | | |
| --- | --- | --- | --- | --- |
| **Variable** | **Estimate** | **SE** | **Z value** | **P value** |
| Intercept | -0.45 | 0.30 | -1.50 | 0.13 |
| Butterfly = G.c | -0.17 | 0.13 | -1.32 | 0.19 |
| Butterfly = P.s | -0.13 | 0.13 | -1.03 | 0.30 |
| Butterfly = P.a | -0.75 | 0.14 | -5.37 | <0.001 |
| Butterfly = B.c | -0.17 | 0.13 | -1.32 | 0.19 |
| scale(hitbox_bound area) | 0.33 | 0.05 | 7.07 | <0.001 |

| **Posthoc comparisons without Butterfly Tails** | | | | |
| --- | --- | --- | --- | --- |
| **Contrast** | **estimate** | **SE** | **z.ratio** | **p.value** |
| L.t vs G.c | 0.17 | 0.13 | 1.32 | 0.68 |
| L.t vs P.s | 0.13 | 0.13 | 1.03 | 0.84 |
| L.t vs P.a | 0.75 | 0.14 | 5.37 | <0.001 |
| L.t vs B.c | 0.17 | 0.13 | 1.32 | 0.68 |
| G.c vs P.s | -0.04 | 0.13 | -0.31 | 1.00 |
| G.c vs P.a | 0.57 | 0.15 | 3.91 | <0.001 |
| G.c vs B.c | 0.00 | 0.13 | -0.04 | 1.00 |
| P.s vs P.a | 0.62 | 0.14 | 4.43 | <0.001 |
| P.s vs B.c | 0.04 | 0.13 | 0.28 | 1.00 |
| P.a vs B.c | -0.58 | 0.14 | -4.10 | <0.001 |

# References

[1.](https://www.zotero.org/google-docs/?YVTHFD)	Hancock, George RA, et al. "Biologically inspired warning patterns deter a passerine, Parus major, from digital turbine blades." Behavioral Ecology 37.4 (2026): arag039.

[2.](https://www.zotero.org/google-docs/?YVTHFD)	[Silvasti, S. *et al.* The flashy escape: support for dynamic flash coloration as anti-predator defence. *Biol. Lett.* **20**, (2024).](https://www.zotero.org/google-docs/?YVTHFD)

[3.](https://www.zotero.org/google-docs/?YVTHFD)	[Navracsics, J. & Darzhinova, L. The Use of PsychToolbox-3 for Tracking Bilingual Sentence Processing and Comprehension. in 123–131 (2020).](https://www.zotero.org/google-docs/?YVTHFD)

[4.](https://www.zotero.org/google-docs/?YVTHFD)	[Bates, D. M. lme4: Mixed-effects modeling with R. (2010).](https://www.zotero.org/google-docs/?YVTHFD)

[5.](https://www.zotero.org/google-docs/?YVTHFD)	[Ripley, B. *et al.* Package ‘mass’. *Cran R* **538**, 822 (2013).](https://www.zotero.org/google-docs/?YVTHFD)
